# Evidence of a 4.33 billion year age for the Moon’s South Pole–Aitken basin

**DOI:** 10.1038/s41550-024-02380-y

**Published:** 2024-10-16

**Authors:** K. H. Joy, N. Wang, J. F. Snape, A. Goodwin, J. F. Pernet-Fisher, M. J. Whitehouse, Y. Liu, Y. T. Lin, J. R. Darling, P. Tar, R. Tartèse

**Affiliations:** 1 Department of Earth and Environmental Sciences, The University of Manchester, Manchester, UK; 2 Institute of Geology and Geophysics, Chinese Academy of Sciences, Beijing, China; 3 Department of Geosciences, Swedish Museum of Natural History, Stockholm, Sweden; 4 School of the Environment, Geography and Geosciences, University of Portsmouth, Portsmouth, UK; 5 Previously at the School of Health Sciences, Faculty of Biology, Medicine and Health, The University of Manchester, Manchester, UK

**Keywords:** Meteoritics, Mineralogy, Petrology, Inner planets

## Abstract

The Moon’s farside South Pole–Aitken (SPA) basin is the largest and oldest visible impact basin in the inner Solar System. Determining the timing of this catastrophic event is key to understanding the onset of the lunar basin-forming epoch, with implications for understanding the impact bombardment history of the inner Solar System. Despite this, the formation age of the SPA basin remains poorly constrained. Here we show that the chemical composition of the lunar meteorite Northwest Africa 2995 is in good agreement with lithologies exposed within the southern region of the SPA basin. Radiometric dating of a range of mineral and rock components in Northwest Africa 2995 yielded consistent dates of ~4.32–4.33 billion years old. We interpret these dates as the age of SPA basin formation, inferring that this event occurred ~120 million years before the formation of the main cluster of lunar impact basins between ~4.2 Ga and 3.8 Ga. This weakens support for a narrow period of lunar late heavy impact bombardment and also implies that the earliest formed impact basins on the Moon (that is, >4.33–4.5 Ga old) were erased either by the SPA impact itself when its formation caused massive resurfacing of the lunar surface or through other geological processes.

## Abstract (web summary)

A lunar meteorite sample called Northwest Africa 2995 probably originated from the South Pole–Aitken basin on the Moon’s farside. Minerals in the meteorite date basin formation to 4.32–4.33 billion years ago.

## Main

The Moon was heavily bombarded by impacting asteroids, comets and planetesimal debris before 3.8 billion years ago^1–6^. The scars of these collisions have shaped the lunar surface, creating the giant impact basins (>150 km) and smaller impact craters, generating localised superheated piles of melt and distributing ejecta material across the Moon’s surface^7^. Determining the timing and order of lunar basin formation is key to our understanding of the nature and timing of early Solar System dynamical processes^2,3,6^. The South Pole–Aitken (SPA) basin, with inner ring dimensions of ~2,000 × 1,500 km (ref. ^8^), is the largest impact basin with a surface expression on the Moon and on all the terrestrial planetary bodies. The date of the basin-forming event, thus, establishes the onset of lunar basin formation^6^. The SPA basin-forming event is thought to have excavated material from the lower crust and upper mantle^9,10^ and probably formed a large melt pile within the basin, which differentiated into different types of igneous-like rock^11,12^. The impact event not only shaped the crustal and volcanic history of the lunar farside^10,13,14^ but may have also caused large-scale instability within the lunar interior triggering partial melting on the opposite side (that is, nearside) of the Moon^15^.

Samples have recently been collected from the SPA basin by the Chang’e–6 robotic mission, and some mafic (that is, olivine-norite) impact melt rocks were also chemically investigated in situ in the SPA basin by the Chang’e–4 rover^16–18^. In addition to the newly collected Chang’e–6 samples, it is likely that recent impactors could have ejected SPA material into Earth-crossing orbits, meaning that we could have naturally delivered SPA-derived lunar meteorites here on Earth. Indeed, several lunar meteorites have previously been tentatively linked to an SPA basin origin on the basis of their compositional similarity to SPA basin surficial lithologies, and/or unique compositions compared with Apollo samples^19,20^. The lunar meteorite Northwest Africa (NWA) 2995 is one such sample that has been proposed to have originated within SPA^19,21^. Here, we test this hypothesis and demonstrate how its rock and mineral records do match a SPA basin origin, and date its formation at ~4.32–4.33 Ga.

## Results

### Petrography

**Fig. 1:**
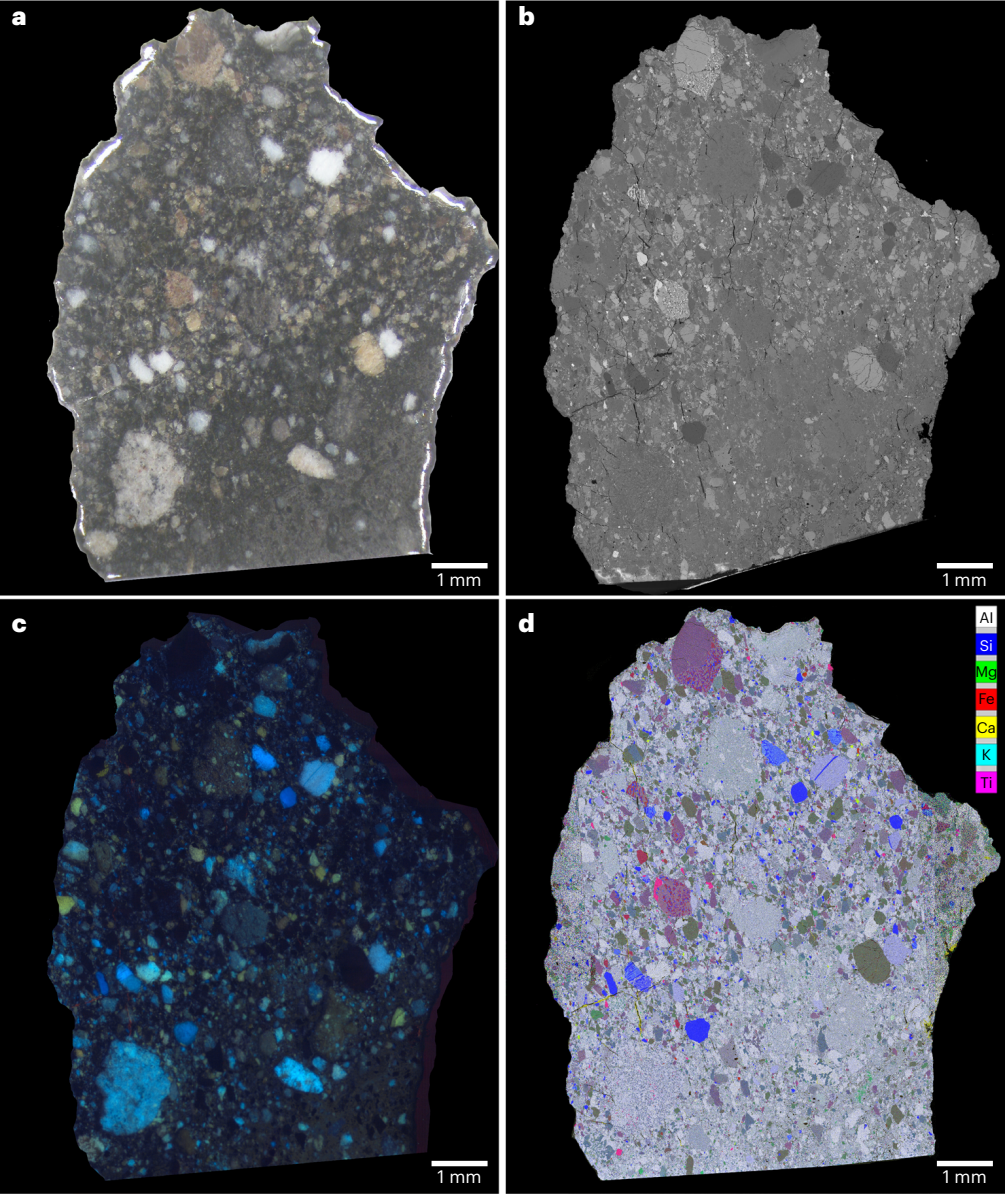
Overview of the NWA 2995 lunar meteorite sample. **a**, An optical scan of the polished sample surface showing a range of coloured clasts in a dark matrix. **b**, A BSE image (note that the lower part of the sample image was truncated during data collection). **c**, A CL image. **d**, A composite false colour element map (combining two different maps) where the colours represent silica (blue), aluminium (white), magnesium (green), iron (red), titanium (pink), potassium (cyan) and calcium (yellow). A map showing the location of clasts and phases of interest can be found in Supplementary Fig. 1.

NWA 2995, found in Algeria in 2005, is one of a clan of several grouped lunar meteorite stones that include NWA 2996, 3190, 4503, 5151, 5152, 6252, 6554, 6555 and so on^22^. The sample is a breccia with a dark-grey matrix, enclosing white, brown and grey clasts up to 1.5 mm in size (Fig. 1). Clasts in NWA 2995 consist of feldspathic impact melt breccias with different textures including clast-bearing and dendritic crystalline types, anorthositic gabbronorites, non-mare mafic clasts, granulitic breccias, exsolved pyroxene, Si- and K-rich granophyres, glass fragments and mineral fragments (Fig. 1 and Supplementary Figs. 1–4). The mineral chemistry of these components supports a lunar origin (Supplementary Fig. 5 and Supplementary Datasets 1–7). Studies of other stones in the NWA 2995 clan have identified rare very low-Ti olivine basalts^23^ and magnesian anorthositic clasts^24^ that are atypical in composition compared with the Apollo highland rock suites^21,22^.

Most lithic and mineral clasts in NWA 2995 are fractured and display evidence of brittle deformation during disaggregation of their precursor rocks; plagioclase clasts have undergone a range of shock pressures from unshocked (<5 GPa; equivalent to shock state S1 ‘unshocked’) to moderately shocked (26 ± 3 GPa; equivalent to shock state S4 ‘strongly shocked’)^25^. The occurrence of glass spherules in NWA 2995 (Supplementary Fig. 2d) and in paired sample NWA 2996 (ref. ^21^) confirms that the meteorite is a regolith breccia representing an ancient fused lunar soil, made up of many different rock and mineral components.

Although the meteorite is heavily brecciated, some of the exsolved pyroxene and granophyre clasts have shared mineral grain boundaries (Supplementary Figs. 3 and 4) and are part of the same quartz monzogabbro (QMG) evolved lithology. Pyroxene in these QMG clasts have a range of Mg# (Supplementary Fig. 6a and Supplementary Dataset 3) and plot over a range of Ti# versus Fe# trends (Supplementary Fig. 6b), suggesting they originated from slightly compositionally distinct parent melts (that is, low-Ti to high-Ti like). These pyroxene grains are exsolved, typically displaying 1–5 µm augitic lamellae hosted in pigeonite, although the opposite relationship sometimes occurs (Supplementary Fig. 3). Clinopyroxene compositions suggest equilibration closure temperatures of 748–786 °C (Supplementary Dataset 8), similar to equilibration temperatures previously calculated for exsolved pyroxene in other types of lunar monzogabbro and quartz monzodiorite^26,27^. Plagioclase in QMG clasts include both alkali-rich and K-feldspar species, less commonly Ca-rich plagioclase (Supplementary Fig. 7). These feldspars are often intergrowth with a Si-rich phase (Supplementary Fig. 4), which Raman spectroscopy indicates is either quartz or amorphous silica, with no other Si-mineral polymorphs observed (Supplementary Fig. 8 and Supplementary Table 9). Analysis of silica phases in these clasts and in the matrix indicates a range of Ti abundances (QMG clasts 240–1,204 µg g^−1^, monomineralic matrix grains 820–2,410 µg g^−1^; Supplementary Dataset 7), suggesting a range of melt Ti saturation conditions^28^. Some QMG clasts have areas of late-stage olivine–pyroxene–silica intergrowths (Supplementary Fig. 3a). Minor mineral phases include ilmenite and troilite, and small 10–20-µm-scale pockets of Si–Al–K-rich glass. Both QMG and granophyre clasts include Ca-phosphate phases. Some zircon grains found in the meteorite matrix (Supplementary Figs. 9–11) are also associated with QMG-sourced exsolved pyroxene grains (that is, these phases share a crystal boundary; Supplementary Fig. 10).

### Phosphate and zircon dating

**Fig. 2:**
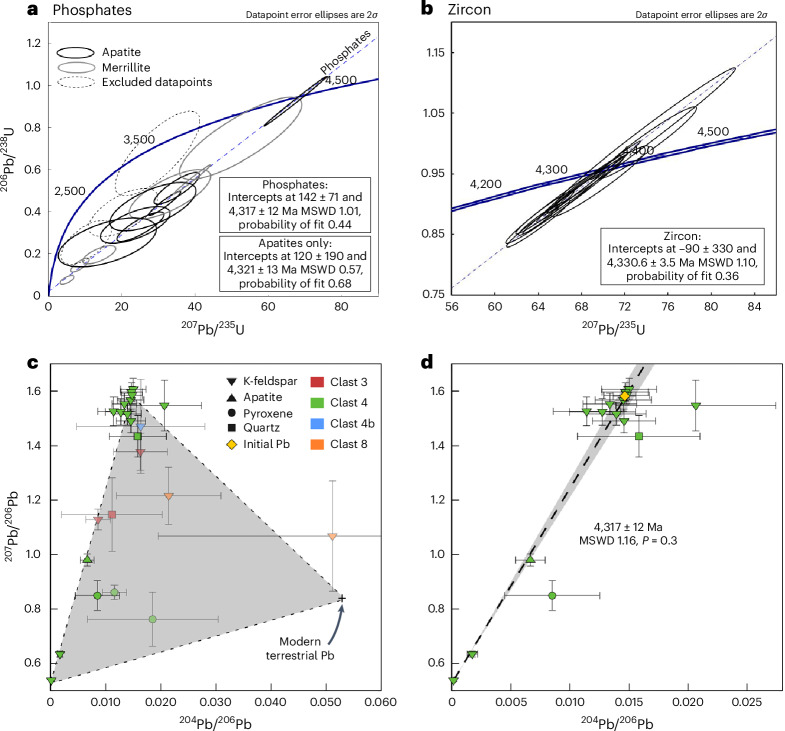
U–Pb and Pb isotope dates of mineral and clast components in the NWA 2995 meteorite. **a**, U–Pb dates of Ca-phosphates in NWA 2995 analysed in Beijing. The discordia (dashed blue line) is fitted to all the data, excluding three datapoints (dashed outlines: Methods), which suffered some Pb loss and do not fall on this isochron (Supplementary Table 10). **b**, U–Pb dates of zircon in NWA 2995 analysed in Stockholm where the dashed blue line is the discordia fitted to the data (Supplementary Table 12). **c**, Pb isotope compositions of phases within four granophyre clasts (Supplementary Table 13). Datapoints included in the final isochron are shown as opaque symbols; those filtered out of the final isochron are semi-transparent. The grey triangle illustrates an assumed mixing relationship between terrestrial Pb contamination^66^ and two end-member lunar Pb components: (1) initial Pb incorporated at the time of clast formation, represented by the highest ^207^Pb/^206^Pb and ^204^Pb/^206^Pb ratios, and (2) radiogenic Pb from the in situ decay of U since that time). **d**, An isochron generated from 15 analyses in clast 4, and the initial Pb composition calculated from the least radiogenic compositions at the top of the isochron. All uncertainty ellipses and bars are shown at 2*σ*; all date uncertainties correspond to 95% confidence intervals.

**Fig. 3:**
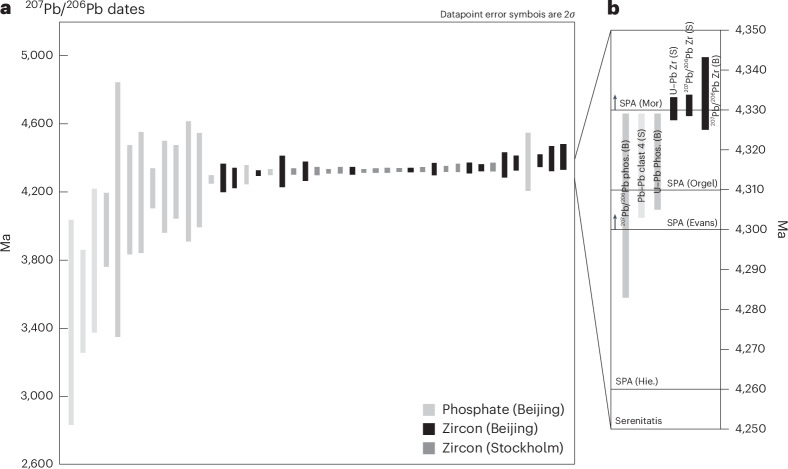
Summary of dates measured in mineral and clast components in the NWA 2995 meteorite. **a**, All individual ^207^Pb/^206^Pb dates ± 2*σ* uncertainties recorded in phosphates (analysed in Beijing) and in zircon grains (analysed in Beijing (B) and Stockholm (S)). The three lighter-grey phosphate dates (left-most side of the plot) represent the datapoints not included in the discordia in Fig. 2a. **b**, A summary of weighted mean ± 95% confidence uncertainty ^207^Pb/^206^Pb phosphate (Phos.) and zircon (Zr.) dates, U–Pb discordia upper intercept dates ± 95% confidence uncertainties (Fig. 2) and Pb–Pb isochron dates ± 95% confidence uncertainty from granophyric clast 4 (Fig. 2d). All dates are reported in the main text and Supplementary Tables 10–13. The dates are compared with the postulated formation ages of the Serenitatis and SPA basins: Hie, Hiesinger et al.^42^; Evans, Evans et al.^44^; Orgel, Orgel et al.^45^; Mor, Morbidelli et al.^5^ (see text for details).

We used secondary ion mass spectrometry (SIMS) to date Ca-phosphates and zircon using the U–Pb and Pb/Pb systems, and silicate mineral phases in granophyre clasts using Pb/Pb (Figs. 2 and 3, Supplementary Figs. 12–14 and Supplementary Datasets 10–13). Some Ca-phosphate and zircon grains are found as isolated fragments within the meteorite matrix, although a few of these grains have one crystal face that is intergrown with exsolved pyroxene (Supplementary Figs. 10, 12 and 13), strongly suggesting that most, if not all, datable mineral phases originated from the QMG lithology.

Zircon grains display a range of textures in backscattered electron (BSE) and cathodoluminescence (CL) images (Supplementary Figs. 10 and 13). Some of the CL-active zones display truncated oscillatory and/or sector zoning, whereas other grains and grain domains are CL inactive. These CL-inactive zones have variable Raman spectra (Supplementary Fig. 9 and Supplementary Dataset 14) and yield weak to no diffraction in electron backscatter diffraction (EBSD) (Supplementary Fig. 11), indicative of a range of crystallinities from relatively pristine to amorphous crystal structure breakdown from radiation damage. EBSD analyses of two zircon grains reveal high levels of strain, with tens of degrees of cumulative misorientation (Supplementary Fig. 11). Zircon #21 also contains <3 μm low-strain domains that are randomly oriented and separated by high angle ‘grain’ boundaries, providing evidence for recrystallization and neoblast growth (Supplementary Fig. 11). No evidence of the high-pressure ZrSiO_4_ polymorph reidite (>30 GPa (refs. ^29,30^)) was identified from Raman spectroscopy or EBSD analyses.

Uranium (24–378 µg g^−1^), Th (7–225 µg g^−1^) and Pb (38–616 µg g^−1^) abundances are variable in the zircon grains (Supplementary Dataset 12) but are within the range of those of Apollo zircons of similar age^31–33^. The U–Pb isotope ratios obtained across all the zircon grains, in both CL-active and CL-inactive areas, yield an upper intercept date of 4,330.6 ± 3.5 Ma (Stockholm: *n* = 12 on 5 different grains for analyses with concordance between 90% and 110%, 95% confidence, mean square weighted deviation (MSWD) 1.10, probability of fit 0.36; Fig. 2b). The corresponding ^207^Pb/^206^Pb dates yielded a weighted average date of 4,331 ± 3 Ma (Stockholm: *n* = 12 on 5 different grains, 95% confidence, MSWD 1.07, probability of fit 0.38; Supplementary Dataset 12). Only Pb–Pb data were obtained in Beijing for zircon, which yielded a weighted average ^207^Pb/^206^Pb date of 4,334 ± 9 Ma (Beijing: *n* = 15 on 9 different grains, 95% confidence, MSWD 3.2, probability of fit 5.7 × 10^−5^; Supplementary Dataset 11) across both CL-active and CL-inactive areas, identical to the Stockholm zircon date. U–Pb ratios in the Ca-phosphates apatite and merrillite, regardless of whether they are isolated in the matrix or found within QMG and/or granophyre lithic clasts, are mostly discordant. Data from 11 out of 14 phosphate grains define a discordia with an upper intercept date of 4,317 ± 12 Ma and a lower intercept at 142 ± 71 Ma (*n* = 13 on 11 grains, 95% confidence, MSWD 1.01; Fig. 2a and Supplementary Dataset 10). Minerals (K-feldspar, pyroxene, silica and apatite) in granophyric clast 4 define a Pb/Pb isochron date 4,317 ± 12 Ma (95% confidence, MSWD 1.16, based on 15 analyses out of a total of 17; Fig. 2c,d and Supplementary Dataset 13; two pyroxene analyses, shown as semi-transparent symbols in Fig. 2c, were filtered out due to Pb isotope compositions indicating terrestrial contamination). Three datapoints from granophyric clast 3 and one from clast 4b also appear to be consistent with an equivalent isochron date and initial Pb isotope composition (Fig. 2c,d). In addition, two K-feldspar analyses from granophyric clast 8 would be consistent with the same isochron but show signs of terrestrial contamination (Fig. 2c,d).

### SPA origin for the NWA 2995 meteorite

NWA 2995 has a reported bulk rock FeO abundance of 9.75 wt% (ref. ^20^), which is intermediate between feldspathic (3–7 wt% FeO) and basaltic (17–23 wt% FeO) lunar igneous rocks, similar to some mixed-lithology Apollo mafic impact melt breccias. The meteorite has a bulk Th abundance of 1.55 µg g^−1^ (ref. ^20^), intermediate between KREEP-rich and KREEP-poor (where KREEP is defined as potassium, rare earth elements and phosphorous) lunar rocks and soils indicating that it was sourced from a moderately mafic region of non-mare crust. Korotev et al.^20^ and Mercer et al.^21^ have argued that the meteorite’s unusual bulk composition (that is, ferroan mafic and high bulk rock Th/Sm ratios^20^) means that, rather than being affiliated with a nearside Procellarum KREEP origin, it is compositionally similar to regolith in the farside SPA basin. This is consistent with the lack of an Imbrium basin age (ca. 3.92 Ga) overprint in any of the isotopic systems in NWA 2995.

**Fig. 4:**
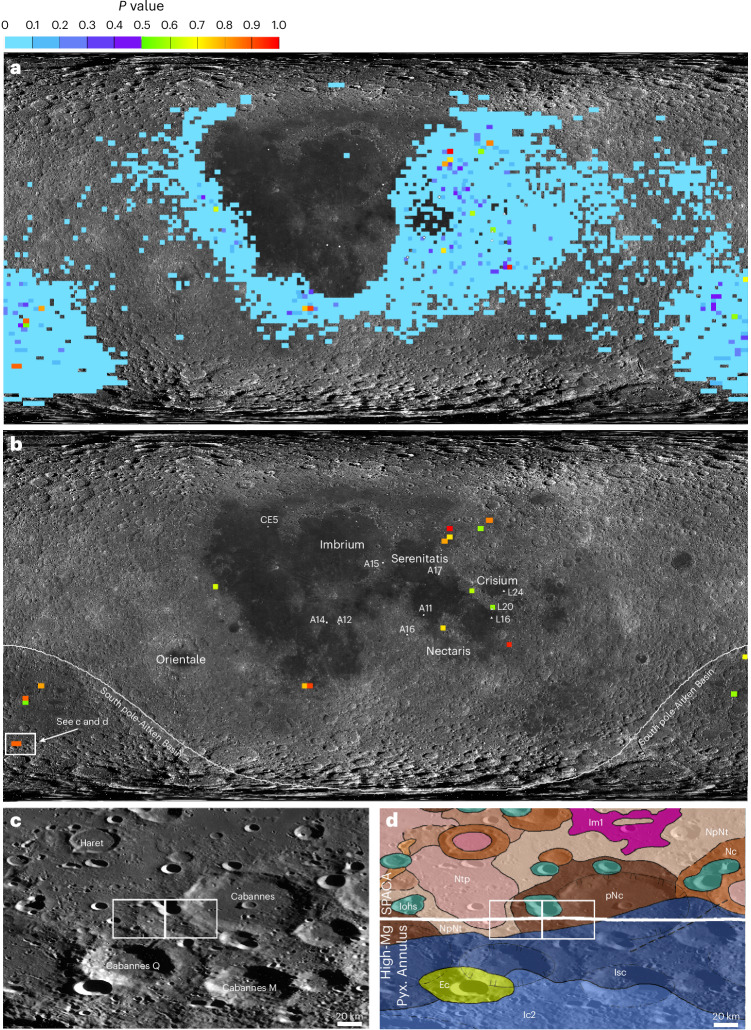
Assessment of lunar provenance of the NWA 2995 meteorite. **a**,**b**, Maps of probability *P*-index values >0 (Supplementary Table 15) (**a**) and of probability *P*-index values >0.5 (Supplementary Table 16) (**b**) where the Lunar Prospector gamma-ray^34^ dataset (rebinned to be 0.5° per pixel) has a similar chemical composition equivalent to the NWA 2995 bulk rock composition (see Supplementary Note 1 for details). The data are overlaid on an equidistant cylindrical projection LRO WAC albedo map of the Moon. Apollo (A), Luna (L) and Chang’e–5 (CE5) landing sites are denoted along with the locations of the basins mentioned in the text. **c**,**d**, Close-up images of the Cabannes crater ROI pixel boundaries (white rectangles) superimposed on an equidistant cylindrical projection LRO WAC basemap (**c**), and on top of the USGS Unified Geologic Map of the Moon at 1:5,000,000 (**d**). In stratigraphic order from youngest to oldest: Ec, Erathanian aged Crater Unit; Iohs, Imbrian aged Orientale Hevelius Formation, Secondary Crater Facies Unit; Ic2, Imbrian aged Upper Crater Unit; Im1, Imbrian aged Lower Mare Uni; lsc, Imbrian aged Secondary Crater Unit; Nc, Crater Material Younger Than Nectaris Basin, but Older Than Imbrium Basin; NpNt, Nectarian aged Terra Unit; Ntp, Nectarian aged Plains and Mantling, Terra Unit; pNc, Pre-Nectarian Crater Unit. The horizontal white line in **d** is the boundary between the SPACA and the Mg-rich pyroxene (Pyx) southern SPA annulus region^14^.

We have investigated this hypothesis through a chi-squared analysis that compares the meteorite’s bulk rock composition (including SiO_2_, Al_2_O_3_, MgO, FeO, TiO_2_, CaO, Th, K and U abundances) and the Lunar Prospector gamma ray lunar surface composition dataset^34^ (see Supplementary Note 1 for the approach). This enables us to identify regions of the Moon’s surface regolith that are most chemically similar to the NWA 2995 meteorite, corresponding to the most likely launch sites. The probability map of likely launch sites is shown in Fig. 4, where each pixel represents 15 × 15 km. This analysis shows that the meteorite was probably not sourced within the nearside Procellarum KREEP Terrane surrounding the Imbrium basin. Pixels with higher *P* values denoting most similar compositions are within the central SPA basin on the lunar farside, and some areas in the eastern nearside limb mare–highland areas surrounding the Crisium and Nectaris impact basins, and the Fecunditatis and Tranquillitatis mare basins. Supplementary Dataset 21 outlines the most statistically probably regions of interest (ROIs) where *P* ≥ 0.5, with details of the ROI’s geological context and the age of the geological unit deduced from crater counting techniques and/or stratigraphic relationships. Of these localities, only two ROIs have mapped Pre-Nectarian surface ages in range of the 4.32–4.33 Ga radiometric dates determined for evolved lithologies in NWA 2995; all the other ROIs have surface ages younger than 4.04 Ga (Supplementary Dataset 21). Both of the Pre-Nectarian ROIs are adjacent to each other within the SPA basin, specifically to the southwest of the 81-km-diameter Pre-Nectarian Cabannes crater (60.9° S 169.6° W) (Fig. 4c,d). The Cabannes crater is located on the southern boundary of the central SPA compositional anomaly (SPACA: dominated by non-mare Fe-rich augite- and pigeonite-bearing lithologies) and the non-mare Mg-richer pyroxene southern SPA annulus region^14^. It is also within the central depression zone of SPA, thought to represent the basin’s differentiated impact melt pile. Within these two ROIs, Lunar Reconnaissance Orbiter narrow-angle camera images reveal there are multiple small fresh impact craters on the scale of hundreds of metres to a few kilometres, which are in the typical size range of craters from which lunar meteorites are thought to be launched from^35^.

## Discussion

The oldest 4.33 Ga date recorded in NWA 2995 zircon could represent either a minimum age for crystallization of the QMG lithologies or a major isotopic resetting event when the QMGs were excavated and deposited in the near lunar surface environment. As the U–Pb system in zircon is more resistant to thermal isotopic disturbance than in Ca-phosphates^36,37^, zircon typically yields older ^238^U/^206^Pb and ^207^Pb/^206^Pb dates than Ca-phosphates in a given rock. Notable in lunar samples (including Mg-suite rocks and various impact melt breccias), Ca-phosphates often yield a date of ~3.92 Ga interpreted to be thermal overprinting by the formation of the Imbrium basin^33,38,39^. In NWA 2995, we see no evidence of a 3.92 Ga overprinting event (Fig. 3). A few of the phosphates record Pb isotope disturbance dates between 3.4 and 4 Ga (Supplementary Dataset 10); however, the much more recent 142 ± 71 Ma date recorded by the U–Pb resetting of phosphate grains (Fig. 2a) could be interpreted as the date NWA 2995 was assembled at the lunar surface when it was consolidated from a soil into a regolith breccia. The coincident zircon, most Ca-phosphates and major rock-forming mineral ^207^Pb/^206^Pb dates all point to a consistent major event at 4.32–4.33 Ga (Fig. 3), suggesting that this date corresponds either to crystallization and fast closure of the U–Pb and Pb/Pb radiometric systems in these different phases followed by no further disturbance, or to disturbance and resetting of all these systems by a single geological event. Evidence for intensive plastic deformation and recrystallization in some zircon grains (Supplementary Fig. 11) favours the latter hypothesis. Therefore, we interpret this 4.32–4.33 Ga date to represent the timing of a major lunar event that reset all the isotopic chronometers in NWA 2995.

The absolute ages and relative stratigraphic order of the Moon’s impact basins is debated, with recent studies arguing for the following order: Orientale < Imbrium (3.92 Ga (ref. ^38^)) < Crisium < Nectaris < Serenitatis (4.1 Ga (ref. ^5^) to 4.2 Ga (ref. ^40^) to 4.25 Ga (ref. ^41^)) < SPA. Crater size-distribution model ages for the SPA basin range from ~4.26 Ga (refs. ^42,43^) to >4.3 Ga (ref. ^44^) to 4.31 Ga (ref. ^45^) to >4.33 Ga (deduced from the oldest regions of the basin floor^5^). The preserved structure of the SPA basin implies that it must have formed after the lunar crust had formed and cooled by ~4.35 Ga (ref. ^46^). We note that older impact basins may have existed before this time, but their surface expression has not been preserved.

The U–Pb and Pb/Pb radiometric dates recorded by NWA 2995 (4.32–4.33 Ga) are, thus, older than those proposed for the Serenitatis basin-forming event (~4.1–4.25 Ga (refs. ^5,40,41^)) and by this logic are also older than the Nectaris or Crisium basins. They are clearly not associated with resetting by the much younger Imbrium event at 3.92 Ga (ref. ^38^). Overall, NWA 2995 records U–Pb and Pb/Pb dates that are within the age range proposed for formation of the SPA basin (ca. 4.26 to >4.33 Ga; Fig. 3) and is compositionally similar to Pre-Nectarian regolith surfaces within the basin floor (Fig. 4). Therefore, we propose that the meteorite originated from the SPA basin and that its 4.32–4.33 Ga U–Pb and Pb/Pb radiometric dates represent the basin-forming event.

The QMG clasts in NWA 2995 are highly evolved magmatic rocks that were probably formed from magmas that underwent silicate liquid immiscibility. Equivalent exsolved pyroxene-bearing Apollo high-Alkali Suite silicic samples are estimated to have formed at relatively shallow depths of 200 m (Apollo 15 15403 QMD^26^) to 0.7–2 km (Apollo 14 14161,7373 monzogabbro^27^). These QMG lithologies sampled by NWA 2995 could be an important component of the upper pyroxene-rich unit of the SPA impact melt pond^11,12,47^. If so, QMG is probably the main contributor of the small Th-bearing KREEP signature seen within the SPA basin floor central zone^14,34^.

A 4.33–4.32 Ga age for SPA—at the older end of previous crater-counting estimates (Fig. 3b)—has important implications for the early bombardment history of the Moon. This supports the suggestion that SPA was a unique event in the inner Solar System history, which pre-dated the main lunar basin-forming period between approximately 4.2 Ga and 3.8 Ga by ~120 million years. This suggests either an accretion tail-end^5^ or saw-tooth^6^ lunar impact model, rather than the terminal cataclysm model where all lunar basins are proposed to have formed in a narrow window at ~3.9 Ga (ref. ^48^). As SPA is stratigraphically the oldest basin on the Moon, a 4.33–4.32 Ga age also implies that older impact basins with no surface expression must have been erased. This most likely was from global resurfacing caused by the SPA basin formation itself^15^, or alternatively from other processes such as the early lunar crust having a high heat flow causing crustal relaxation^49^.

Our proposed formation age for SPA will require confirmation from future radiometric dating of samples collected from the south of the Apollo basin area by the Chang’e–6 mission^50^, or from future proposed missions such as the Endurance-A rover concept that aims to collect 100 kg of samples from across the SPA basin floor^3^. Such mission designs will require (1) careful selection of sampling localities to maximize the likelihood of collecting material from basin-formed impact melt and/or differentiated impact melt products^7^, and (2) precise geological mapping and crater counting of the landing site/sampling site areas to enable calibration of the lunar cratering chronology back to 4.3 Ga, helping to determine the surface ages of the oldest regions of the lunar highlands^1,2,6^.

## Methods

The section of NWA 2995 we studied was provided from the type specimen sample curated in the former Northern Arizona University collection (Ted Bunch).

### Scanning electron microscopy

A 1.0 × 0.7-cm-thick section of NWA 2995 was polished, carbon coated and analysed using scanning electron microscopy (SEM) at the University of Manchester to derive whole-sample qualitative energy-dispersive spectroscopy X-ray element distribution maps and a BSE image (Fig. 1 and Supplementary Fig. 1).

Images were collected using different instruments including a FEI XL30 environmental scanning electron microscope–field emission gun (FEG) with EDAX Genesis software for energy-dispersive spectroscopy, a quanta 650 FEG environmental scanning electron microscope with Brucker software, a Zeiss Merlin FEG-SEM with Atzec software, and a JEOL-Electron Probe Microanalyzer (EPMA) 8530F. BSE and element maps were collected with an accelerating voltage of 20 kV. False colour element maps were produced using ImageJ to assess the diversity of clasts within each section (see approach of Joy et al.^51^). Each element (Si, Al, Ca, K, Fe, Mg and Ti) was assigned a colour designed to highlight material from various geological provenances (for example, highland anorthosite, mare basalts and granitic material). The images were then finalized in Adobe Photoshop, where some image processing (contrast and brightness stretch) highlighted material further (that is, highlighting clast boundaries) and removed ‘noise’ around the sample (Fig. 1). Mineral phases and clasts of interest were imaged at high spatial resolution using BSE using the same instruments as described above (Supplementary Figs. 1–4).

The chemical compositions of silicate mineral phases, glass and apatite were analysed at the Institute of Tibetan Plateau Research, Chinese Academy of Sciences with a JEOL JXA-8230 EPMA. All reported data were checked to be above the instrument detection limits (<0.05 wt%). Typical uncertainties were ~0.1 wt% and ~0.05 wt% for major and minor elements, respectively. All data were also checked for acceptable analytical totals of between 98% and 102%, and mineral compositions were checked for stoichiometry. Data are reported in Supplementary Datasets 1–6 and shown in Supplementary Figs. 5–7.

The Ti abundance in SiO_2_ phases was determined using a Cameca SX100 EPMA at the University of Manchester. Analyses were carried out using a 25 kV accelerating voltage, a 60 nA beam current and a defocused 5 μm beam size. Measurements were made using LPET and PET spectrometers. Rutile was used to identify the position of the TiO_2_ peak and backgrounds positions. Spectrosil glass (pure SiO_2_) was used a Ti-free reference material. Counting times for each spectrometer was 480 s on peak and 480 s off peak. Under these beam conditions, detection limits of 2.4 µg g^−1^ Ti were achieved. Data are reported in Supplementary Dataset 7.

### CL imaging

Optical microscope-CL imaging of the whole sample (Fig. 1) was acquired at the University of Manchester using a CITL 8200 mk3 ‘cold’ CL system coupled to a transmitted-light microscope; images were collected at ~15 kV and a current of ~300 mA.

CL imaging of the zircon grains (Supplementary Figs. 10 and 13) was taken using the JEOL-EPMA 8530F at the University of Manchester using 10 kV accelerating voltage and 20 nA current.

### EBSD

EBSD analysis of two zircons in NWA 2995 (#21 and #44; Supplementary Fig. 11) was performed at the Electron Microscopy and Micronalysis Unit of the University of Portsmouth, using a Zeiss Evo10MA LaB6 SEM equipped with an Oxford Instruments nano EBSD detector and HKL Channel 5 software. Before EBSD analysis, the sample surface was repolished for 40 min using a 0.05 μm alumina suspension to remove the surface defects caused by specimen preparation. EBSD data were acquired using an accelerating voltage of 20 kV, a beam current of 2 nA, a step size of 100–300 nm and a specimen tilt angle of 70°. The phase and orientations of individual EBSD patterns were indexed using the unit cell data of Hazen and Finger^52^ for zircon, and Farnan et al.^53^ for reidite (although no subgrain domains were indexed as reidite).

### SIMS

#### Ca-phosphate U–Pb dating (Beijing)

In situ isotopic U–Pb dating of Ca-phosphates was performed on a large geometry multi-collector CAMECA IMS-1280 ion microprobe at the Institute of Geology and Geophysics, Chinese Academy of Sciences in Beijing. Samples were carbon coated before SIMS analysis. U–Pb dating for P-bearing minerals, apatite and merrillite in NWA 2995 was conducted in mono-collection mode (see Li et al.^54^ for technical details). The primary O^−^ beam was tuned to ~15 × 10 μm in size at 14 nA in Gaussian mode. During the acquisition, a 5 μm raster was performed to avoid creating deep craters. The species ^204^Pb^+^, ^206^Pb^+^, ^207^Pb^+^, ^208^Pb^+^, ^232^Th^+^, ^238^U^+^, ^232^Th^16^O^+^, ^238^U^16^O^+^ and ^238^U^16^O_2_^+^ were measured sequentially for 6, 6, 12, 1.04, 1.04, 6, 1.04, 1.04 and 4 s using an axial electron multiplier in peak jumping mode. The mass resolving power was set at 8,450 (defined as 50% peak height) by combining a field aperture of 5,000 μm, an entrance slit of 60 μm, an energy slit of 60 eV and a field magnification of 200×. Each measurement for U–Pb dating consisted of 12 cycles and lasted ~19 min. Repeated analyses of NW-1 apatite (U–Pb age of 1,163.5 ± 3.5 Ma (ref. ^55^)) was used as a standard to calibrate the U–Pb fractionation during the analysis and yielded ^206^Pb/^238^U = 0.192 × (^238^U^16^O_2_/^238^U)^0.802^ (*n* = 12, *R*^2^ = 0.93). All Ca-phosphate U–Pb data are presented in Supplementary Dataset 10. Data displayed in the conventional Concordia diagram (Fig. 2a) have been corrected for common Pb contribution, assuming that all common Pb was terrestrial Pb contamination with a ^207^Pb/^206^Pb of 0.836.

The location of SIMS analyses and BSE images of the dated Ca-phosphate phases are given in Supplementary Fig. 12.

#### Zircon Pb–Pb dating (Beijing)

Pb–Pb isotopic dating was performed on zircon in NWA 2995 with a small beam size of <5 μm under mono-collector mode using the same instrument than for Ca-phosphate U–Pb dating. The detailed analytical method can be found in Liu et al.^56^ and references therein. In brief, we used a 0.4 nA O^−^ primary beam tuned to a diameter of ~4.5 μm. For each analysis, ^180^Hf^16^O^+^, ^94^Zr_2_^16^O^+^, ^204^Pb^+^, ^206^Pb^+^ and ^207^Pb^+^ were measured sequentially using an axial electron multiplier in peak jumping mode for 0.48, 0.32, 8, 4 and 10 s, respectively. Each analysis included ten cycles and lasted ~10 min. Oxygen was flooded into the sample chamber (~1 × 10^−6^ torr pressure) to enhance the ion yield of Pb^+^. A mass resolving power of 8,000 was achieved by using a field aperture of 6,000 μm, an entrance slit of 60 μm, an energy slit of 50 eV and a field magnification of 200×. We used the ^180^Hf^16^O signal in imaging mode to locate the zircon grains and accurately position the beam for analysis. The accuracy of ^207^Pb/^206^Pb analyses was monitored by repeated analyses of the Phalaborwa baddeleyite, which yielded a weighted mean ^207^Pb/^206^Pb date of 2,059 ± 6 Ma (2*σ*, *n* = 5, MSWD 0.19), identical to its known date of 2,060 ± 1 Ma (ref. ^57^). Measured ^207^Pb/^206^Pb ratios have been corrected for common Pb contribution assuming that all common Pb was terrestrial Pb contamination with a ^207^Pb/^206^Pb of 0.836. Data are presented in Supplementary Dataset 11. Note that using a lunar meteorite like ^207^Pb/^206^Pb initial ratio of 1.6 (ref. ^58^) instead makes no difference (^207^Pb/^206^Pb-weighted average date of 4,333.0 ± 9.4 Ma (MSWD 3.4) versus 4334.1 ± 9.1 Ma (MSWD 3.2) for a common ^207^Pb/^206^Pb ratio of 0.8356 for the 15 analyses).

The location of SIMS analyses and BSE and CL images of the zircon phases are given in Supplementary Fig. 13.

#### Zircon U–Pb dating and clast Pb–Pb dating (Stockholm)

Additional analyses of the U–Th–Pb systematics of zircon in NWA 2995, as well as analyses of the Pb isotopic compositions for phases in the granophyre clasts, were performed using methodologies similar to those outlined in previous studies (for example, refs. ^59–63^) using the CAMECA IMS-1280 ion microprobe at the NordSIMS facility, Swedish Museum of Natural History, Stockholm. Several parameters were common to both types of analysis. An Oregon Physics Hyperion H201 radiofrequency plasma source, operated in critical (Gaussian) focusing mode, was used to generate an oxygen (O_2_^−^) primary beam. A 5 nA current was used for all of the U–Th–Pb zircon analyses and the Pb isotope analyses of small targets in the NWA 2995 granophyre clasts, resulting in a spot size of ca. 10 µm, including a 5 µm raster to flatten the crater bottom. At the start of each analysis a 70 s pre-sputter over a 20 × 20 µm area was used to remove the gold coating and/or surface contamination. The instrument was operated in high-transmission mode, corresponding to a transfer magnification of 160×. In this mode, the field aperture size of 1,000 µm (Pb isotopes) or 1,200 µm (zircon) was chosen to limit the field of view on the sample surface (that is the area from which ions will be admitted to the mass spectrometer) to a 6 × 6 µm or 7.5 × 7.5 µm square, respectively, larger than the unrastered spot, but smaller than the pre-sputtered area, further minimizing the possibility of measuring surface contamination. Analyses of the reference materials (basaltic glass for Pb isotopes, zircon for U–Pb) utilized a larger primary beam (20 nA) and field aperture (3,000–4,000 µm).

For the Pb isotope measurements, a nuclear magnetic resonance field sensor regulated the stability of the magnetic field to high precision. The Pb isotopes were measured simultaneously in multi-collector mode using four electron multipliers. The mass spectrometer was operated with a mass resolution of 4,830 (*M*/Δ*M*), sufficient to resolve Pb from known molecular interferences. Each analysis comprised 60 integrations (20 for reference materials) of 20 s per cycle. In addition to the sample analyses, measurements were also made of the United States Geological Survey (USGS) basaltic glass reference material, BCR-2G. By comparing with the BCR-2G Pb isotope ratios of Woodhead and Hergt^64^, these measurements were used to generate a correction factor to compensate for mass fractionation (a few parts per thousand at Pb mass^65^) and detector relative gain (a few percent) in the unknown analyses. Specifically, the ‘accepted’ isotope ratios for BCR2-G (determined independently using TIMS analyses^65^) were divided by the corresponding average of each ratio obtained from all the BCR2-G analyses in the session. Isotope ratios of unknown samples were then corrected by multiplying by these factors. No systematic drift was observed in the BCR2-G measurements during the analytical session, as demonstrated by the 1*σ* standard deviations from the average session value all being less than 1% (the complete set of average session values and associated standard deviations are reported in Supplementary Dataset 13). The overall uncertainties stated for each ratio in the individual sample measurements (Supplementary Dataset 13) incorporate the internal run error propagated together with the standard deviations of the BCR-2G analyses for the relevant analytical session and the uncertainty given for the published BCR-2G values^65^. Background counts for each channel were measured for 2 s each cycle during each run by blanking the secondary beam using deflectors located before and after the electrostatic sector. The average background values for the session are reported in Supplementary Dataset 13. Individual analyses were filtered out of the final dataset if the count rates for any of the masses were less than 3× the average background count rates during that session. The Pb–Pb isochron dates are quoted with errors stated at the 95% confidence level.

For the zircon U–Pb analyses (Supplementary Dataset 12), a single low-noise (<0.003 counts s^−1^) ion counting electron multiplier (ETP 14133H) with an electronically-gated deadtime of 44 ns was used to measure secondary ion beam intensities in the mass-switching sequence: 196 (^90^Zr_2_^16^O), 204 (Pb), 206 (Pb), 207 (Pb), 208 (Pb), 235 (U), 238 (U), 248 (^232^Th^16^O), 254 (^238^U^16^O) and 270 (^238^U^16^O_2_). The mass spectrometer was operated with a mass resolution of 5,400 (*M*/Δ*M*), sufficient to resolve Pb from known molecular interferences. Non-radiogenic (common) Pb monitored using ^204^Pb was assumed to be the result of terrestrial contamination and was corrected using the Stacey and Kramers^66^ model for present-day terrestrial Pb isotopic compositions. Sample Pb/U ratios were calibrated against the 561 Ma M257 zircon standard (840 µg g^−1^ U, ^206^Pb/^238^U = 0.090975 (ref. ^67^)) using a power law relationship between measured ^206^Pb/^238^U and ^238^U^16^O/^238^U ratios.

Data reduction was performed using in-house developed software at NordSIMS and the Excel add-in Isoplot (v. 4.15^68^).

The location of SIMS analyses and BSE images of the other mineral phases (K-feldspar, pyroxene, quartz and apatite) targeted for Pb/Pb dating are given in Supplementary Fig. 14. The zircon U–Pb data can be found in Supplementary Dataset 12, and Pb isotope data are in Supplementary Dataset 13.

### Raman spectroscopy

Micro-Raman spectroscopy was carried out using a Horiba Xplora Plus instrument at the University of Manchester using a green laser light with a wavelength of 532 nm. Spectra were background corrected and calibrated against a polystyrene reference material as described in ASTM E1840-96^69^. Data were collected using the LabSpec 6 software.

Two sessions were used to collect point spectra on zircon grains using a 1,200 gr mm^−1^ grating, 100× magnification objective and 5–10 s acquisition time

Data for quartz were collected across two sessions. Point spectra were collected with a 1,200 gr mm^−1^ grating, using a 50× objective and 5–5.5 s acquisition time. For both minerals, the 1,200 gr mm^−1^ provides a spectral resolution of ~2.8 cm^−1^ per pixel.

We quote Raman peak positions with ±1.5 cm^−1^ calibration errors as in Itoh et al.^70^, which includes any error of <1 cm^−1^ temporal drift in spectrometer calibration possible between calibrations^71^. Raman data presented are background reduced via application of an iterative method^72^.

#### Analysis of Si phases

There is no apparent evidence for a phase transition for quartz, which is characterized in Raman spectra by a (Si–O–Si) stretching mode resulting in a peak at ~464 cm^−1^. Any transition from quartz into higher-pressure phases would alter the structure such that the (Si–O–Si) stretching mode is shifted to higher wavenumbers: 521 cm^−1^ for coesite (which splits into a doublet at pressures >25 GPa) and 750 cm^−1^ for stishovite^73^. However, there is evidence for extensive amorphization of the structure, resulting in much broader spectral peaks with similar quartz peak position (Supplementary Fig. 8). Peaks within the 400–600 cm^−1^ mode for vibration of tetrahedral tectosilicates are evident^74^. Note, however, that silicate glasses share the same peak position with crystalline polymorphs^74^.

An overprint of feldspar on K-feldspar quartz is indicated by the appearance of a characteristic Va peak for feldspars at 513–515 cm^−1^, which correlates to orthoclase. However, overlap of the ~482 cm^−1^ Vb peak with the quartz 464 cm^−1^ peak obscures further classification including intermediate compositions^75,76^. The feldspar Va peak at 513–515 cm^−1^ can be clearly resolved from the 521 cm^−1^ characteristic peak for coesite^77^.

#### Analysis of zircon

The spectra of Zircon 41 have dominant peaks at ca. 344, 426 and 1,003 cm^−1^ (Supplementary Fig. 9) resulting from the fundamental vibration modes of zircon SiO_4_ tetrahedra. This grain represents a relatively unmodified (unshocked, no radiation damage) zircon. Several zircon grains (#1, #15, #43, #62, #63 and some portions of #21 and #36) have Raman spectra that are broader and have less obvious bands than unshocked terrestrial zircon (Supplementary Fig. 9), indicating that Si–O–Zr bonds in the ZrSiO_4_ group in these crystal lattices have been damaged to form shorter Si–O bonds^29^. No reidite (shock polymorph of zircon) bands (~559, 851 and 893 cm^−1^; Supplementary Fig. 9) are present; instead, the broadening and rightward shift of the 344 and 426 cm^−1^ peaks and increase of the intensity of a peak at ~810 cm^−1^ are more similar to metamictization from radiation damage effects seen in terrestrial zircon^78^. Three zircon grains (#35, most of #36 and a portion of #21) have much broader smoother Raman spectra, with a broad ‘hump’ centred at ~504 cm^−1^ (Supplementary Fig. 9). These spectra were mostly collected from CL-inactive areas of these zircon, and indicate breakdown of the crystalline structure from radiation damage and show no indication of the presence of reidite.

### Meteorite launch location search

Details of this method and verification are provided in detail in Supplementary Note 1.

## Supplementary information

Supplementary Information Supplementary Figs. 1–14 and Note 1, containing Figs. 15–19 and Datasets 17–21.

Supplementary Data File containing Supplementary Datasets 1–16.

## Data Availability

All data are available in the tables provided in the main text and in Supplementary Information.

## Code availability

The Interactive Data Language (IDL) routine used to analyse the meteorite samples is available from the corresponding author upon reasonable request.
