# Supplementary material for: Evidence of a 4.33 billion year age for the Moon’s South Pole–Aitken basin
Source: Nat Astron. 2024 Oct 16;9(1):55–65. doi: 10.1038/s41550-024-02380-y (PMC11757148; doi:10.1038/s41550-024-02380-y)
Supplement: Supplementary file 1 — Supplementary Figs. 1–14 and Note 1, containing Figs. 15–19 and Datasets 17–21. [file 41550_2024_2380_MOESM1_ESM.pdf]

nature astronomy

Supplementary information https://doi.org/10.1038/s41550-024-02380-y

Evidence of a 4.33 billion year age for the Moon's South Pole–Aitken basin

In the format provided by the authors and unedited

Overview sample context

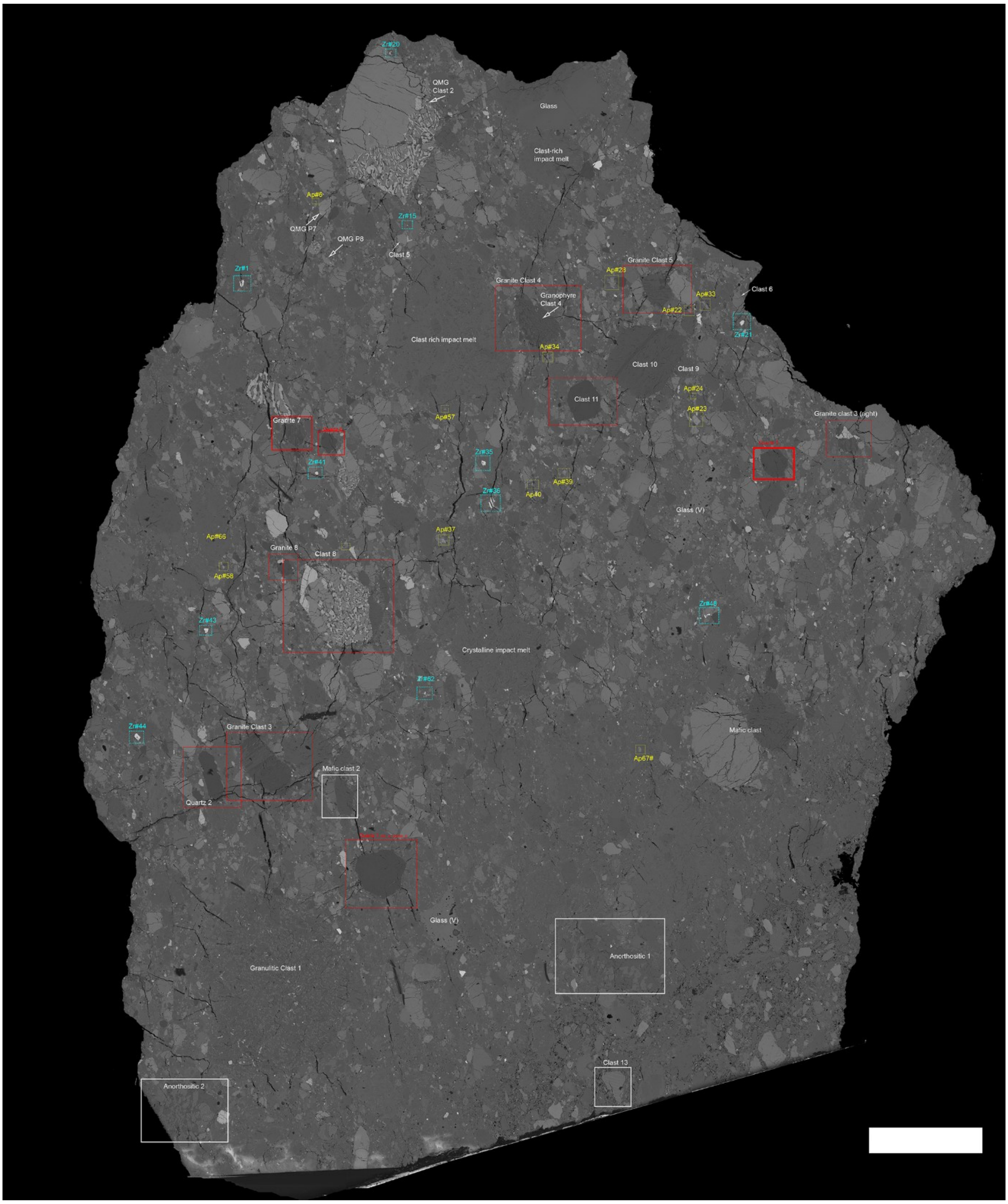

Supplementary Figure 1. Backscattered electron map showing NWA 2295 with assigned clast and phase names that are referred to elsewhere in this study. The red squares indicate the location of evolved clasts (i.e., Si-rich), white squares indicate other key clast types, yellow font/squares correspond to phosphate locations, and cyan coloured font/boxes display the location of are Zr-rich phases.

Example of clast BSE images

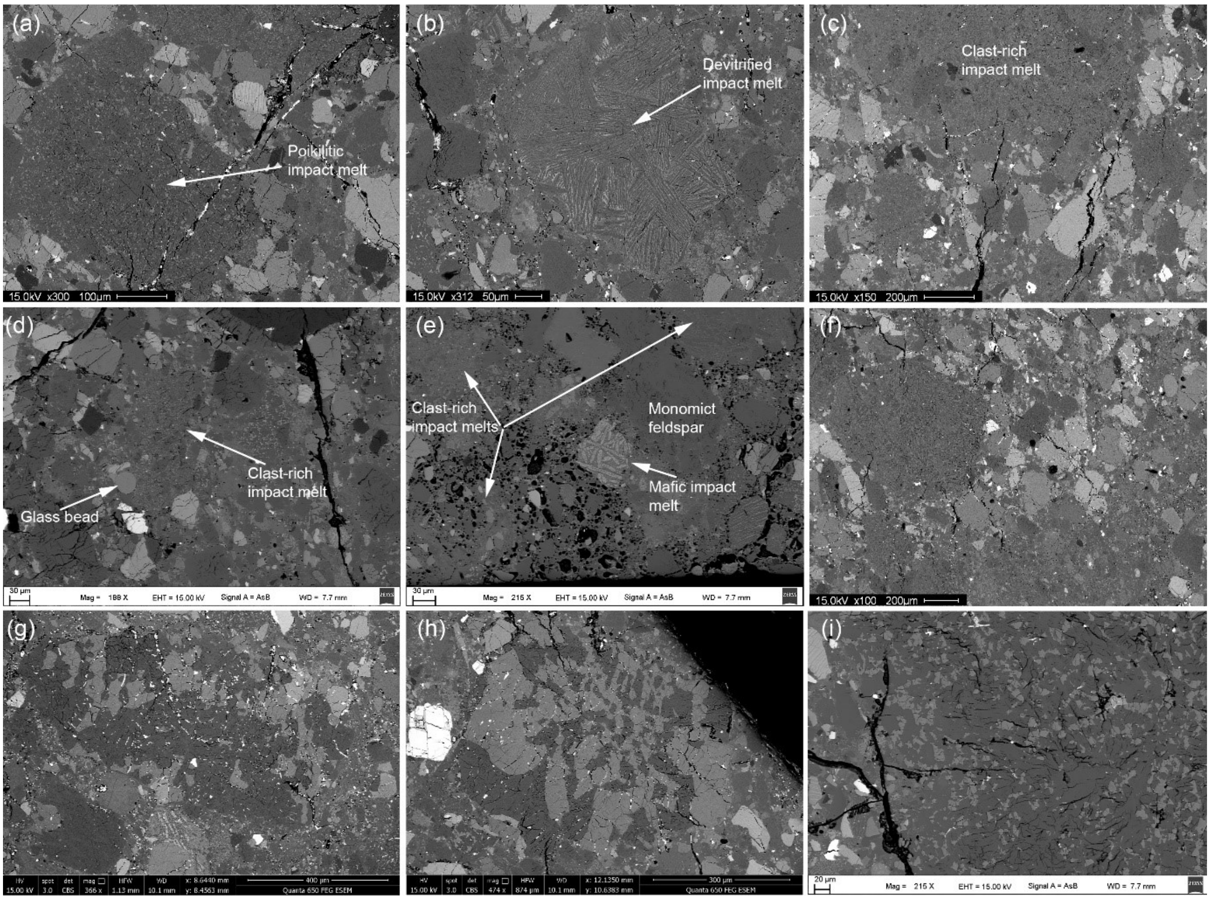

Supplementary Figure 2. BSE images of feldspathic clasts, impact melt clasts, and matrix in NWA 2995. (a) Poikilitic impact melt clast, (b) devitrified impact melt clast, (c) large clast-rich impact melt clast. (d) Matrix area with clast-rich impact melt clast and a small ~30 µm glass spherule. (e) Matrix area including a vesiculated zone, and clasts of clast-rich impact melt, a devitrified mafic impact melt and monomict mineral fragments. (f) Typical matrix region with variable clast sizes. (g) Anorthositic clast 1. (h) Anorthositic clast 2. (i) Granulitic textured clast. Bright phases in cracks is residual material from Au-coating after SIMS work.

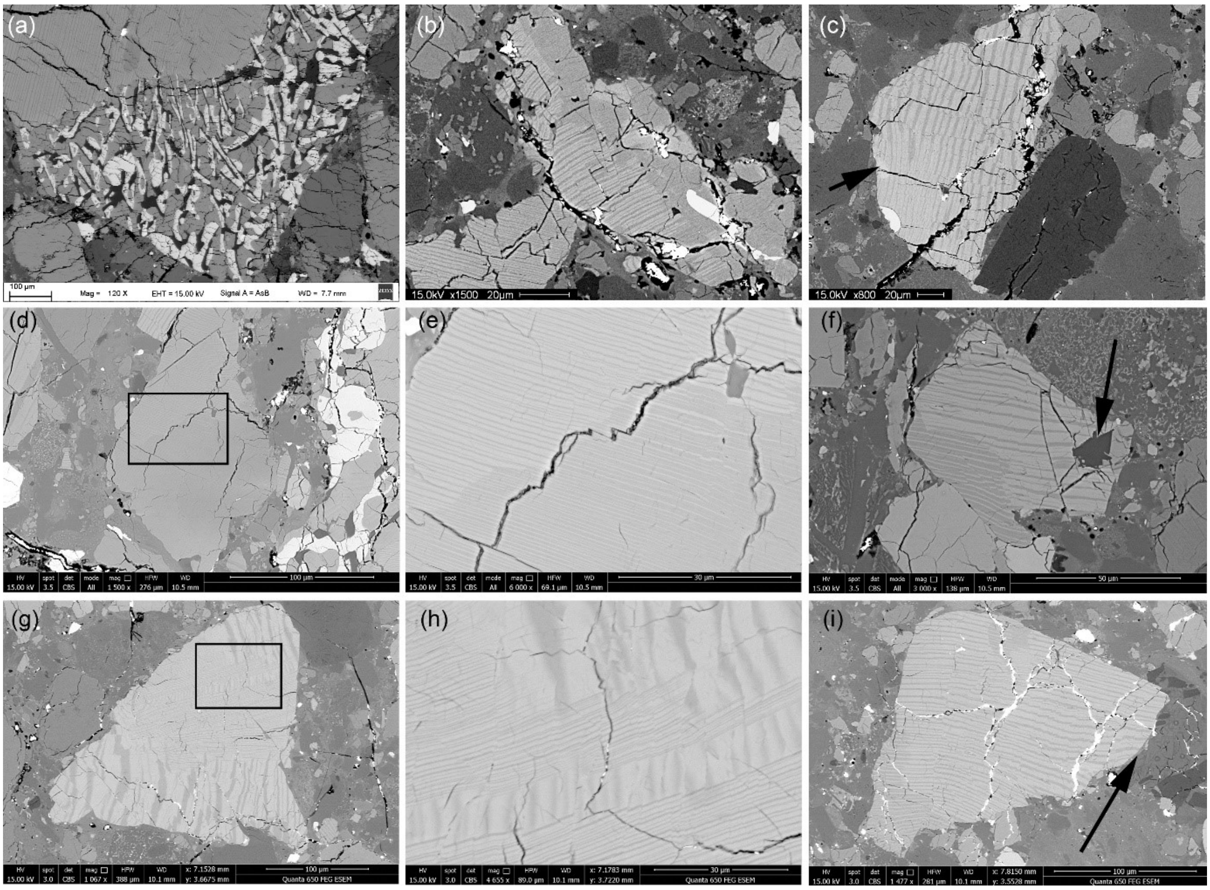

Supplementary Figure 3. BSE images of selected gabbroic clasts. (a) Large QMG Clast 2 with a region of finely exsolved pyroxene and symplectite assemblage with pyroxene, fayalite, and silica. (b) Complex exsolved pyroxene clast. (c) Apatite bearing QMG Clast P7, where the arrow indicates the location of the apatite grain. (d) Finely exsolved pyroxene clast where square inlay is shown in close up (e). (f) Finely exsolved pyroxene clast with Si-rich inclusion (indicated by arrow). (g) Complex exsolved pyroxene clast where square inlay is shown in close up in (h). (i) Pyroxene clast ROI 2 where the exsolved pyroxene shares a grain boundary with a granophyre area (indicated by black arrow).

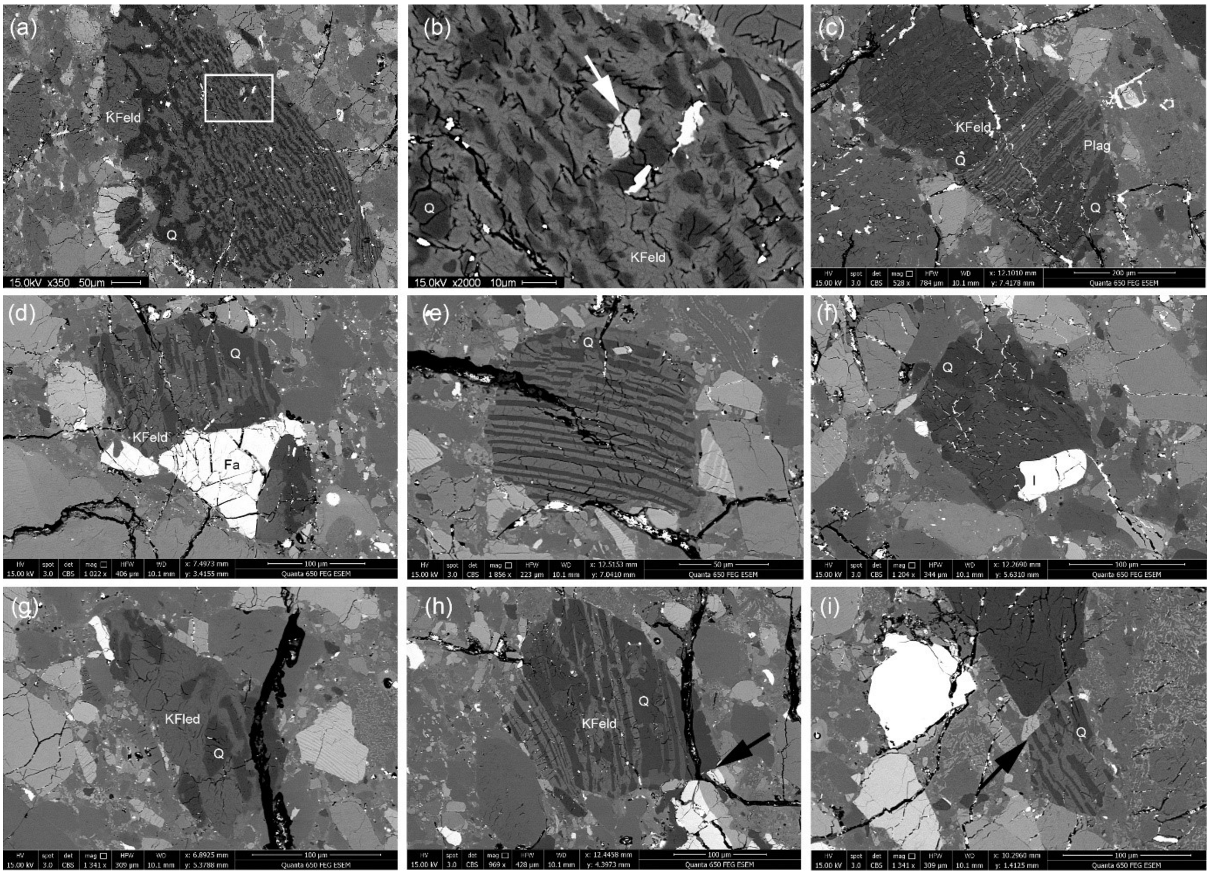

Supplementary Figure 4. (a) Granophyric clast 4 with irregular intergrowth of quartz (Q) with K-feldspar (KFeld) and apatite (indicated by arrow in (b)). (b) Close-up of white square area shown in (a). (c) Granophyric clast 3 composed of platy intergrowth of quartz with K-feldspar and plagioclase (Plag). (d) Granophyric clast 3b with intergrowth of quartz, K-feldspar, and fayalite (bright phase labelled Fa). (e) Granophyric clast 9 with platy intergrowth of quartz (darker phase) with K-feldspar (lighter phase). (f) Granophyric clast 8 with granular intergrowth of quartz and feldspar with rounded ilmenite (bright phase labelled I). (g) Granophyric clast with intergrowth of quartz and K-feldspar. (h) Granophyric clast 7 with intergrowth of quartz and K-feldspar, grain boundary with exsolved pyroxene indicated with black arrow. (i) Small granophyric clast with intergrowth of quartz and K-feldspar, grain boundary with exsolved pyroxene indicated with black arrow. Bright regions in cracks are Au-coat.

Mineral chemistry plots

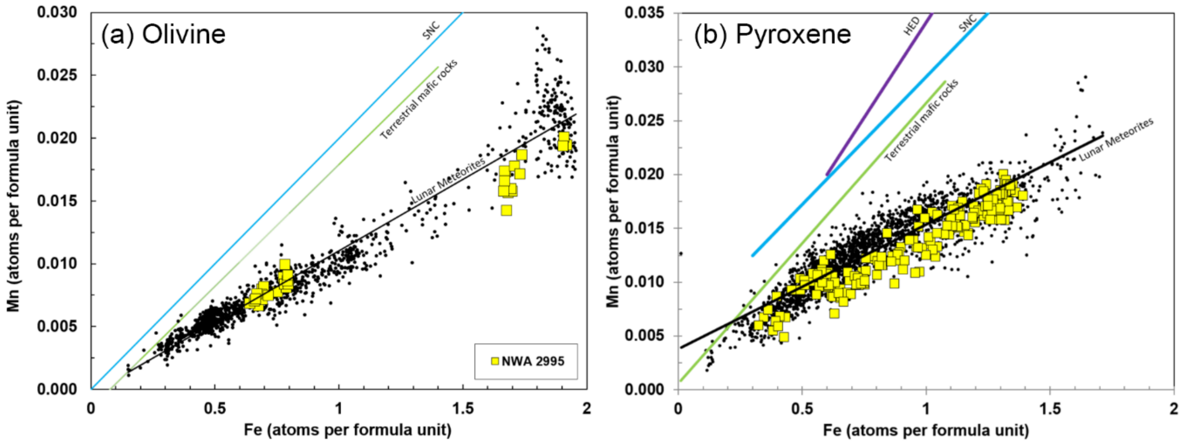

Supplementary Figure 5**.** Fe/Mn ratios in (a) olivine and (b) pyroxene in NWA 2995 compared with Fe/Mn lines from other terrestrial planetary bodies including Mars (SNC meteorites), Vesta (HED meteorites), Earth mafic rocks, and other lunar meteorites (for sources of data see *Joy et al., 2014*).

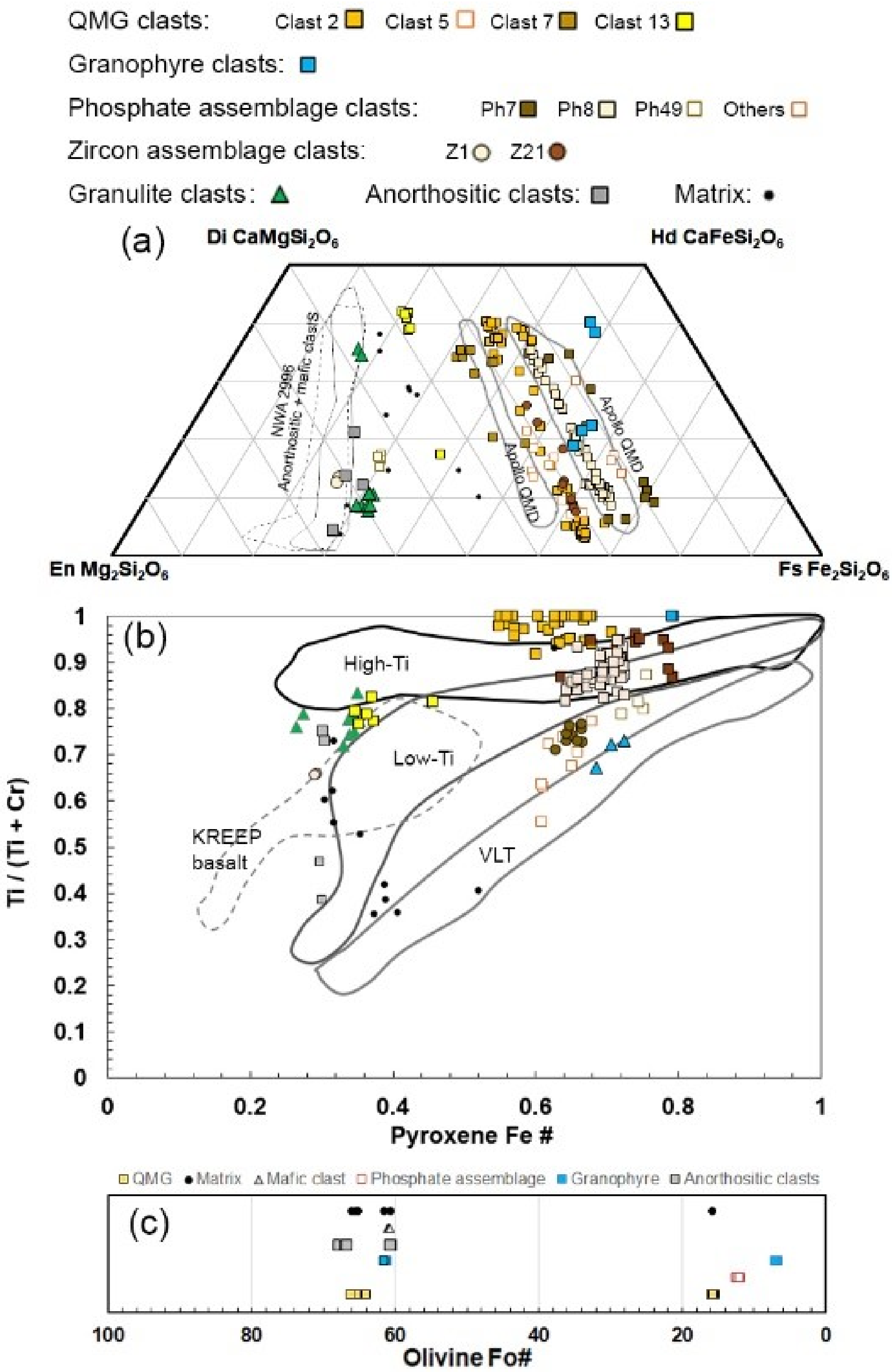

Supplementary Figure 6. Mineral chemistry of clasts in NWA 2995. (a) Pyroxene quadrilateral, where the NWA 2996 anorthositic and mafic clast field is taken from *Mercer et al. (2017)* and examples of pyroxene compositions in Apollo quartz monzodiorites (plotted as fields with solid grey outlines) are from *Jolliff (1991)*. (b) Pyroxene Ti# (Ti/(Ti+Cr)) vs Fe# plot where NWA 2995 clast data are compared with the Apollo mare basalt and KREEP basalt ranges (data fields taken from Astromat database https://www.astromat.org/). (c) Range of olivine forsterite content in clasts in NWA 2995.

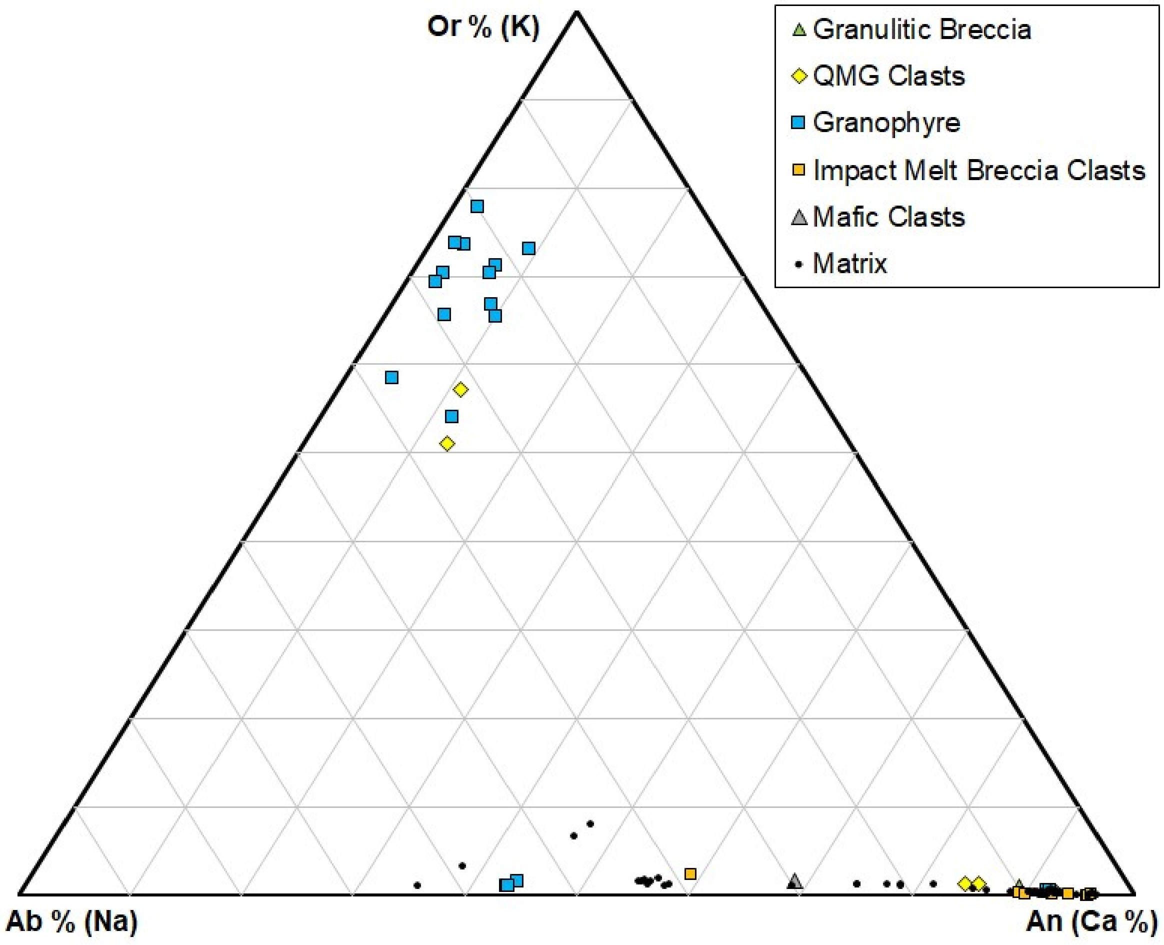

Supplementary Figure 7**.** Plagioclase chemistry of clasts and matrix grains in NWA 2995.

Raman spectroscopy

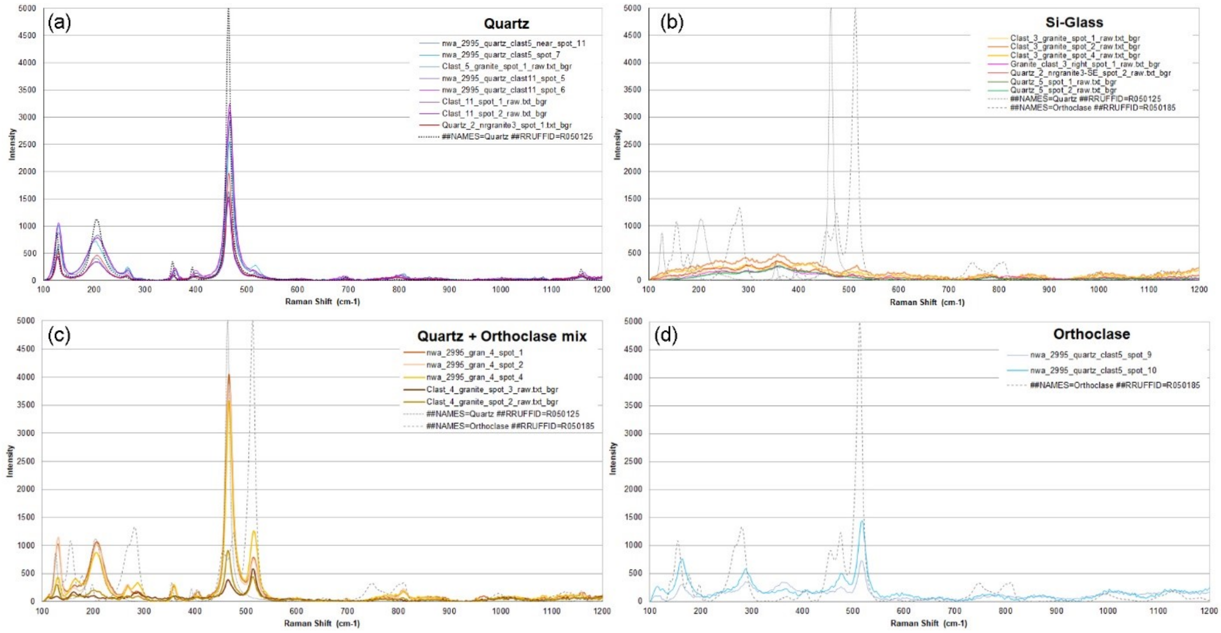

Supplementary Figure 8. Raman spectra from Si-rich phases in granophyric clasts and monomineralic Si-phase clasts in NWA 2995. (a) Quartz, (b) Si-rich glass, note the lower reflectance intensity, (c) spots analysed that were a mixture of quartz and orthoclase, and (d) orthoclase. Reference quartz and orthoclase data shown with dashed lines are from the RRUFF database. Si-rich phase Raman data are provided in Supplementary Table 9.

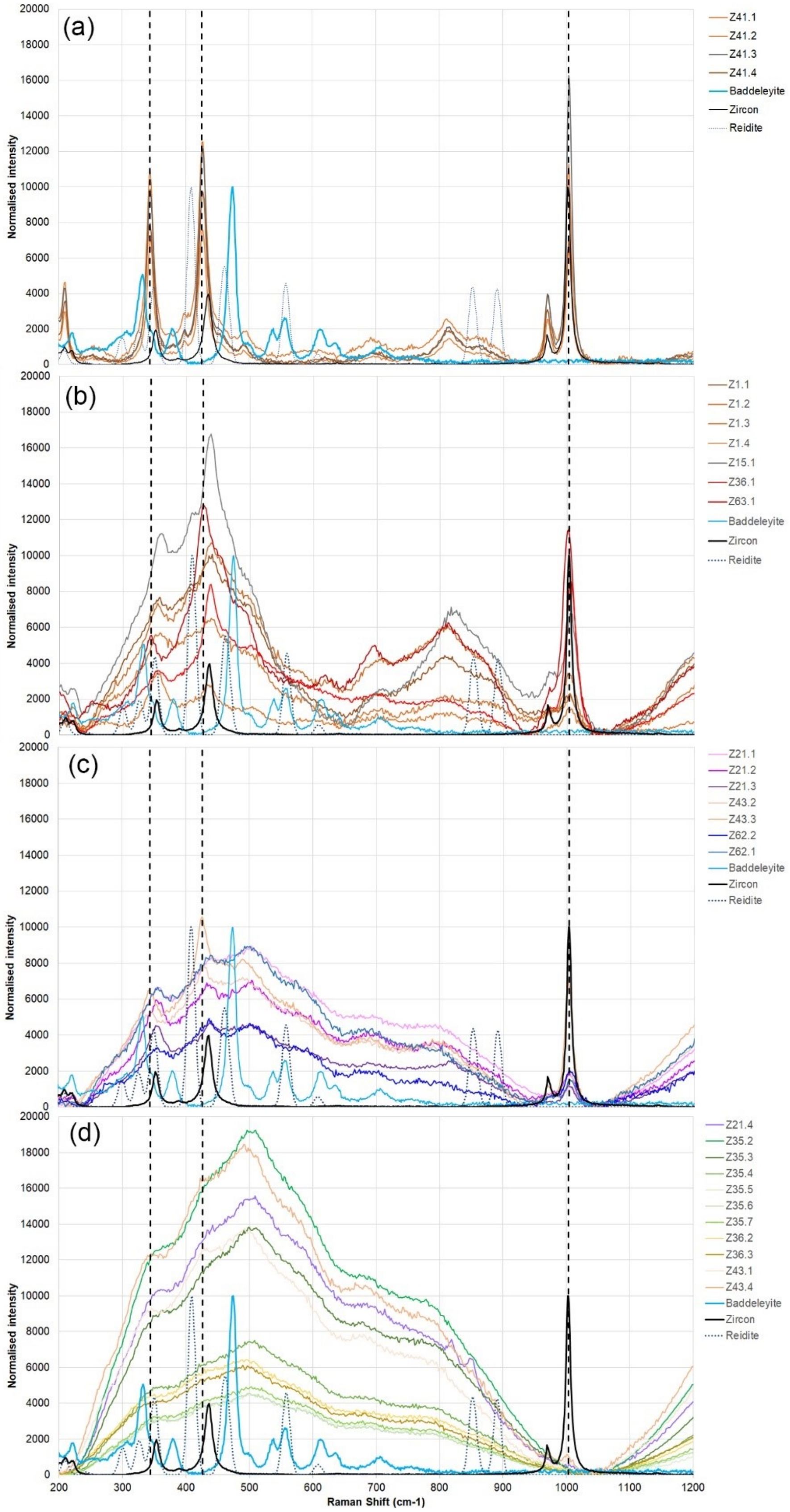

Supplementary Figure 9. Raman spectra from zircon in NWA 2995. (a) Relatively unmodified with 344, 426 and 1003 cm^{-1} bands, (b) intermediate modified with less intense band at 1003

cm^{-1} and right shifted bands from 344 or 1003 cm^{-1}, (c) modified spectra with little no band at 1003 cm^{-1}, and appearance of a broad peak around 504 cm^{-1}, and (d) highly disordered with no 344 or 1003 cm^{-1} bands and a very broad peak centred around 504 cm^{-1}. Reference spectra for zircon (R050034) in black and baddeleyite (R060016) in blue are from the RRUFF database, and reidite modelled spectrum in dashed black line is from *Stangarone et al. (2019)*. Zircon Raman data are given in Supplementary Table 14.

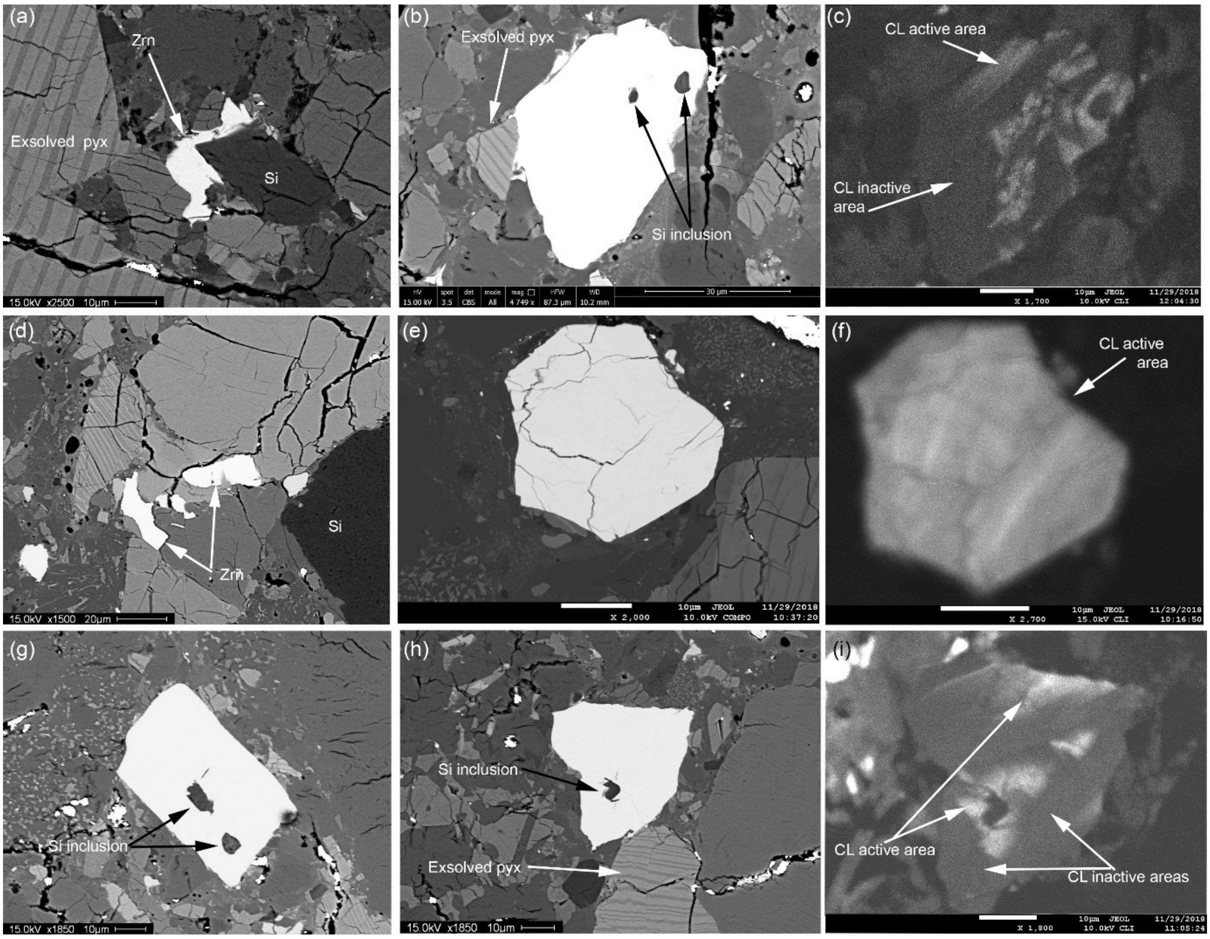

Supplementary Figure S10. Examples of zircon in NWA 2995 where zircon are the bright white phases in the BSE images (left and middle columns). (a) Zircon grain #20, associated with large QMG Clast 2. (b) BSE image and (c) CL image of Zircon grain #21 that is associated with an exsolved pyroxene. (d) Zircon grain #48, associated with a QMG clast. (e) BSE image and (f) CL image of Zircon grain #41 associated with exsolved pyroxene from a QMG clast. (g) Zircon grain #44, found in the sample matrix. (h) BSE image and (i) CL image of Zircon grain #43, associated with exsolved pyroxene from a QMG clast. Note that images (c) and (f) have been modified from original to reorient the scale bar as the images were taken in a different orientation. Note also that some of the bright white phases within the samples cracks is residual Au sample coat.

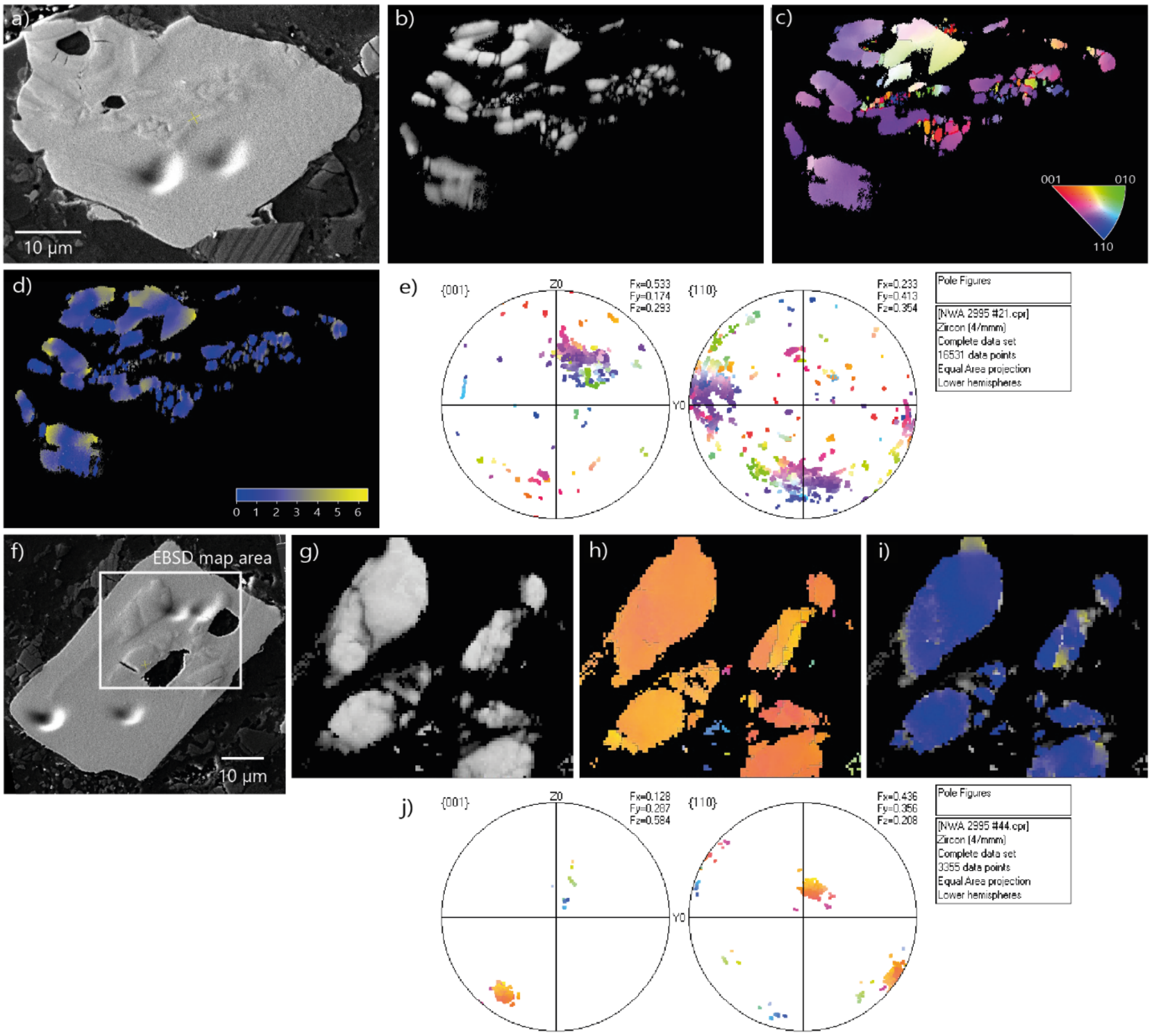

Supplementary Figure 11. Electron backscatter diffraction (EBSD) data for NWA 2995 zircons. (a) BSE image of zircon #21 – note that small indents in the grain are SIMS spots locations: see Supplementary File 3; (b) EBSD band contrast map (100 nm step size) of the same area highlighting strongly diffracting domains (which are CL-active) surrounded by zircon with little to no detectable diffraction signal (CL-inactive); (c) inverse pole-figure (IPF) map showing deformed zircon host with low angle (2-10°; grey) boundaries, and <3 µm neoblasts with variable crystallographic orientations separated by high angle (>10°; red) ”grain” boundaries, (d) grain relative orientation deviation (GROD) map showing low-strain nature of neoblasts, and (e) pole figures showing scattered orientations of neoblasts. (f) BSE image of zircon #44 showing EBSD map area – note that small indents in the grain are SIMS spots locations: see Supplementary File 3; (g) band contrast, (h) IPF, and (i) GROD maps (300 nm step size; same legends as c and d) showing highly deformed host zircon, with <5 µm variably oriented domains; (j) pole figures highlight strained nature of host zircon. Limited data points for variably oriented domains make interpretation of their significance more challenging than in grain #21.

Supplementary Figure References

Jolliff, B. L. 1991. Fragments of quartz monzodiorite and felsite in Apollo 14 soil particles. *Lunar and Planetary Science Conference, 21st, Houston, TX, Mar. 12-16, 1990, Proceedings* (A91-42332 17-91). Houston, TX, Lunar and Planetary Institute, 1991, p. 101-118. vol. 21, pp. 101-118.

Joy, K.H., Crawford, I.A., Huss, G.R., Nagashima, K. and Taylor, G.J., 2014. An unusual clast in lunar meteorite MacAlpine Hills 88105: A unique lunar sample or projectile debris? *Meteorit. Planet. Sci.*, 49(4), pp.677-695.

Mercer, C.N., Treiman, A.H. and Joy, K.H., 2013. New lunar meteorite Northwest Africa 2996: A window into farside lithologies and petrogenesis. *Meteorit. Planet. Sci.*, *48*(2), pp.289-315.

Stangarone, C., Angel, R.J., Prencipe, M., Mihailova, B. and Alvaro, M., 2019. New insights into the zircon-reidite phase transition. *Am. Mineral.*, 104(6), pp.830-837.

Supplementary Figure 12 (a-n). BSE images showing the location of NWA 2995 Phosphate U-Pb points collected in Beijing. Ap = apatite and M = merrillite lables in image identified by SEM EDS analysis. Note that phosphates in panes (l), (m) and (n) do not fall on the U-Pb isochron. Data in Supplementary Table 10.

Supplementary Figure 12a. Apatite Grain #22

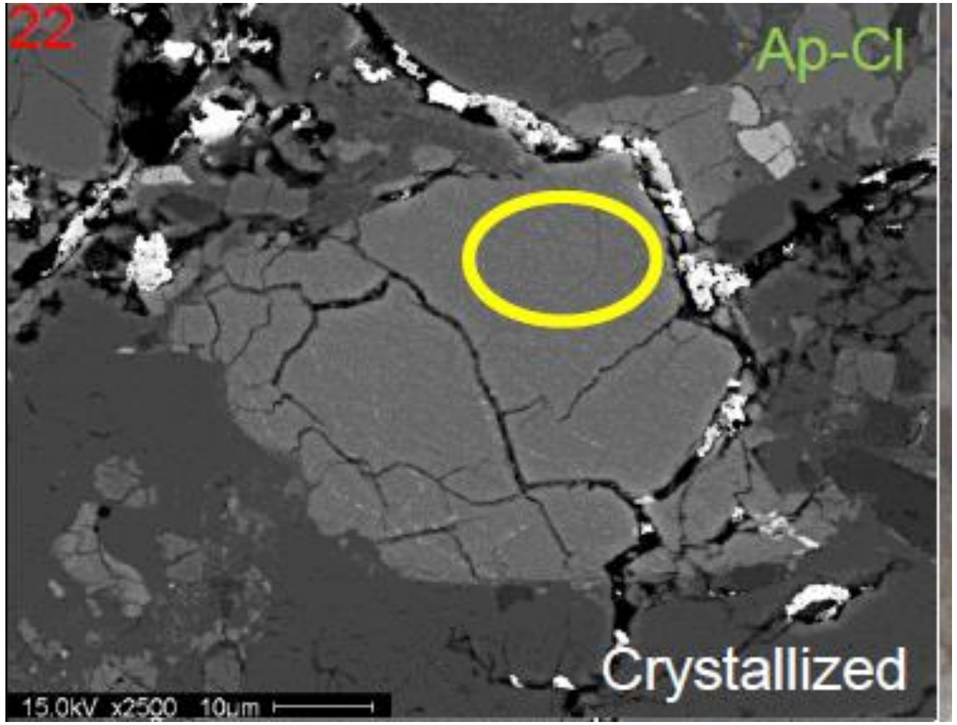

Close up of area in yellow square at right.
SIMS spot location shown as yellow oval.

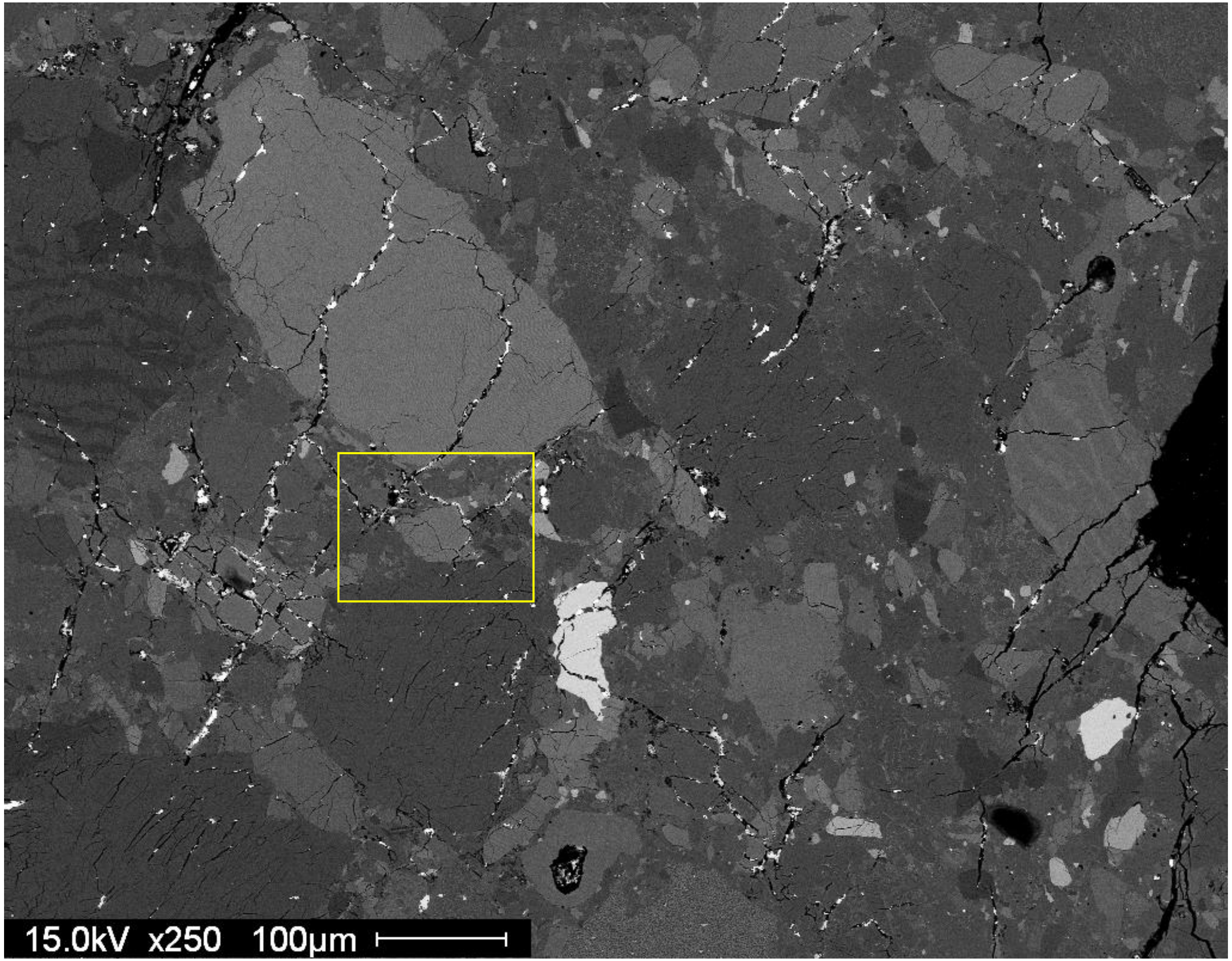

Supplementary Figure 12b. Apatite Grain #23

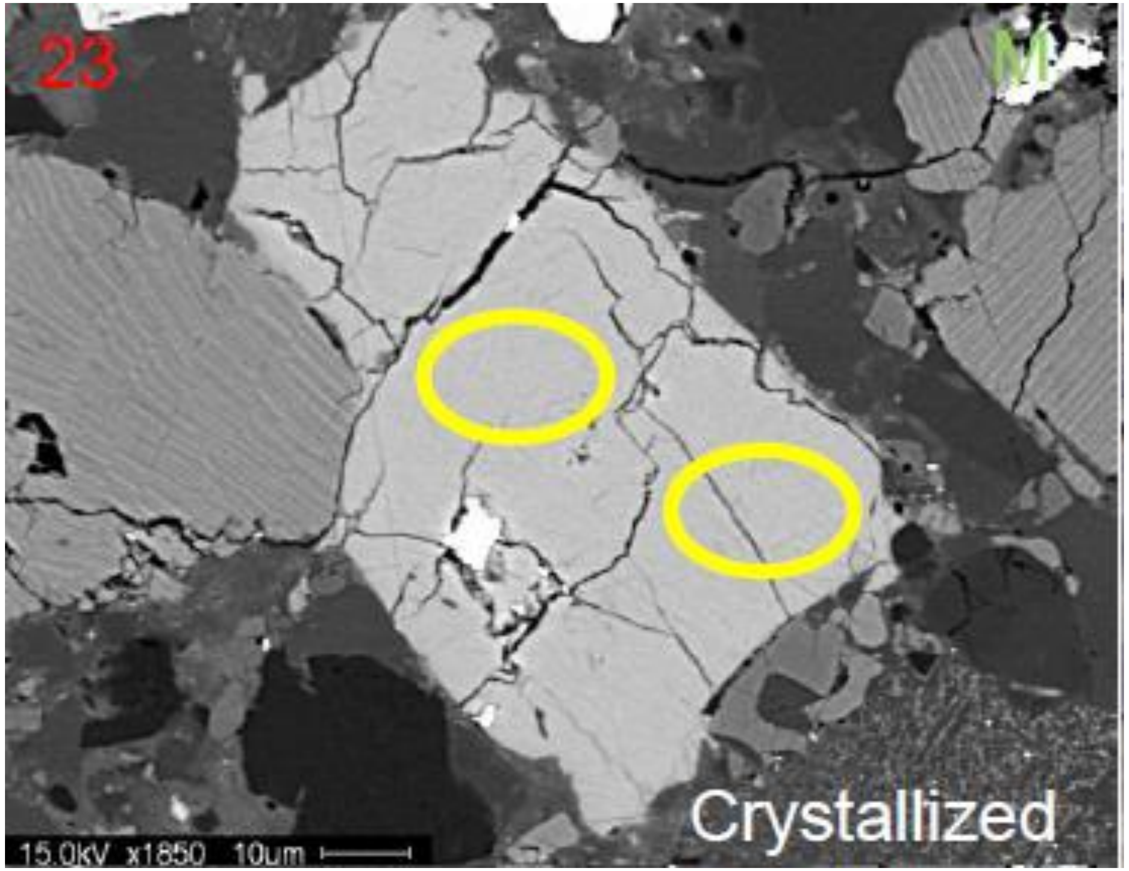

Close up of area in yellow square at right. SIMS spot locations shown as yellow ovals.

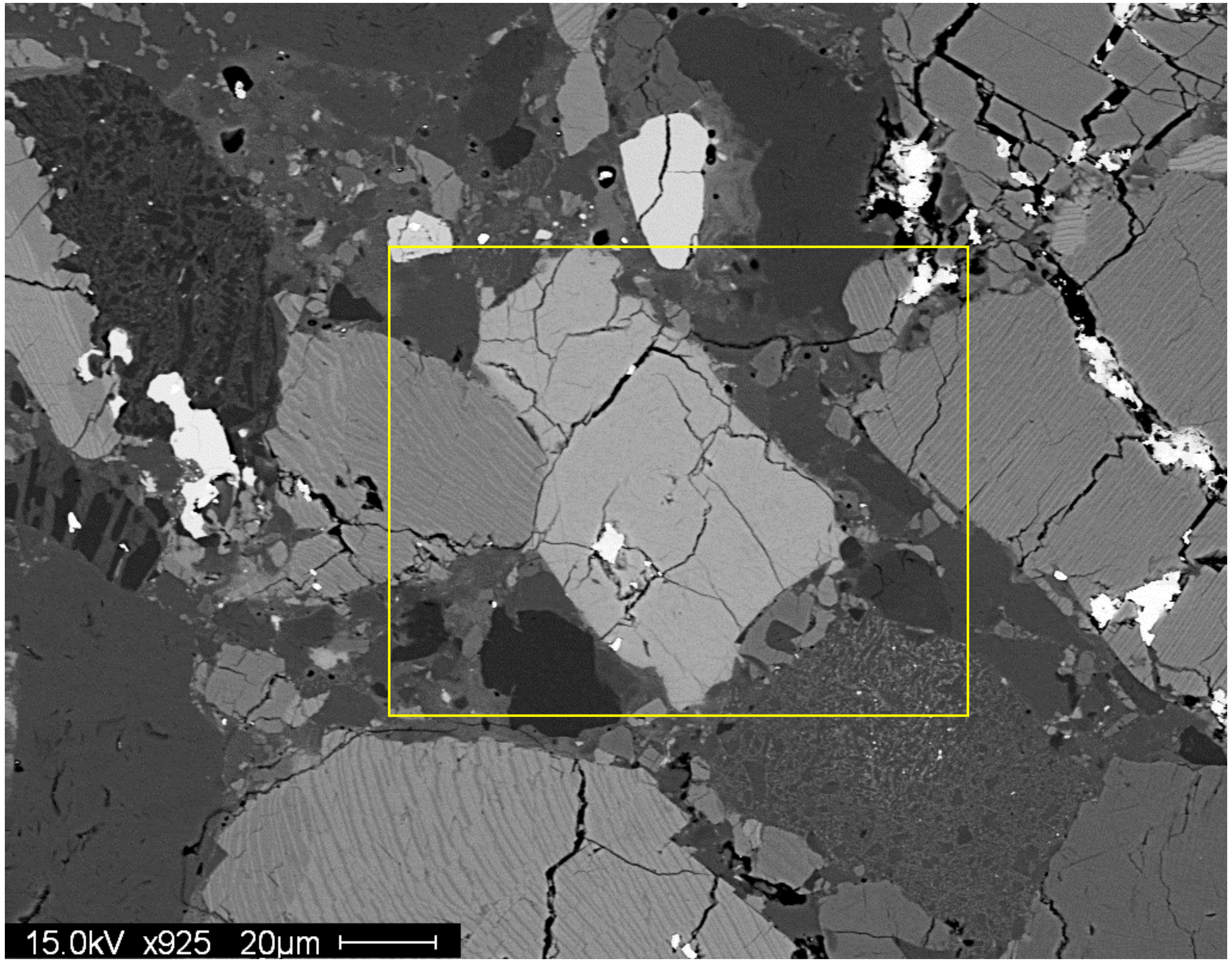

Supplementary Figure 12c. Apatite Grain #24

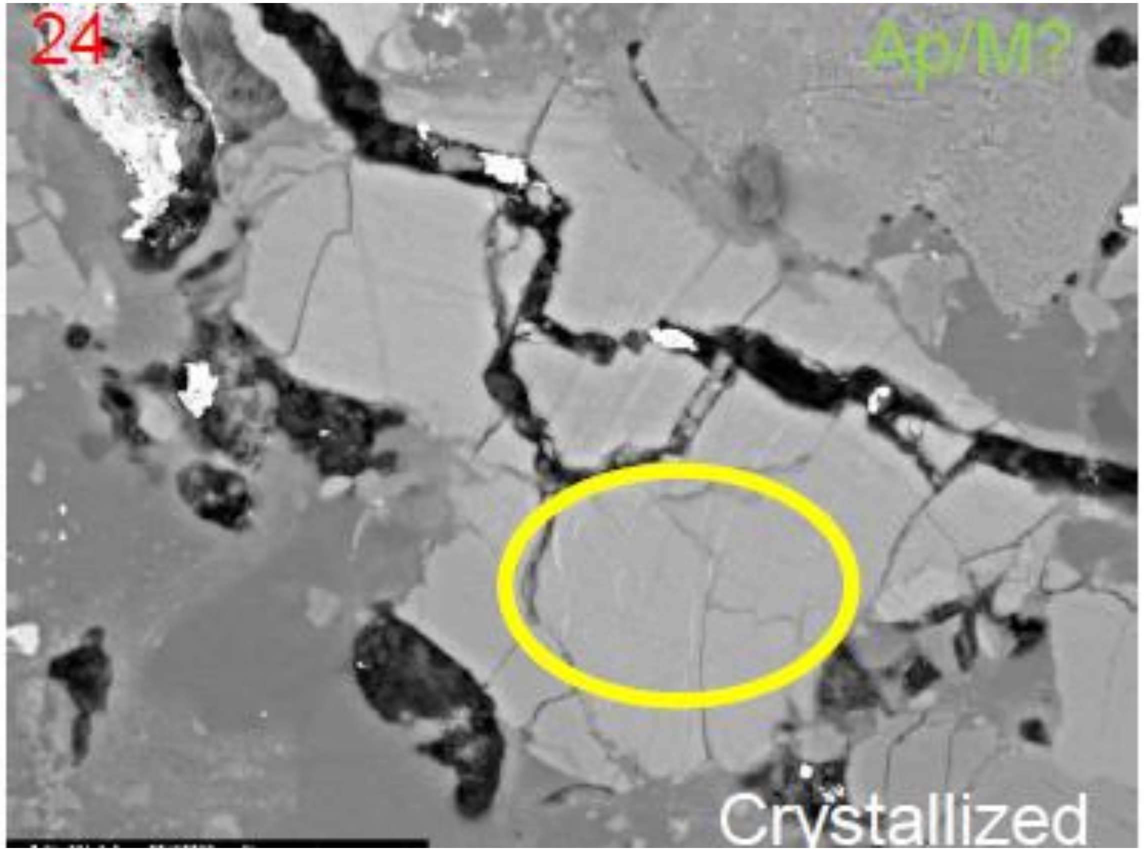

Close up of area in yellow square at right. SIMS spot location shown as yellow oval.

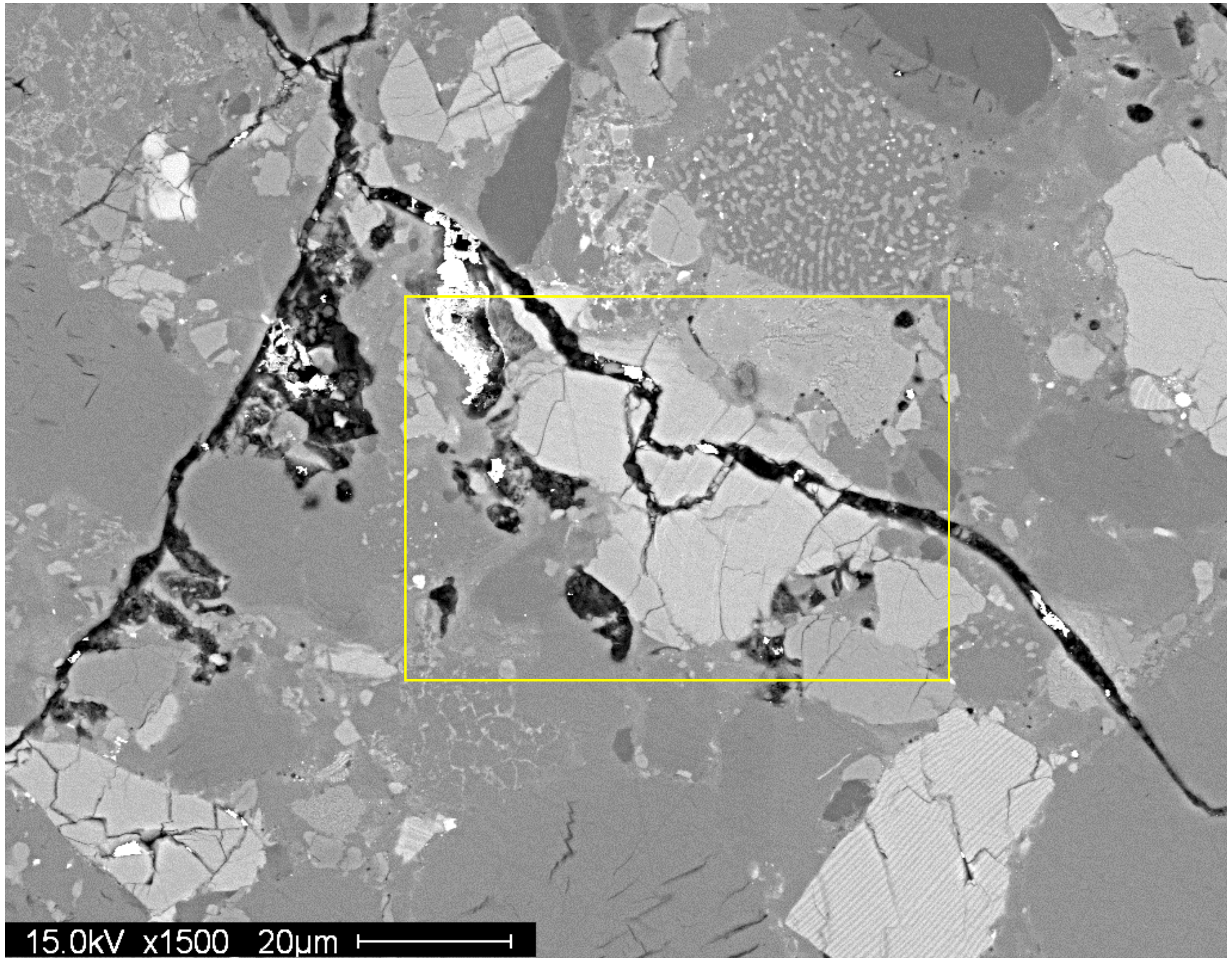

Supplementary Figure 12d. Apatite Grain #28

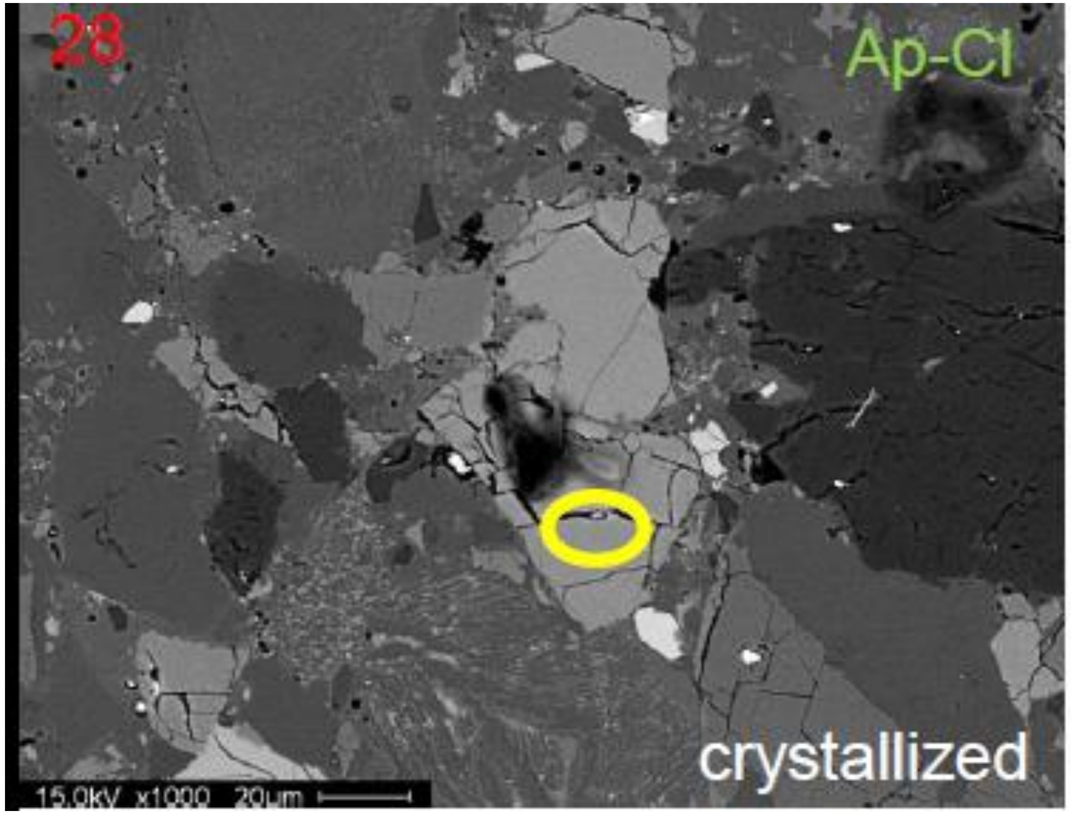

Close up of area in yellow square at right. SIMS spot location shown as yellow oval.

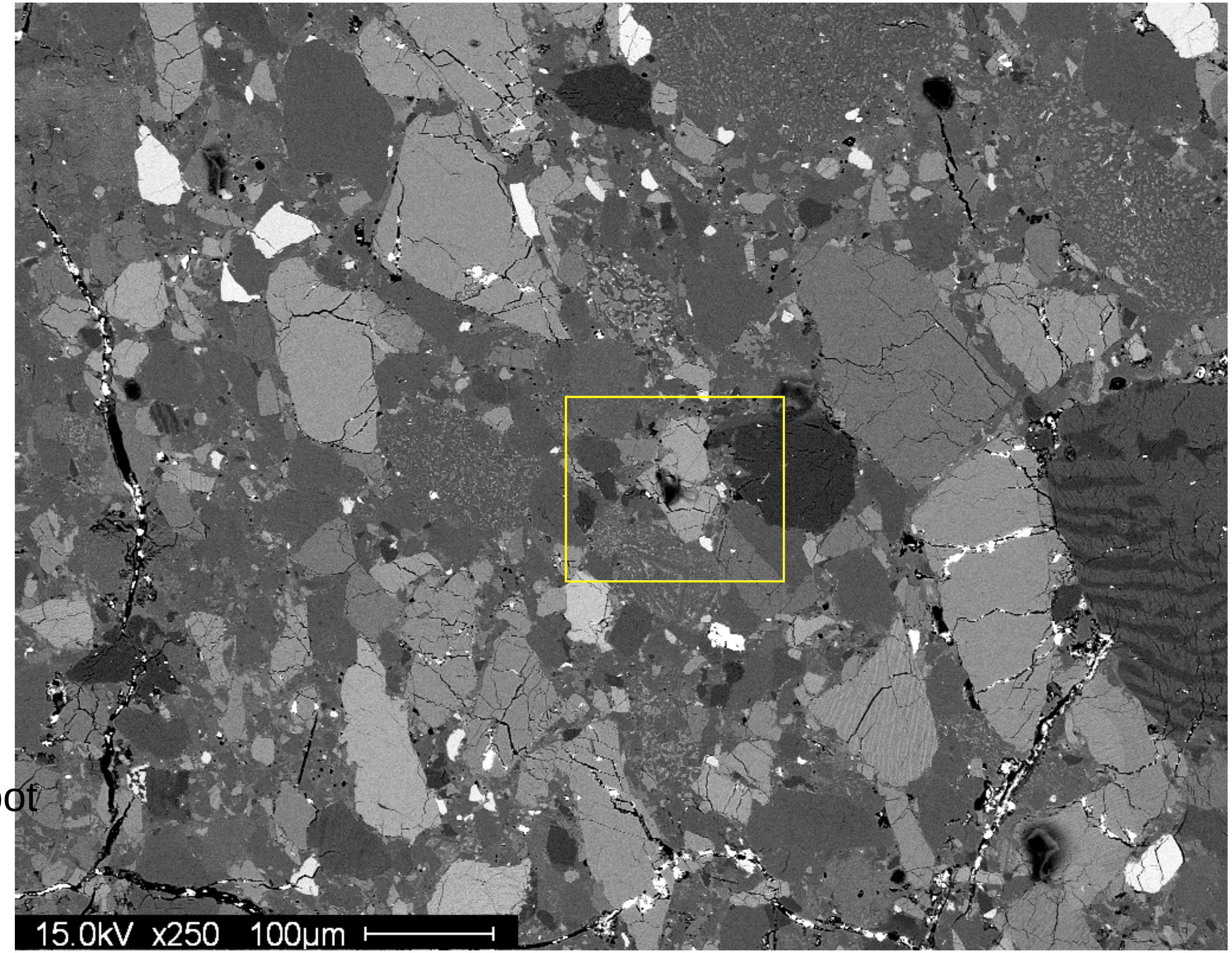

Supplementary Figure 12e. Apatite Grain #37

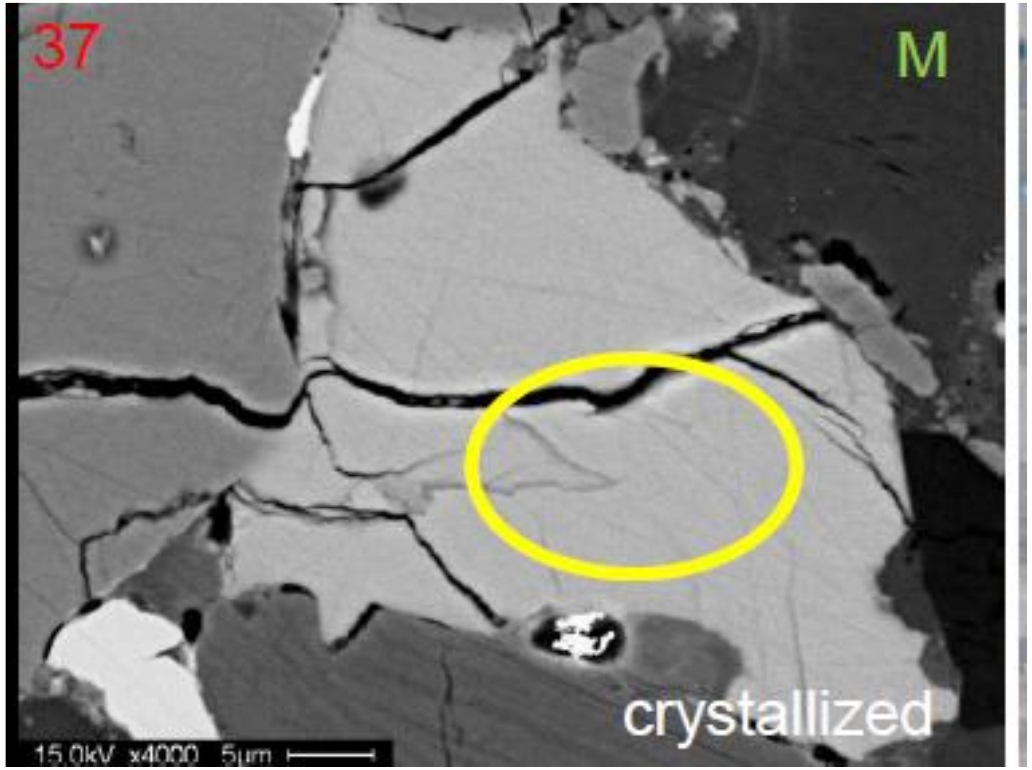

Close up of area in yellow square at right. SIMS spot location shown as yellow oval.

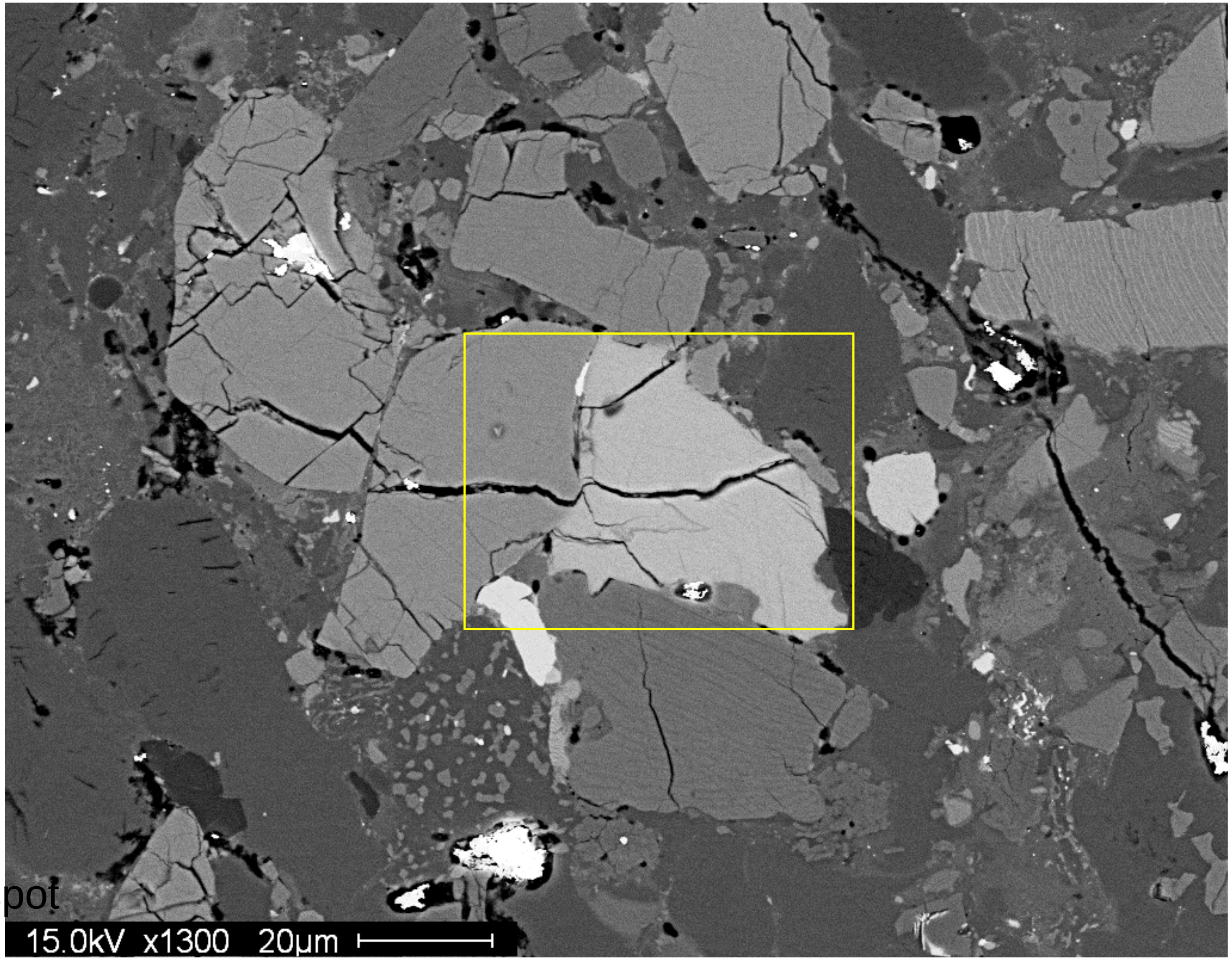

Supplementary Figure 12f. Apatite Grain #40

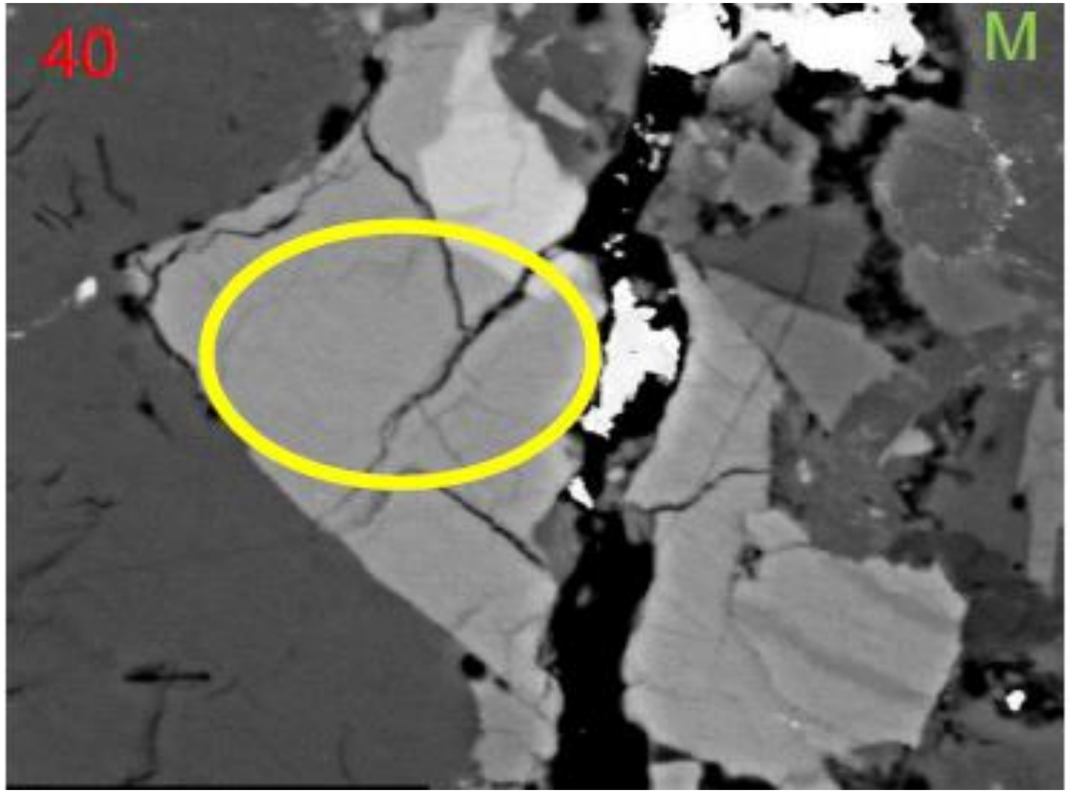

Close up of area in yellow square at right. SIMS spot location shown as yellow oval.

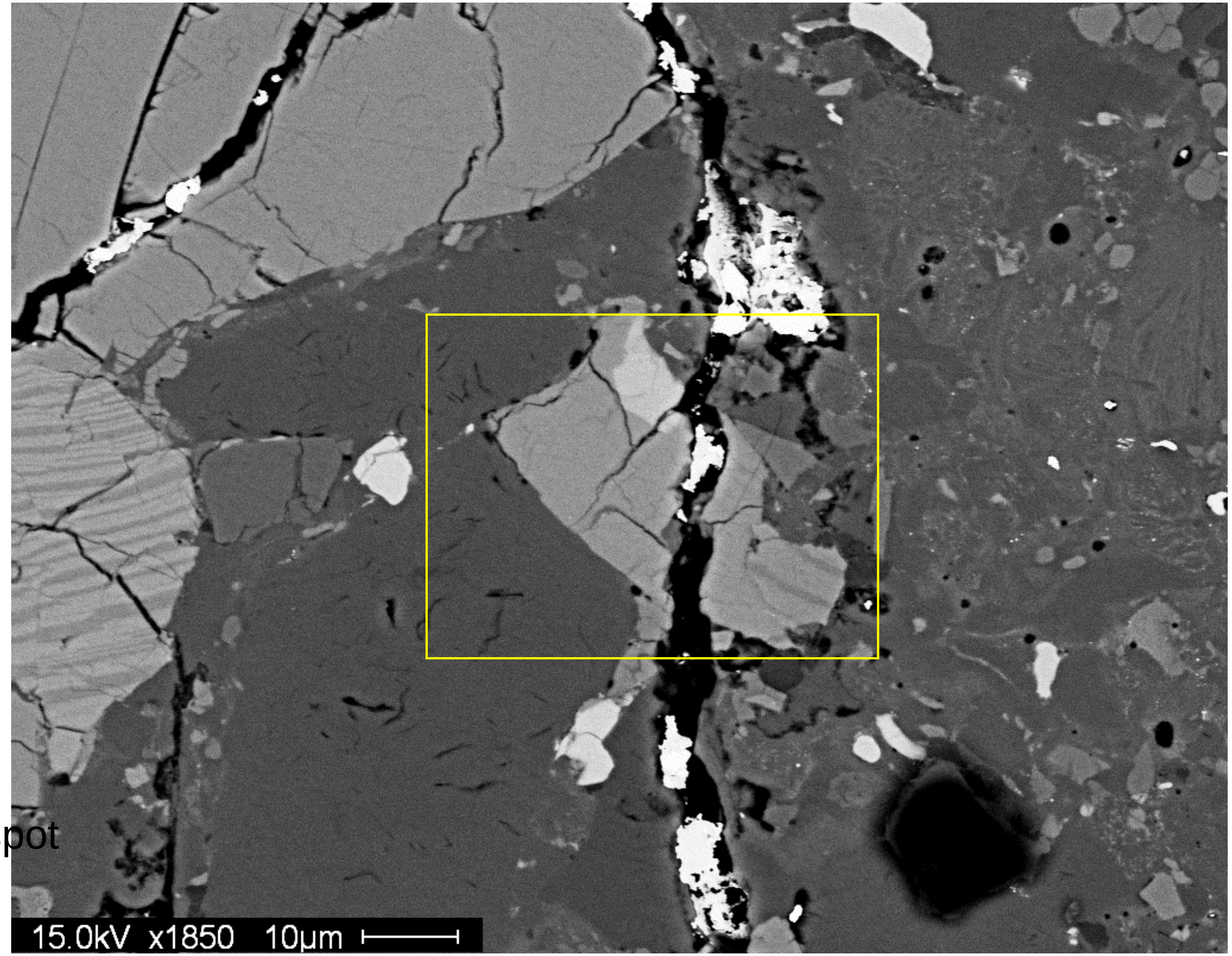

Supplementary Figure 12g. Apatite Grain #57

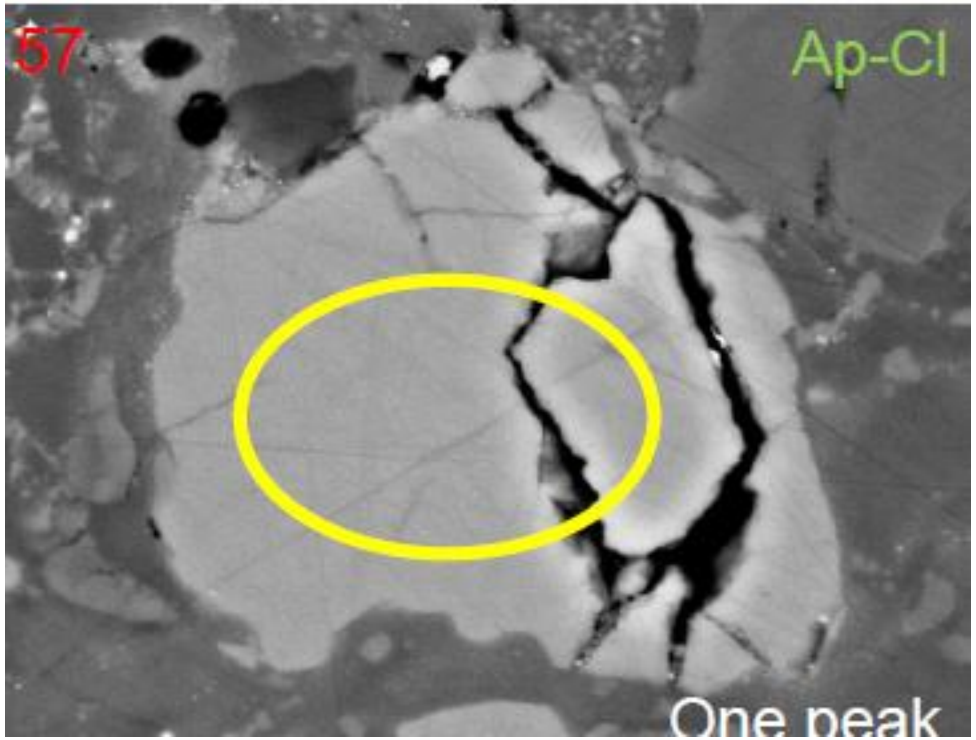

Close up of area in yellow square at right.
SIMS spot location shown as yellow oval.

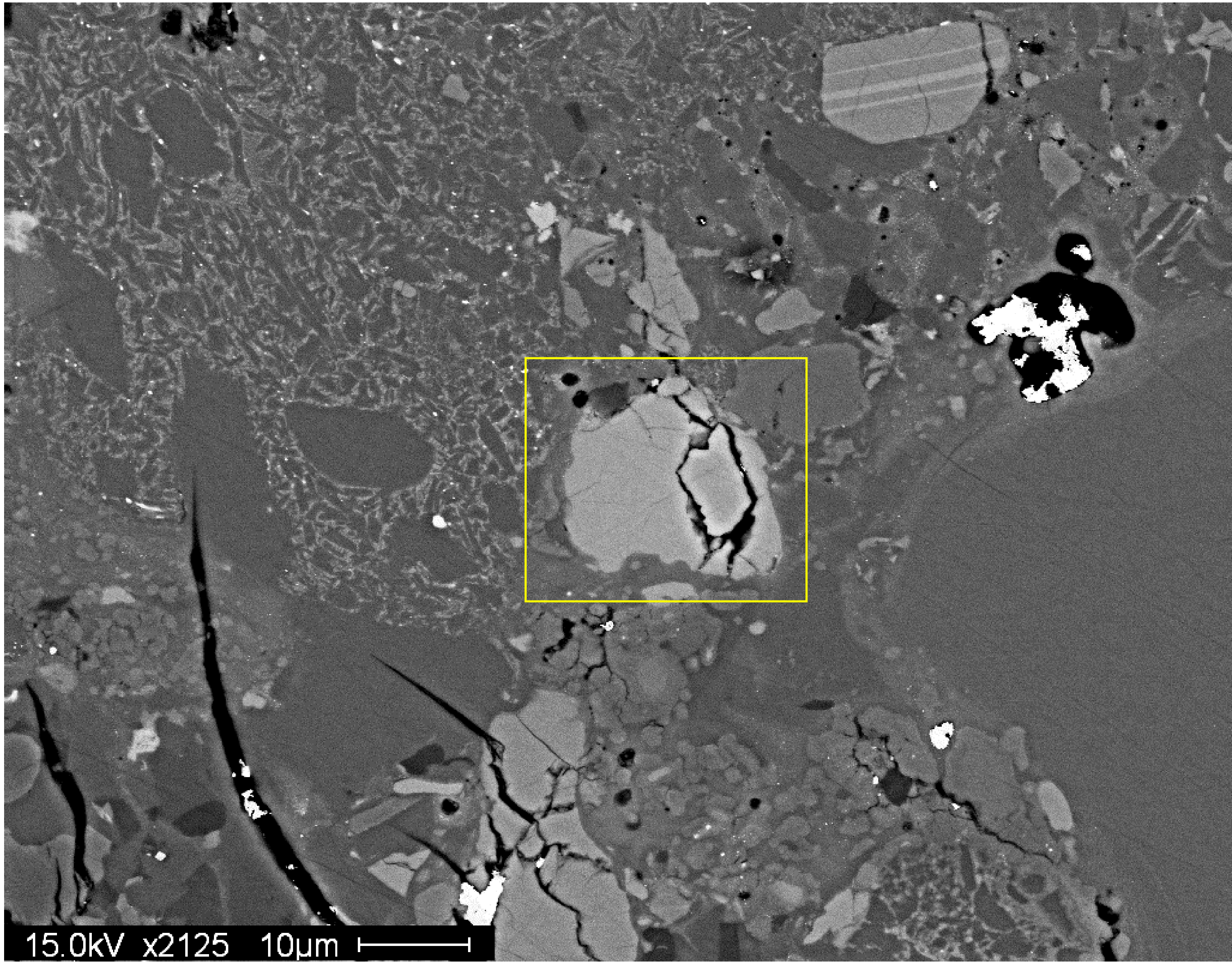

Supplementary Figure 12h. Apatite Grain #58

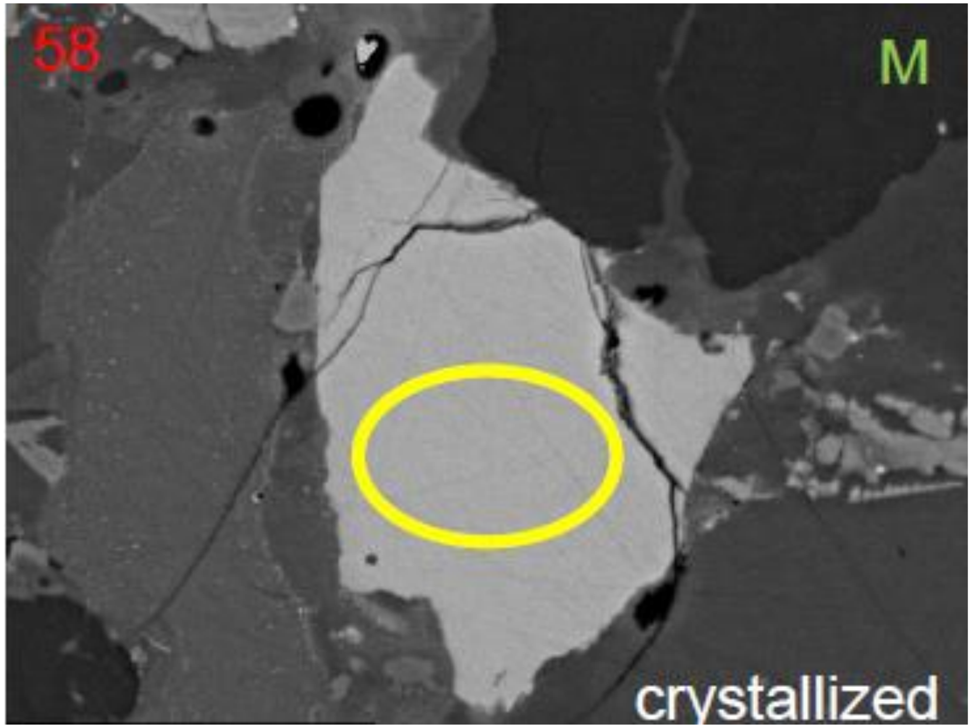

Close up of area in yellow square at right.
SIMS spot location shown as yellow oval.

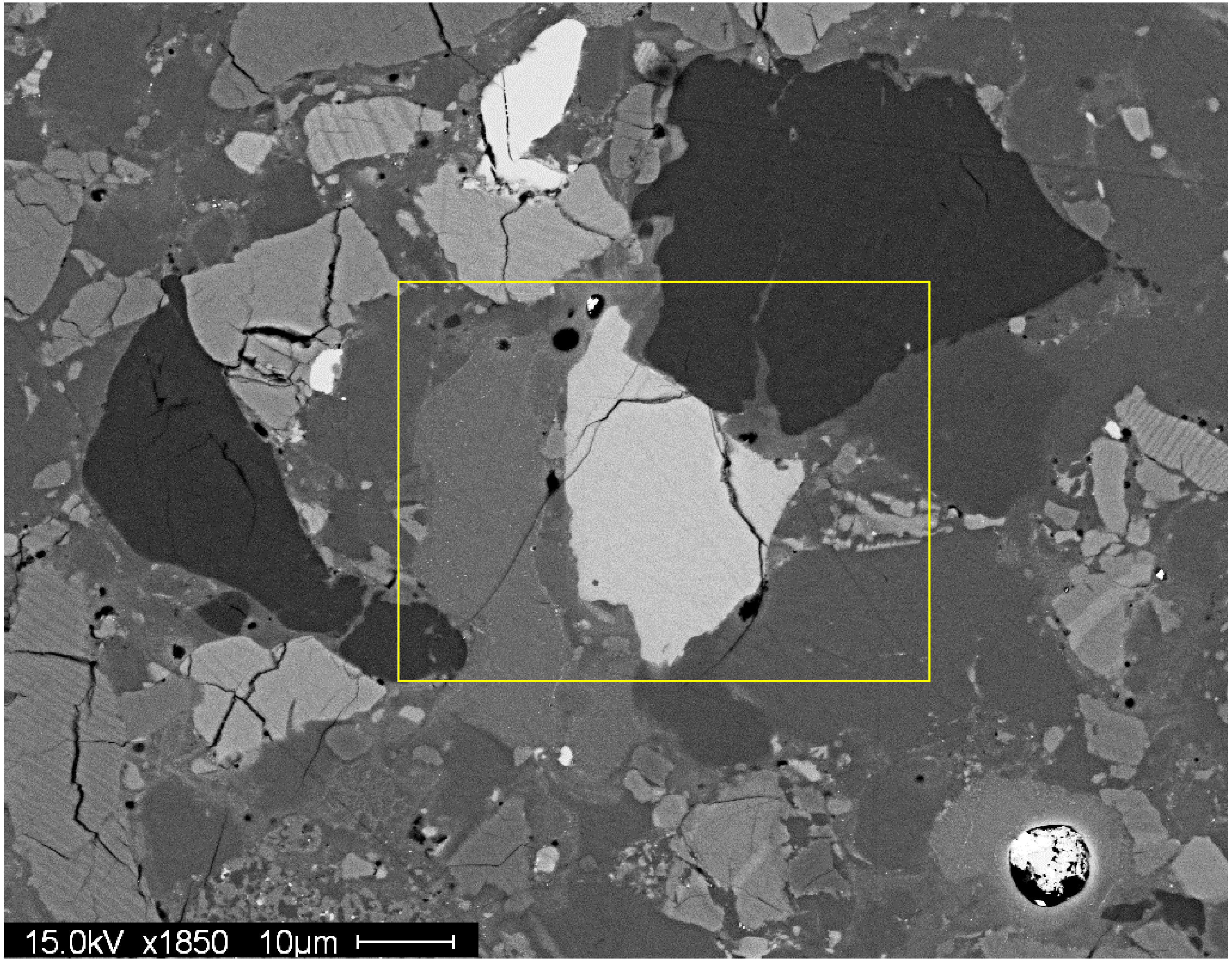

Supplementary Figure 12i. Apatite Grain #6

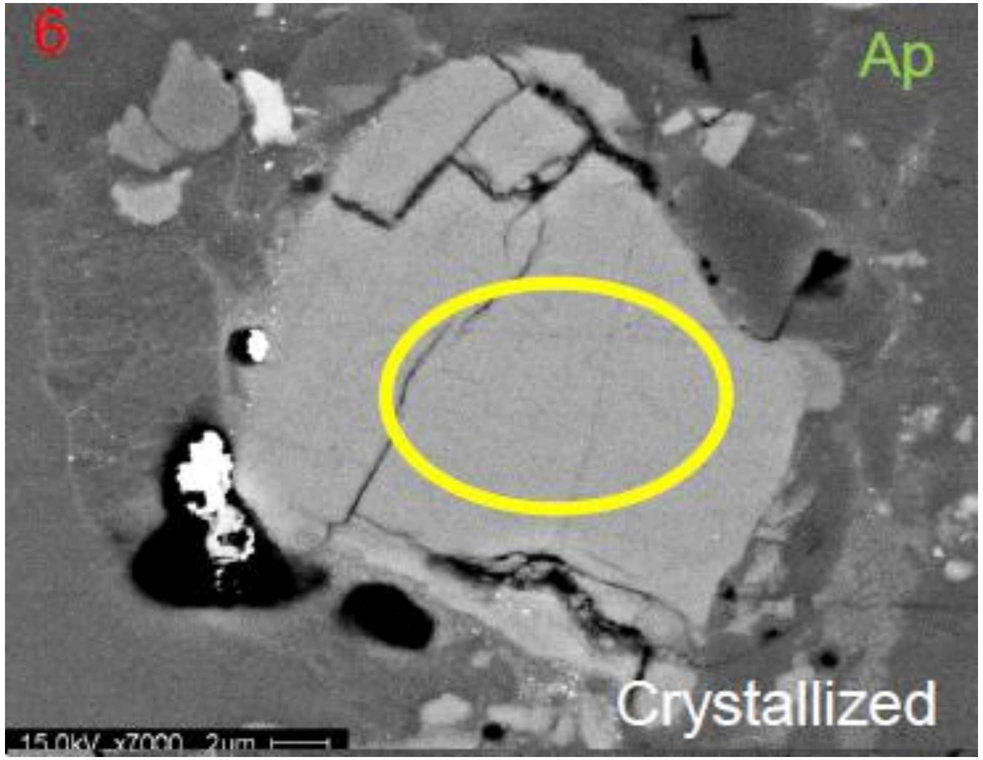

Close up of area in yellow square at right.
SIMS spot location shown as yellow oval.

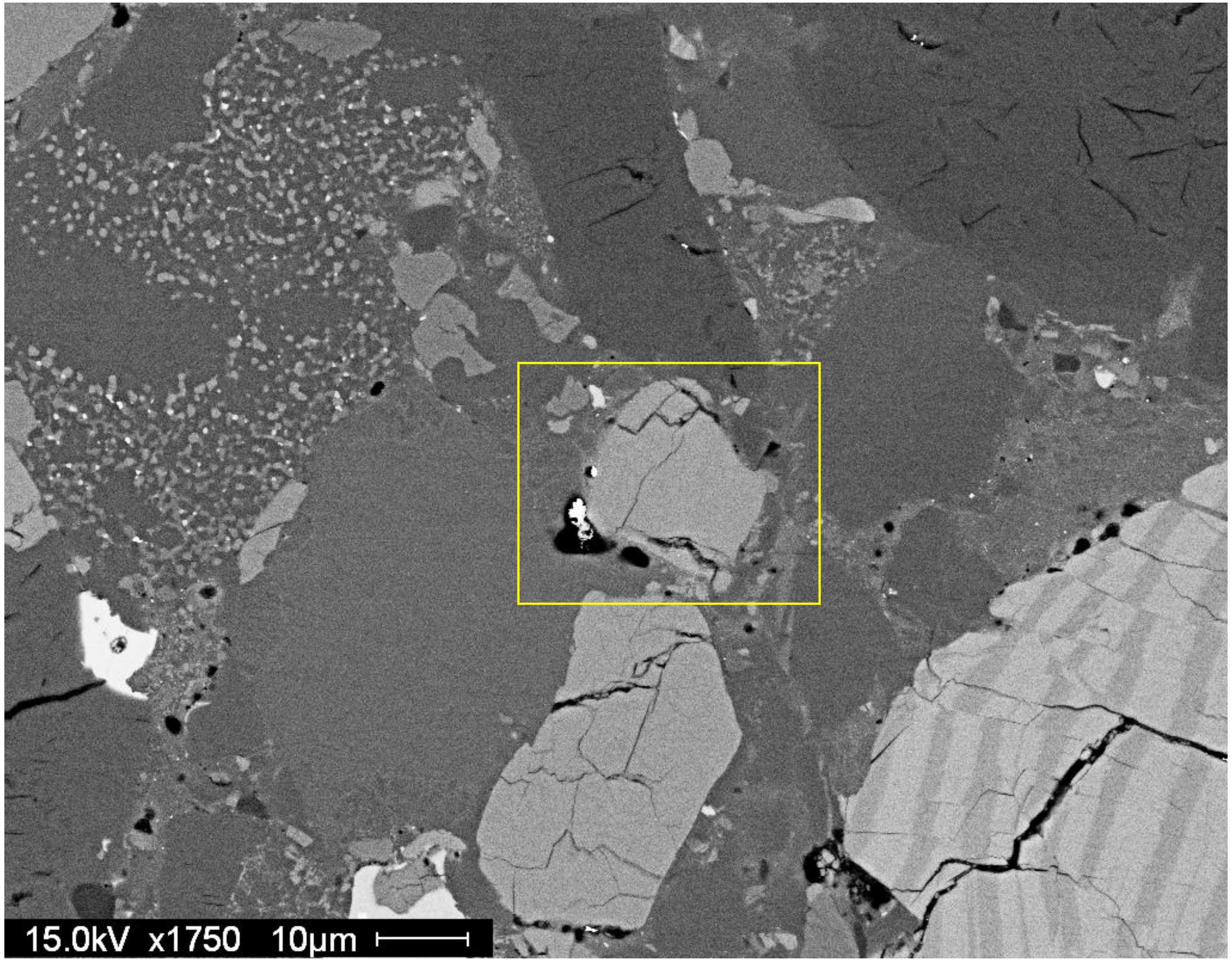

Supplementary Figure 12j. Apatite Grain #66

Close up of area in yellow square at right.
SIMS spot location shown as yellow oval.

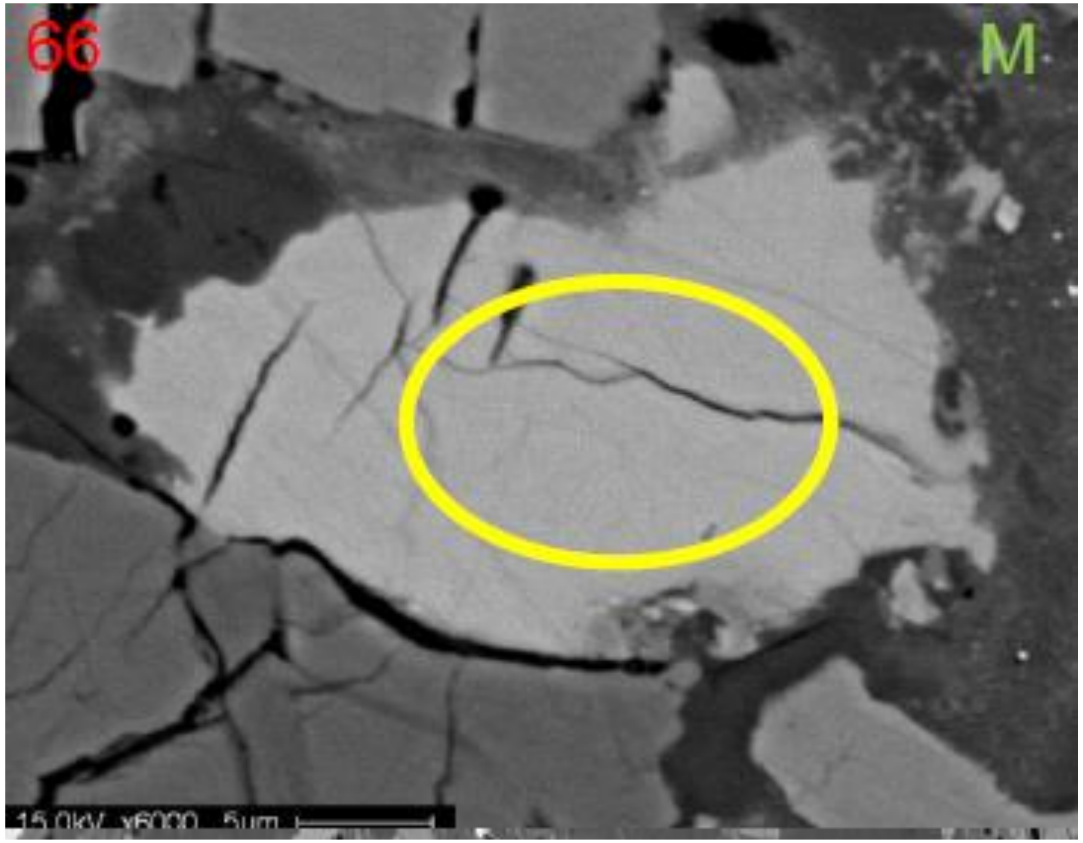

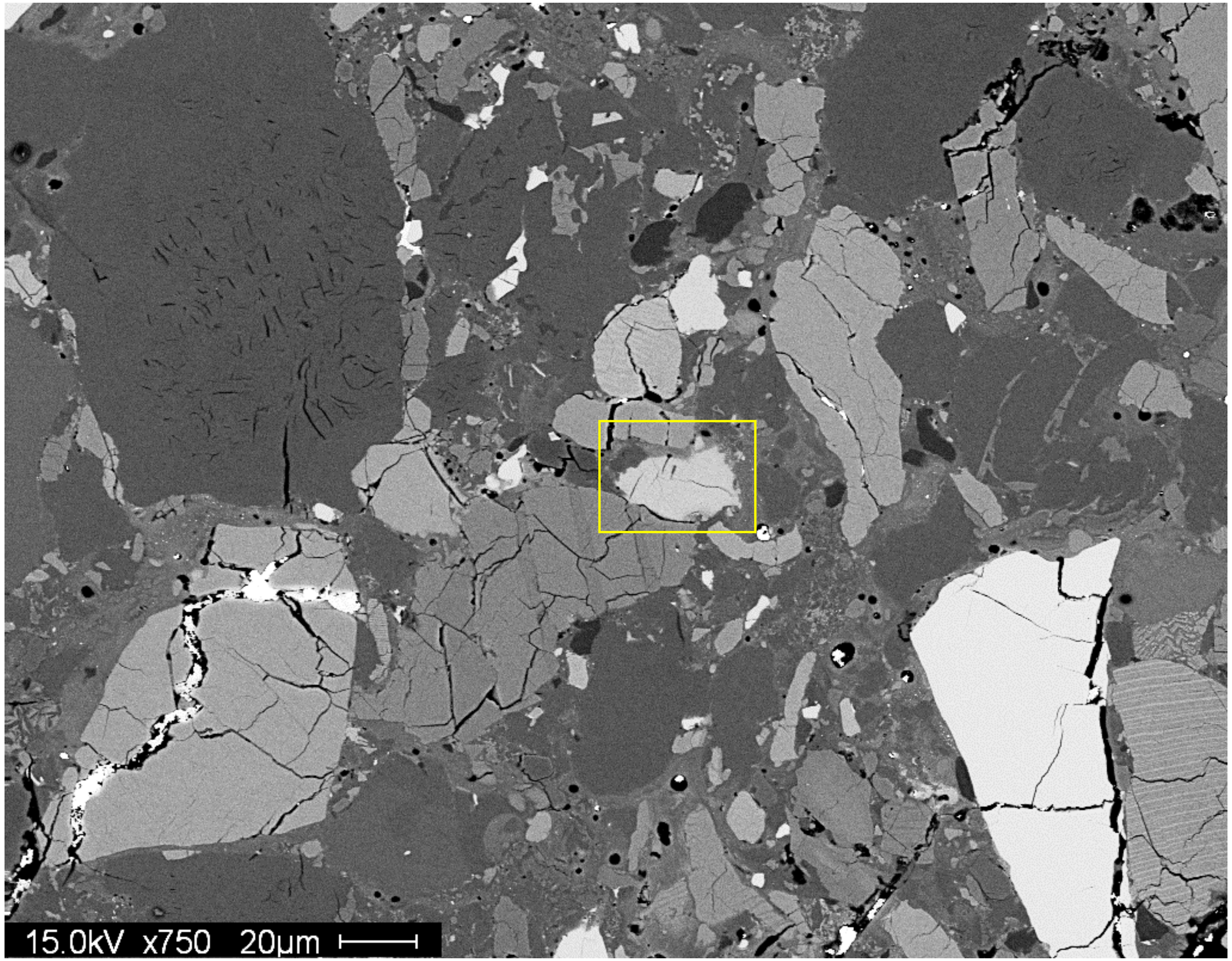

Supplementary Figure 12k. Apatite Grain #67

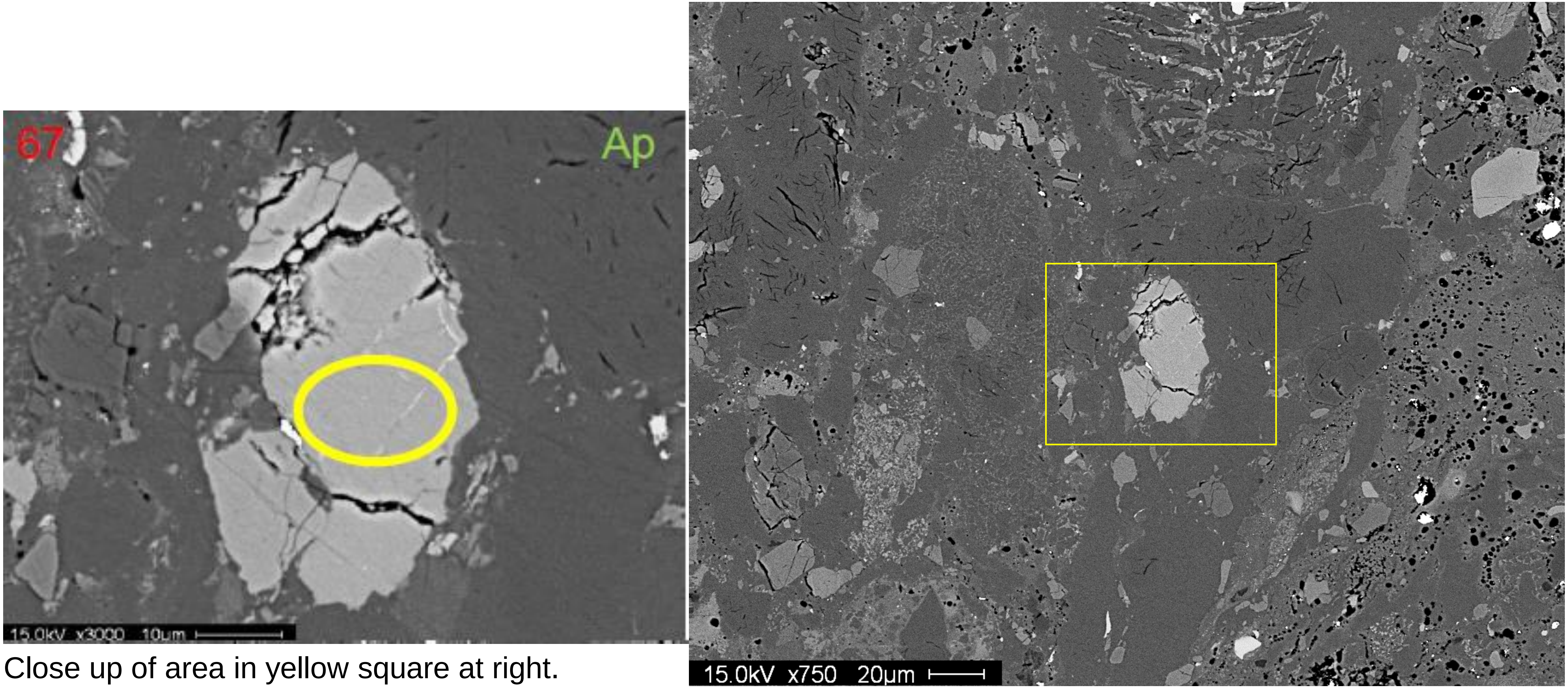

Close up of area in yellow square at right.
SIMS spot location shown as yellow oval.

Supplementary Figure 12l. Apatite Grain #33 – not on isochron

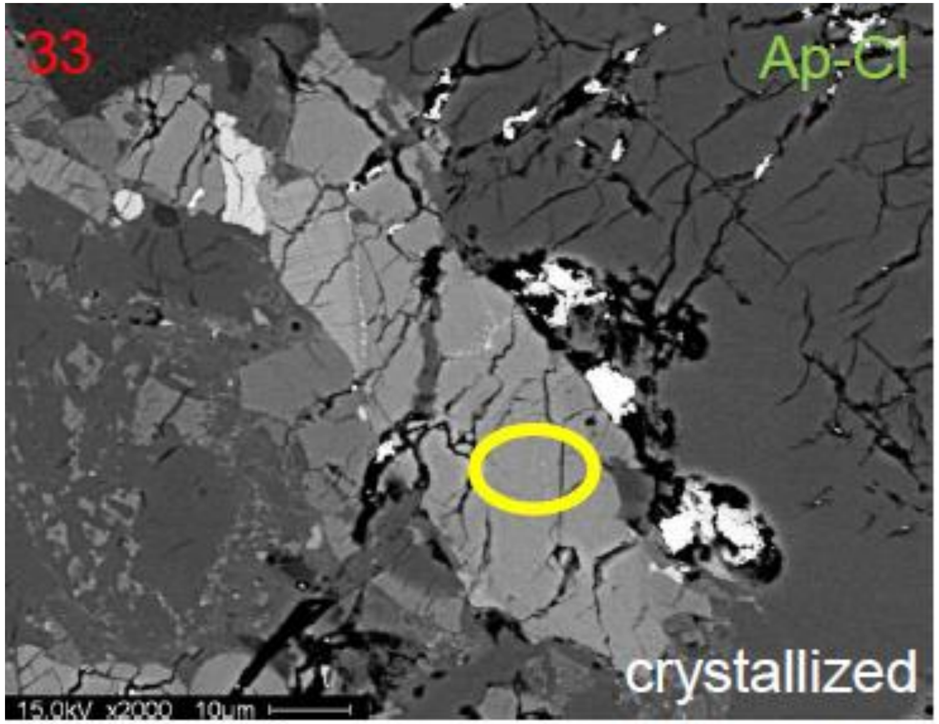

Close up of area in yellow square at right.
SIMS spot location shown as yellow oval.

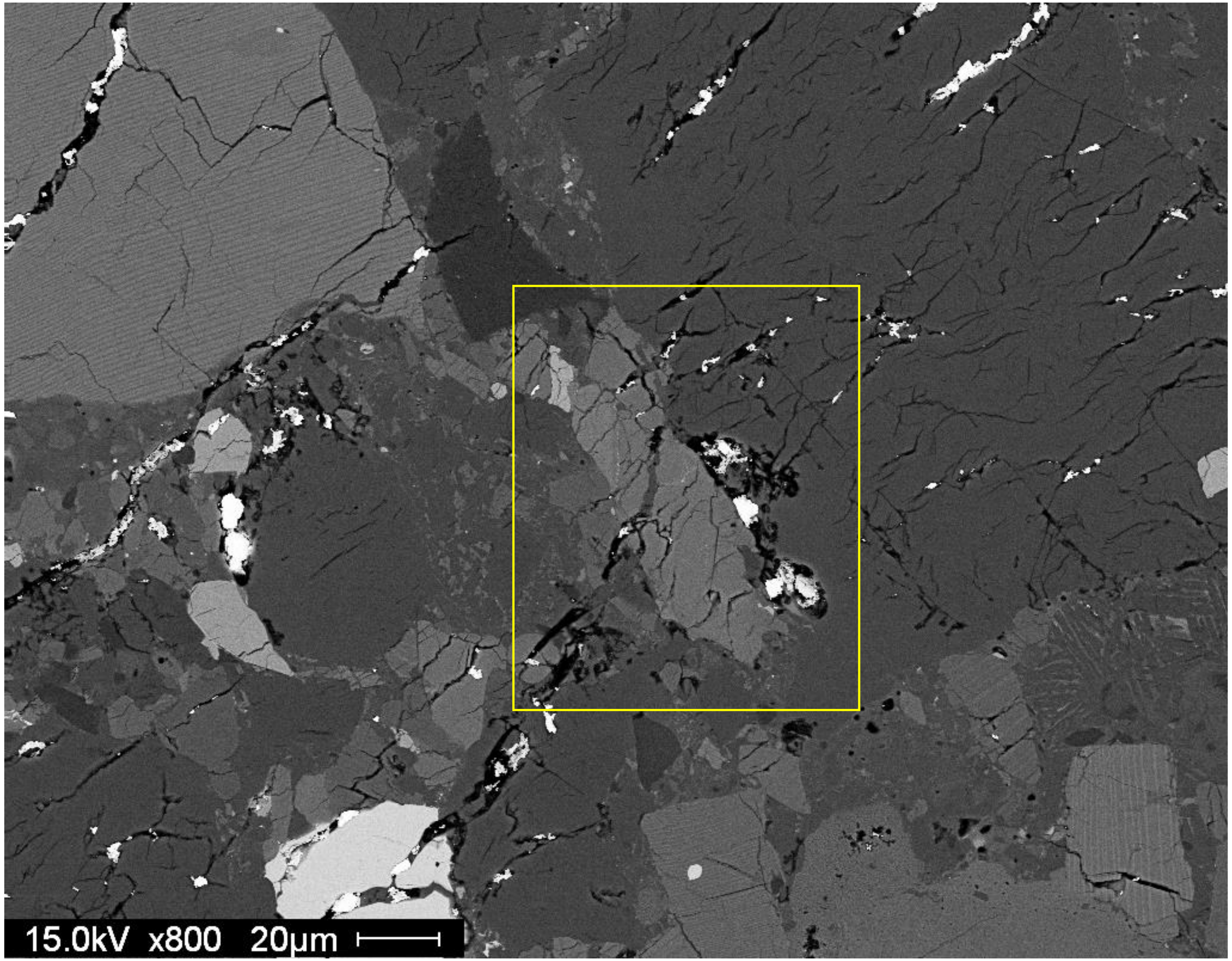

Supplementary Figure 12m. Apatite Grain #34 – not on isochron

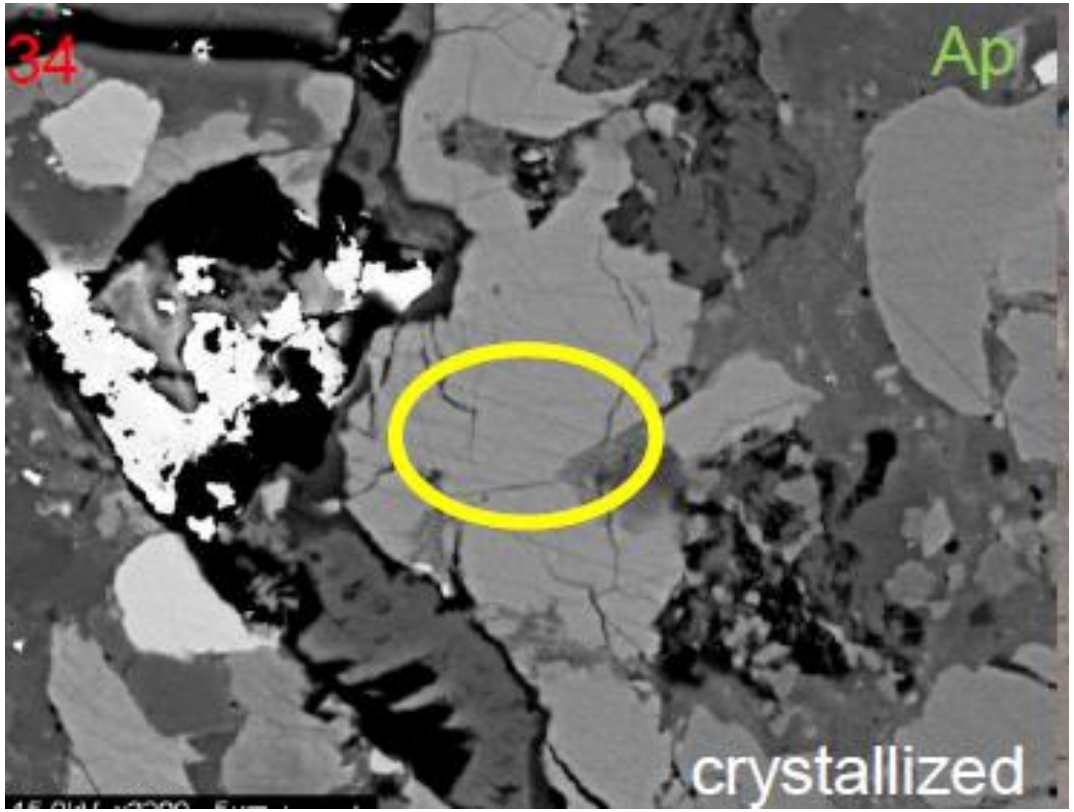

Close up of area in yellow square at right.
SIMS spot location shown as yellow oval.

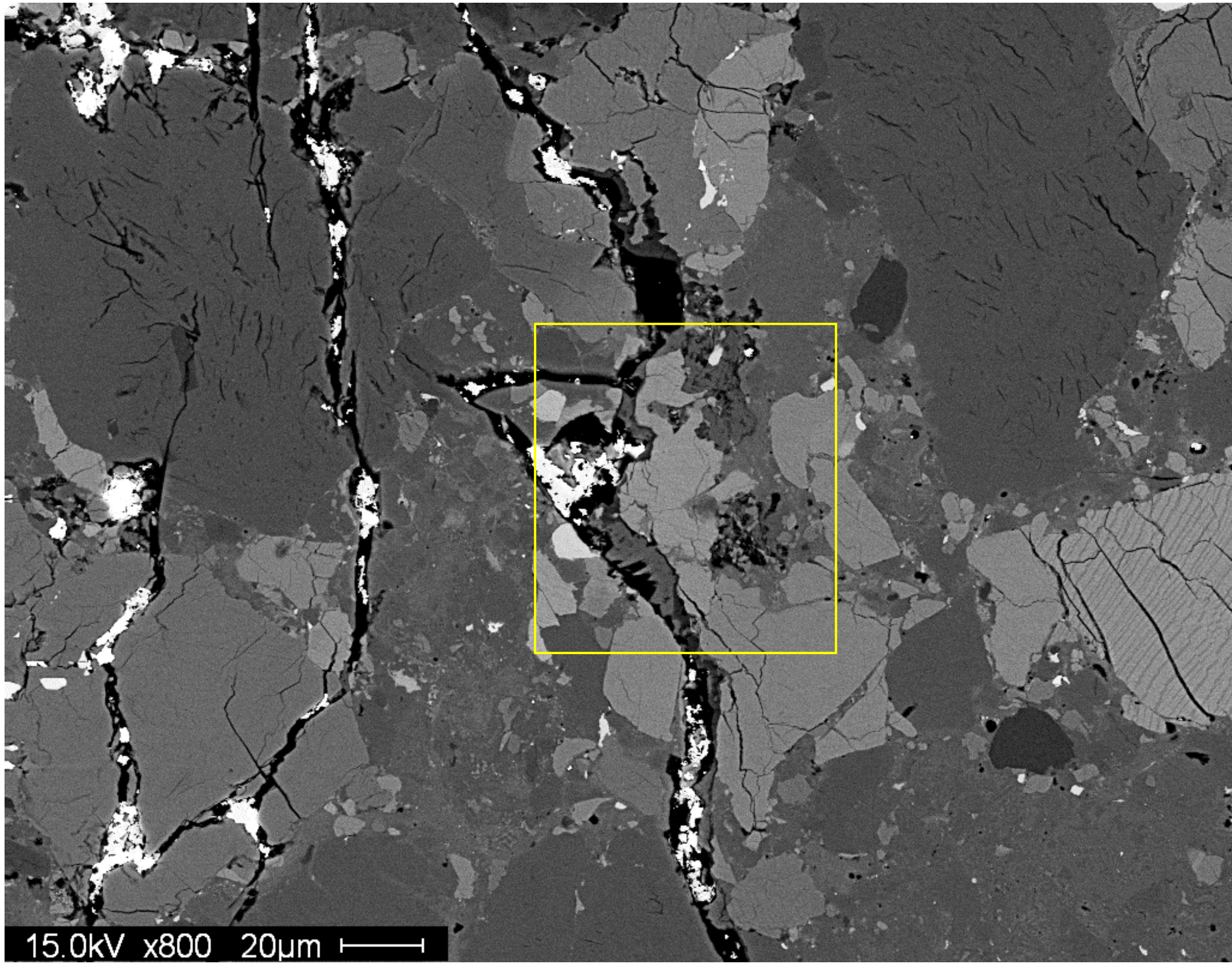

Supplementary Figure 12n. Apatite Grain #39 – not on isochron

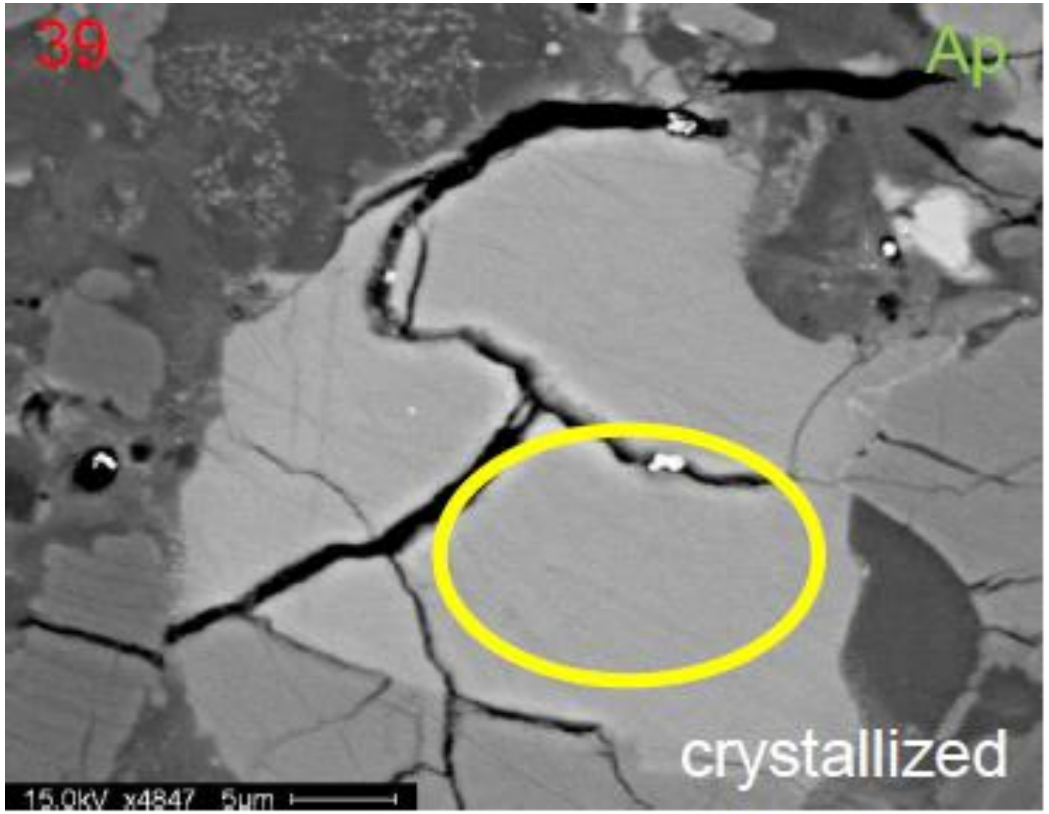

Close up of area in yellow square at right.
SIMS spot location shown as yellow oval.

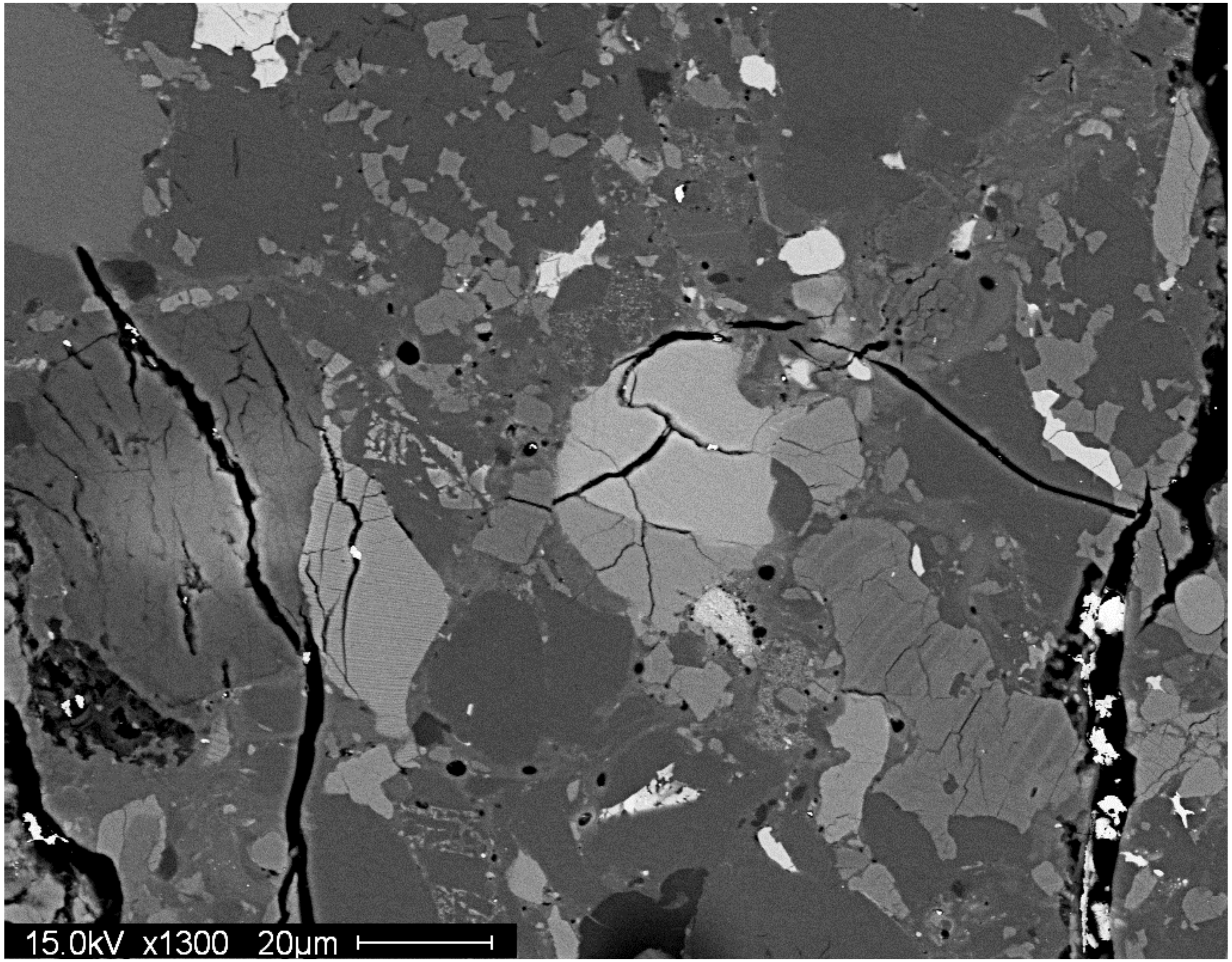

Supplementary Figure 13 (a-l). SIMS Dating of NWA 2995 showing BSE images and cathodoluminescence images with the location of NWA 2995 Zircon $^{207}Pb/^{206}Pb$ and U-Pb points collected in Beijing (red circles labelled B: Supplementary Table 11) and Stockholm (blue circles labelled S : Supplementary Table 12).

Supplementary Fig. 13a. Zircon 1

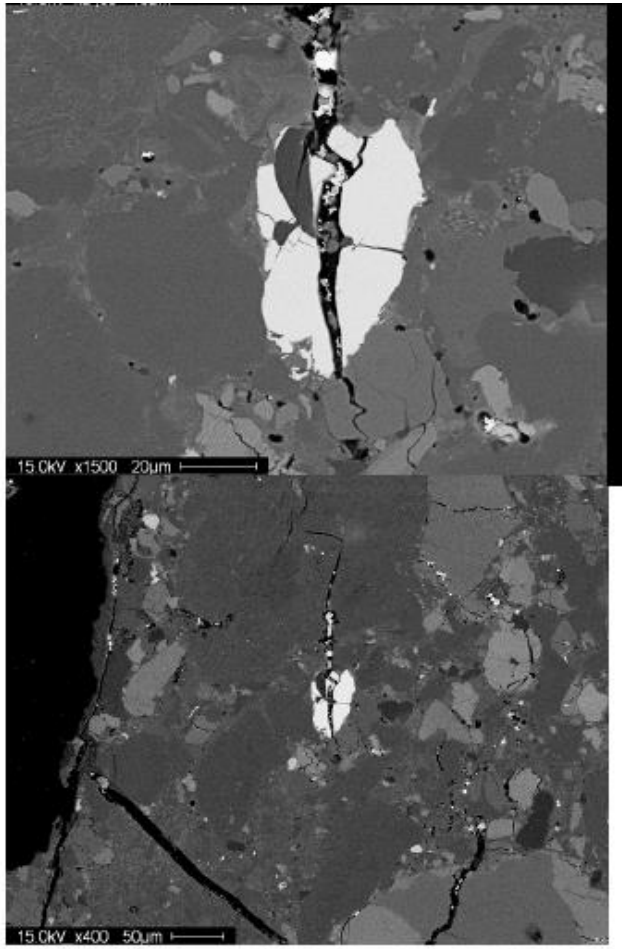

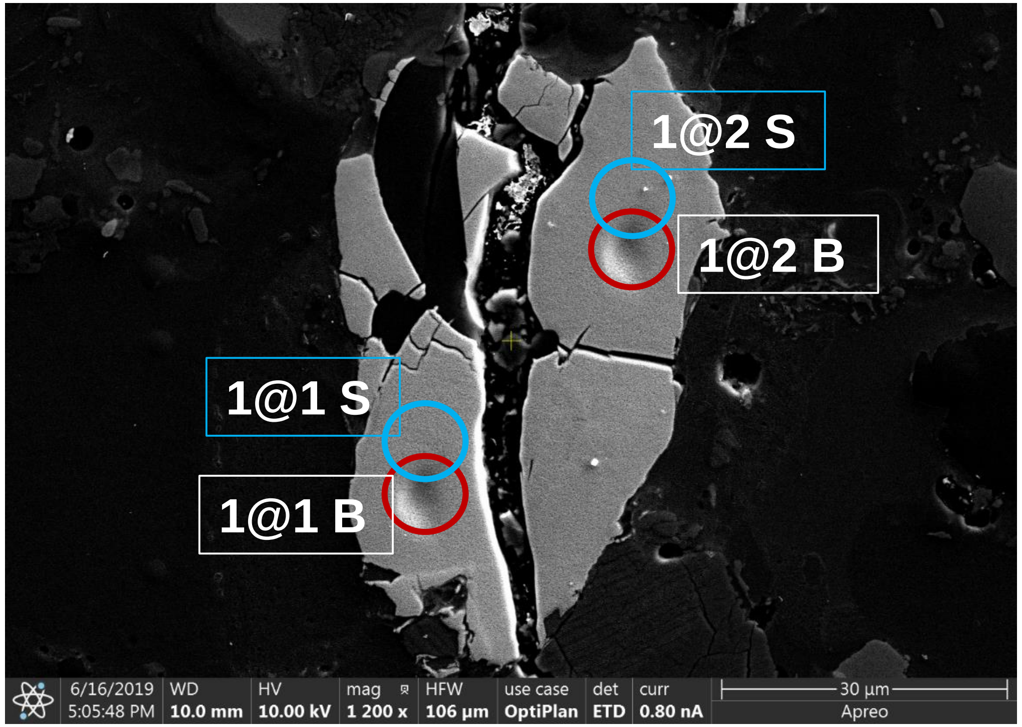

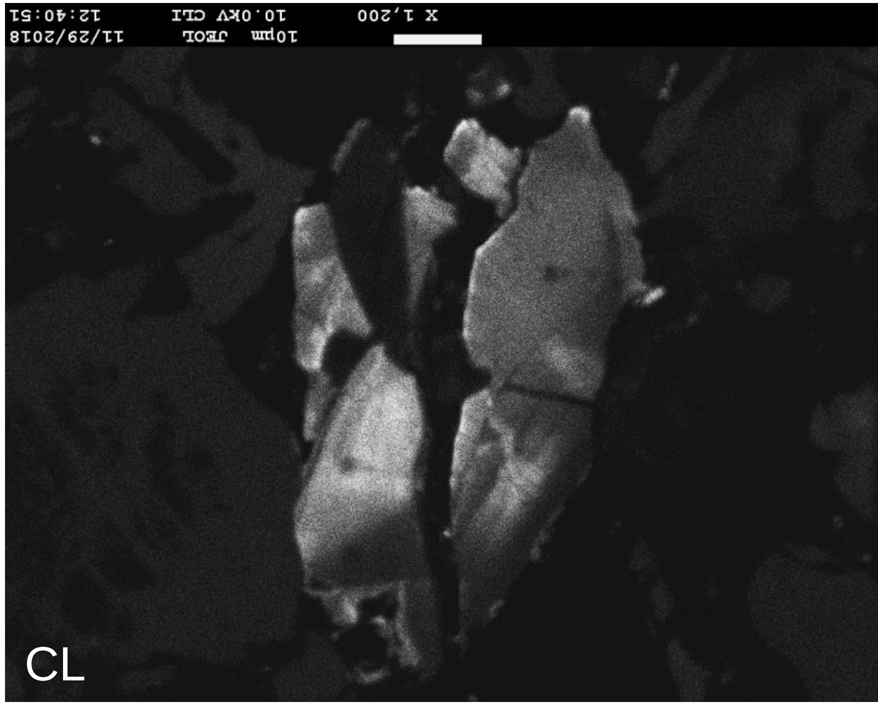

Grain in matrix attached to a Ca-bearing pyroxene grain at base, contains two Si-rich inclusions and has a large crack running through centre. Variable CL activeness. RAMAN data indicate some intermediate grain damage with broadened peaks.

SIMS

- Beijing: was analyzed with two spots, but Grain 1 is close to the edge of the sample - the Pb count is very low, and the uncertainties are very large. Data are rejected from analysis and are not reported.
- Stockholm: was analyzed with two spots, again errors are large on measurement and are not concordant so not on isochron.

Supplementary Fig. 13b. Zircon 15

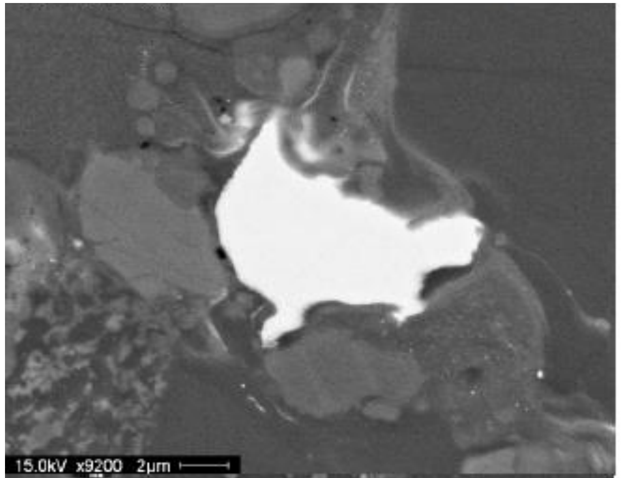

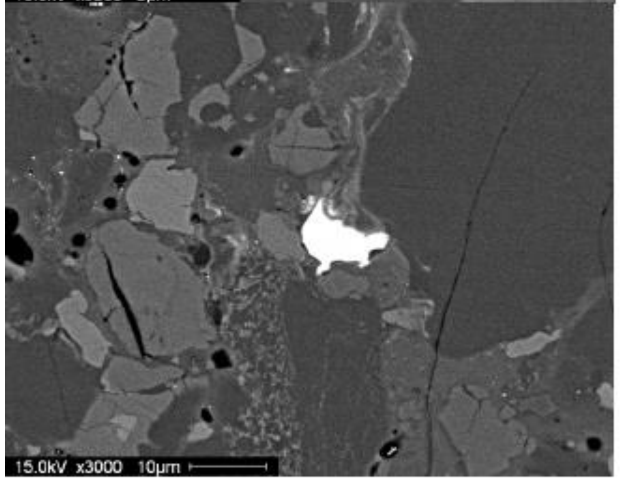

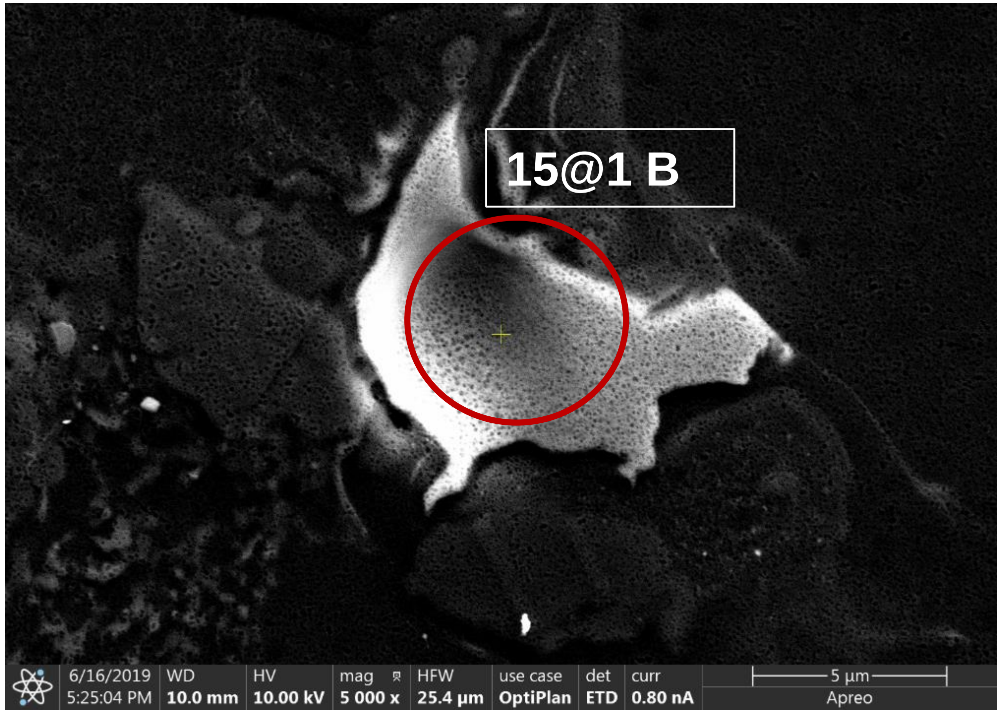

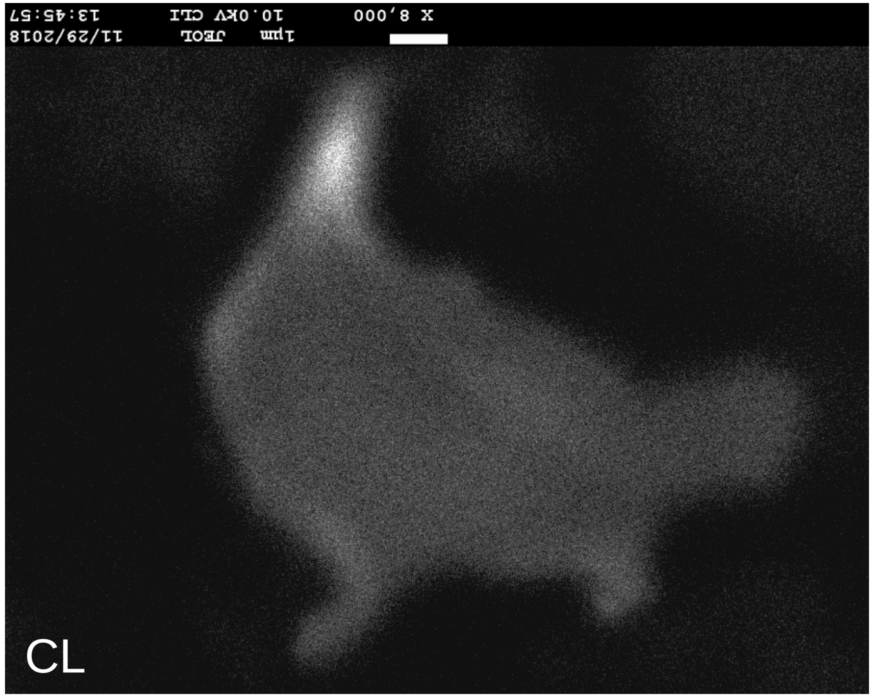

Small in matrix attached appears to have a contact with a melted area and margins looks like they have had some melting. CL activeness is unclear. RAMAN data indicate some intermediate grain damage with broadened peaks.

SIMS

- Beijing: was analyzed with one spot.
- Stockholm: not analysed.

Supplementary Fig. 13c. Zircon 20

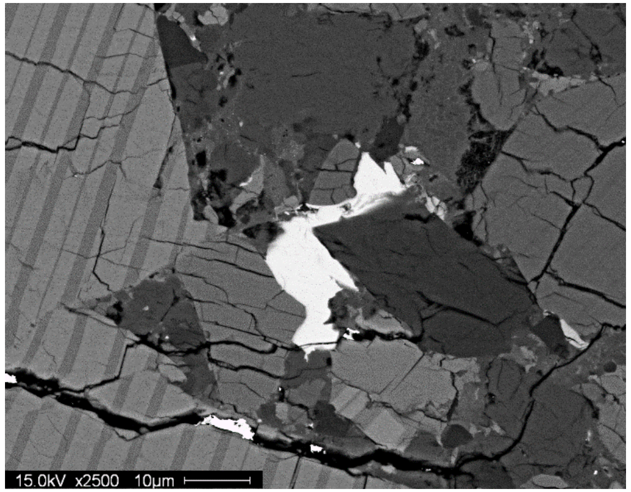

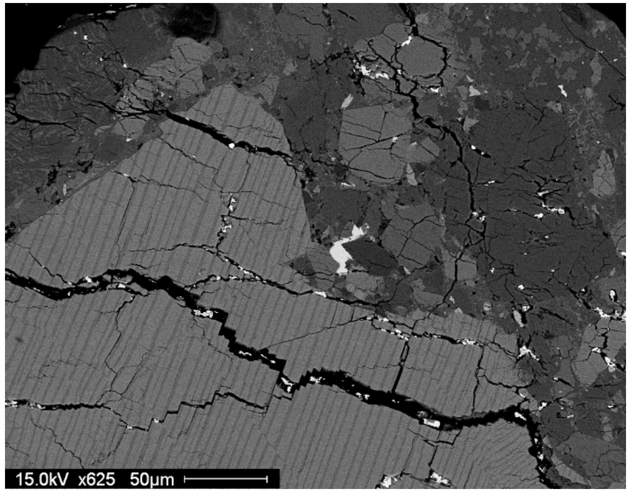

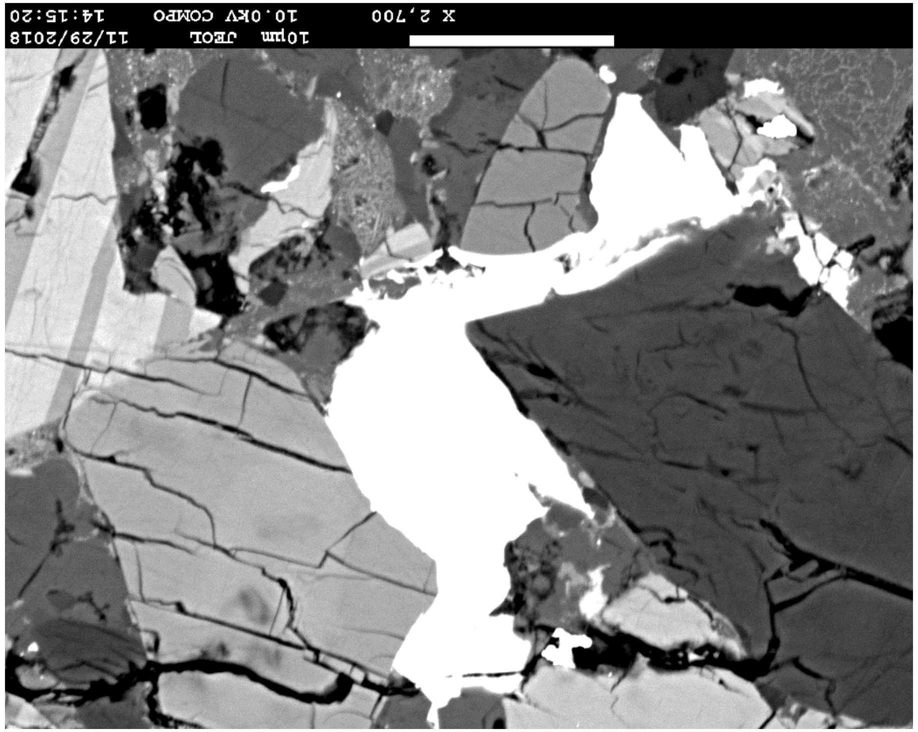

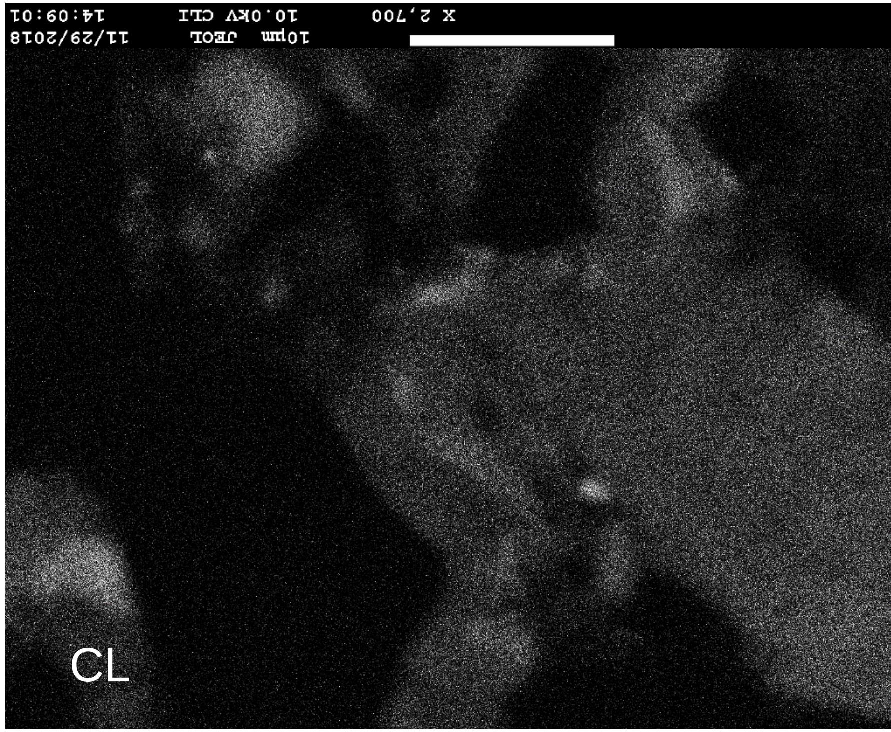

Zircon associated with QMG Clast 2 – it is likely part of this assemblage. CL activeness is unclear. No RAMAN data.

SIMS

- Beijing: not analysed.
- Stockholm: not analysed.

Supplementary Fig. 13d. Zircon 21

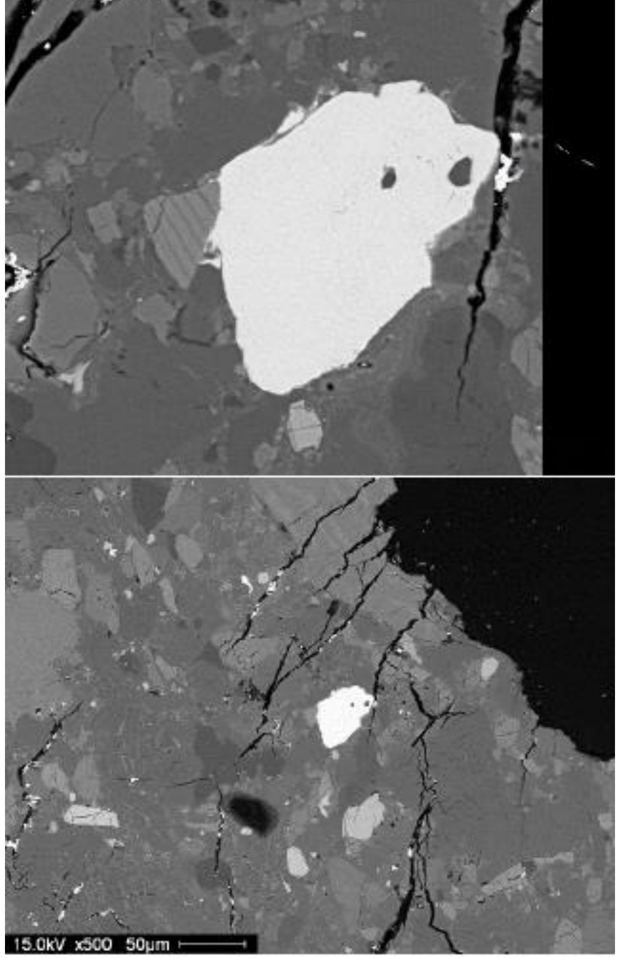

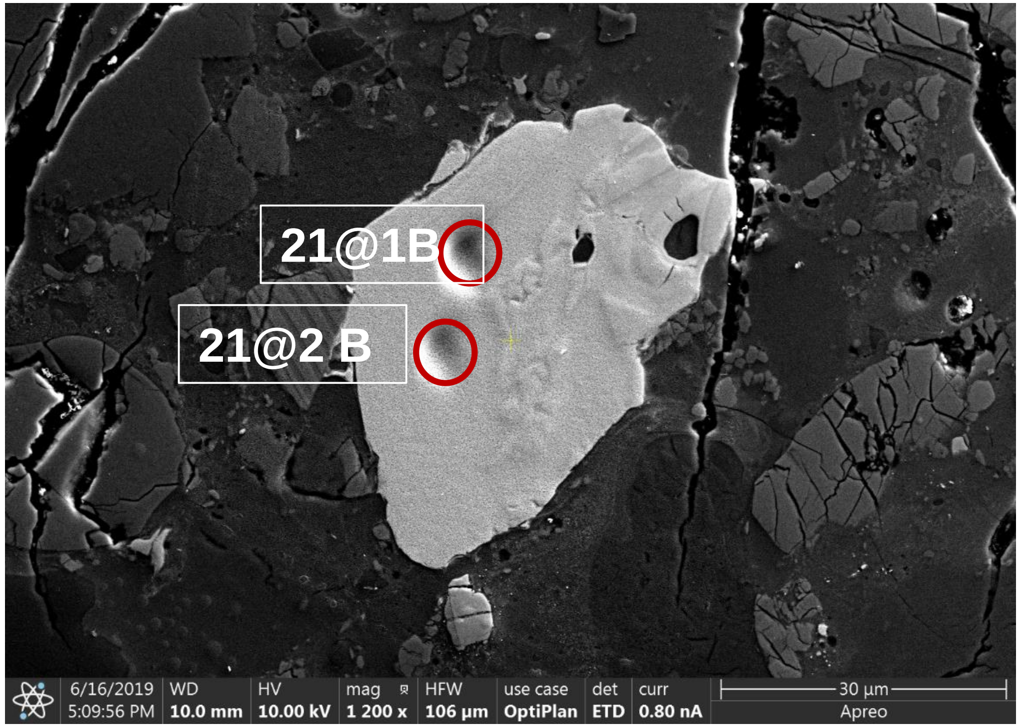

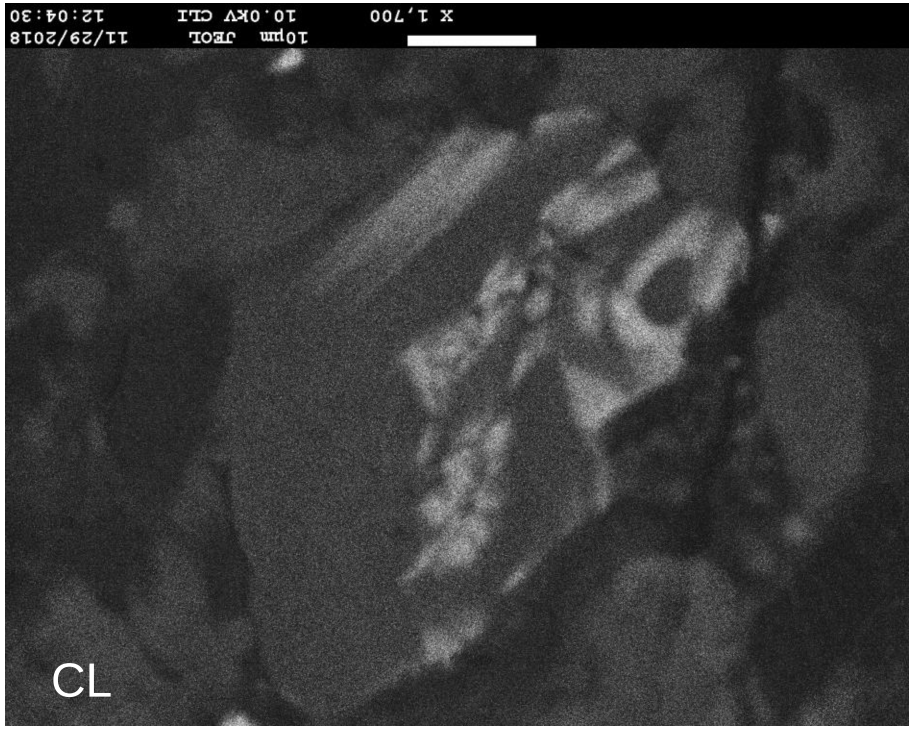

Grain in matrix attached to an exsolved pyroxene, contains two Si-rich inclusions. Variable Cl activeness with some CL active and CL inactive zones. RAMAN data indicate some intermediate grain damage with broadened peaks in the CL active areas, and highly damaged zones associated with the CL inactive area.

SIMS

- Beijing: was analyzed with two spots. @2 resulted in data with a large error which is excluded from the weighted analysis.
- Stockholm: not analysed.

Supplementary Fig. 13e. Zircon 35

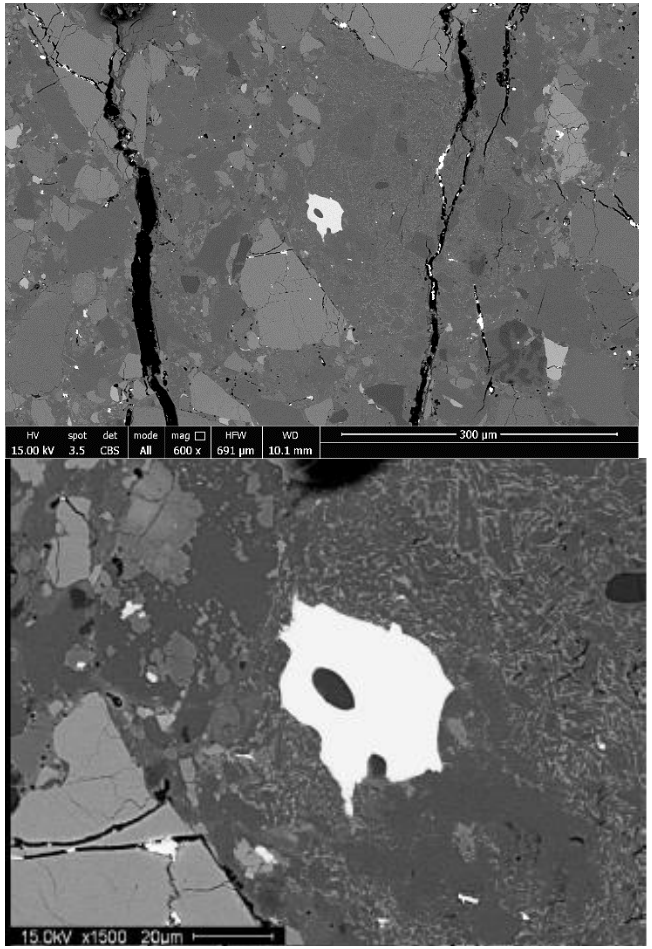

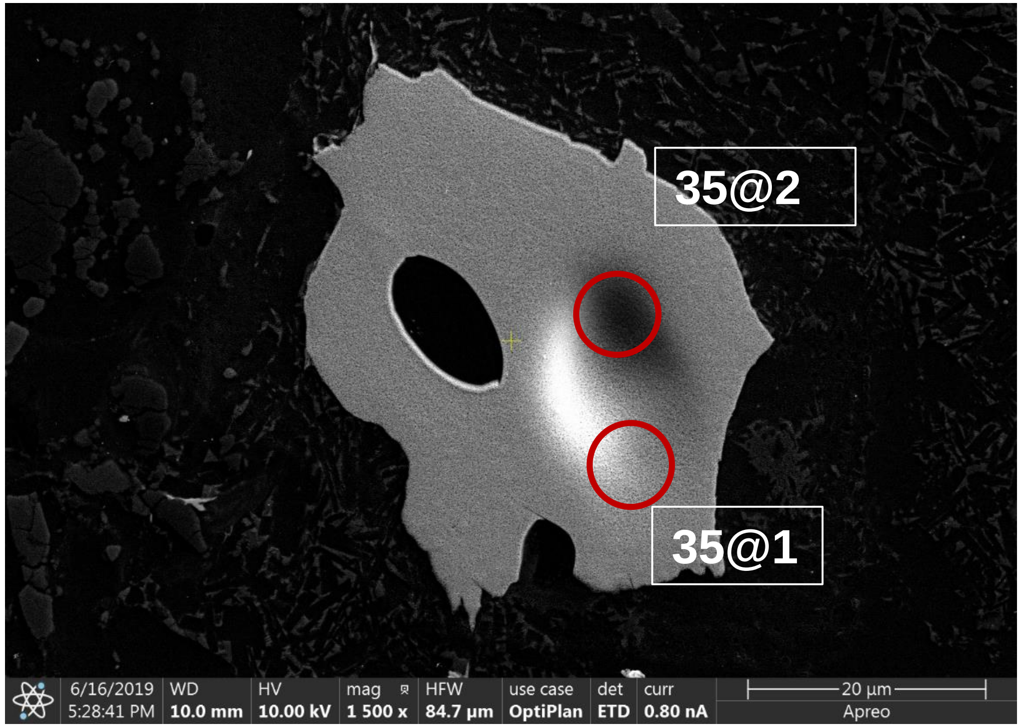

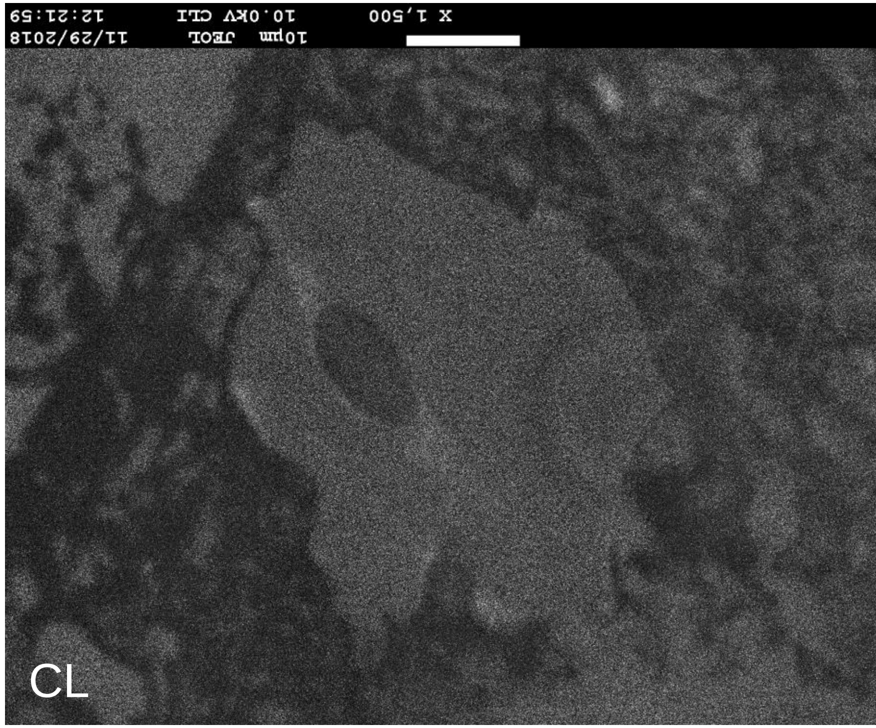

Grain within clast-rich impact melt clast, contains two Si-rich inclusions. CL inactive. RAMAN data indicate high levels of radiation damage.

SIMS

- Beijing: was analyzed with two spots.
- Stockholm: not analysed.

Supplementary Fig. 13f. Zircon 36

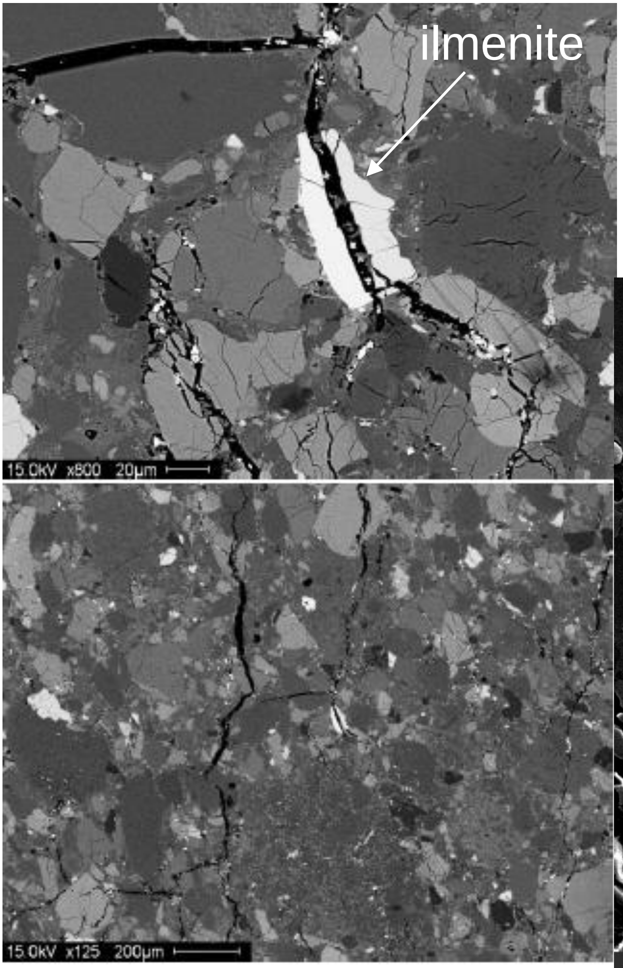

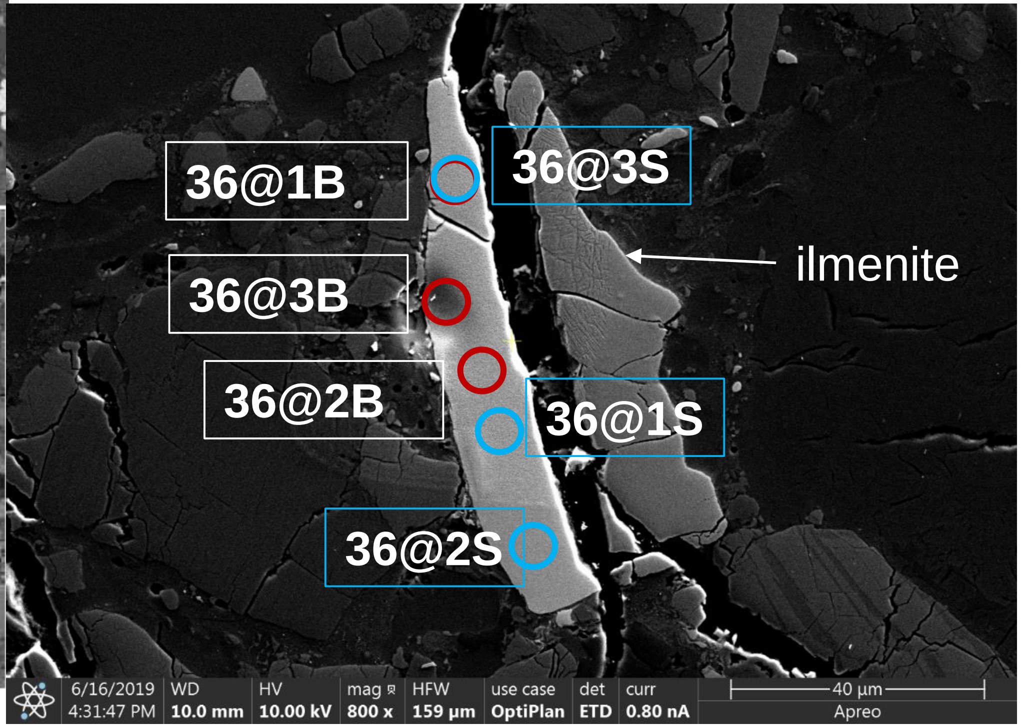

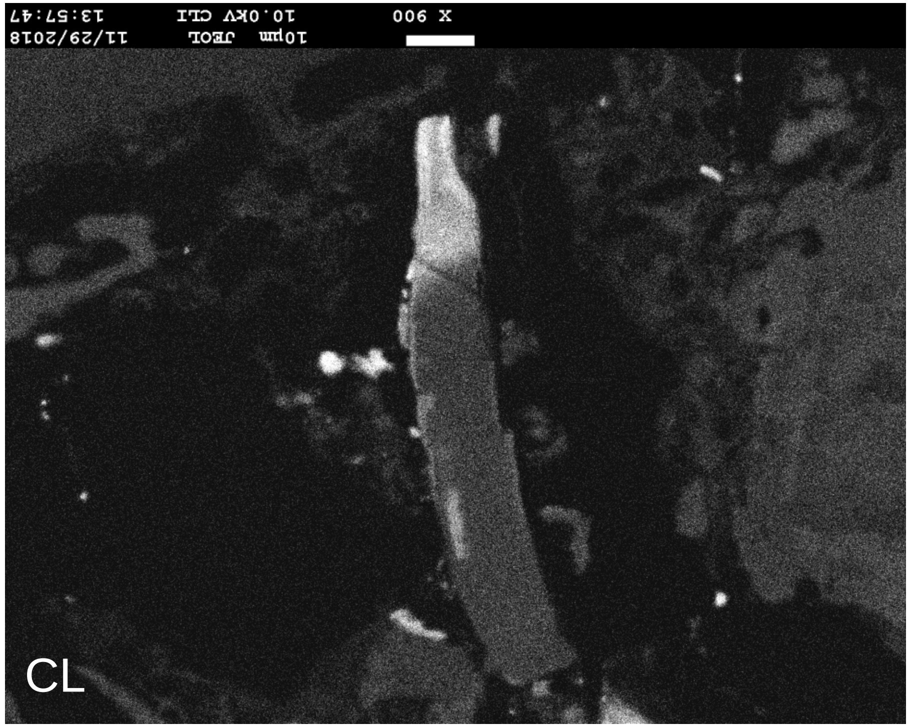

Grain with matrix, possibly associated with ilmenite grain. CL active (top) and inactive (bottom) areas. RAMAN data from CL active area has intermediate modified spectra, and in CL in active areas indicates high levels of radiation damage.

SIMS
- Beijing: was analyzed with three spots.
- Stockholm: analysed with three spots.

Supplementary Fig. 13g Zircon 41

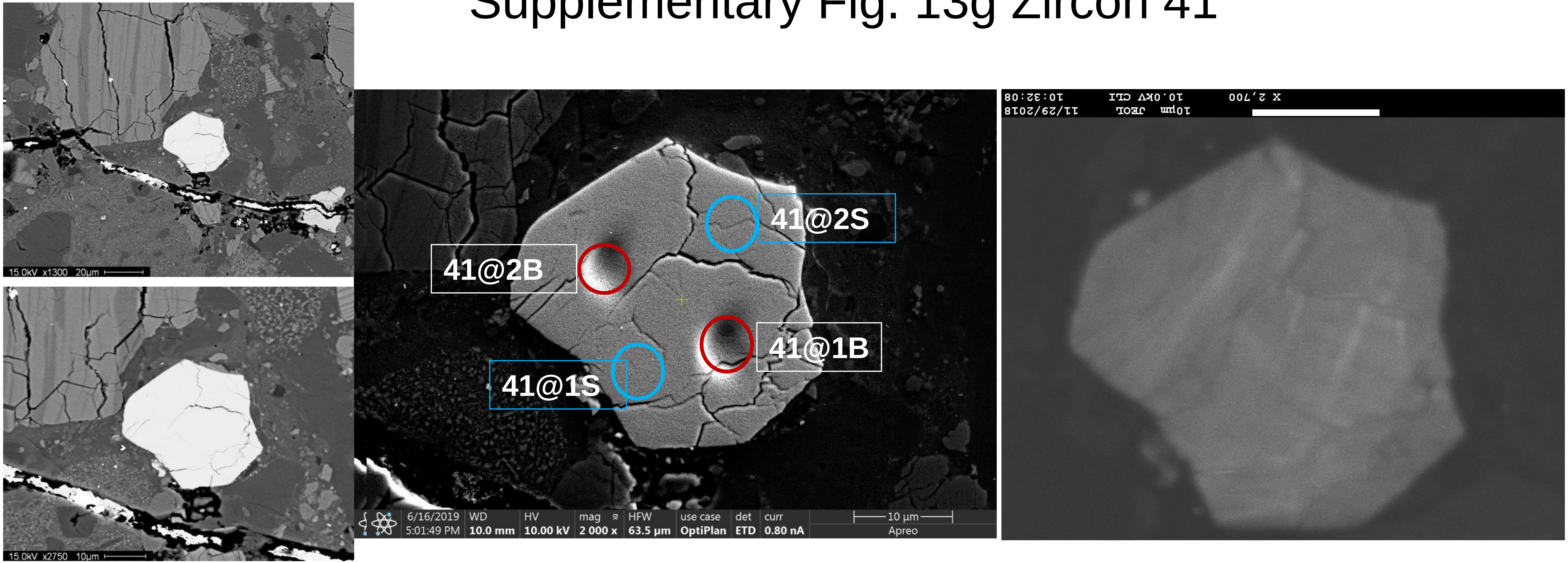

Grain with matrix. CL active. RAMAN data from CL active area is relatively unmodified zircon i.e., grain has minor radiation damage.

SIMS

- Beijing: was analyzed with two spots.
- Stockholm: analysed with two spots.

Supplementary Fig. 13h Zircon 43

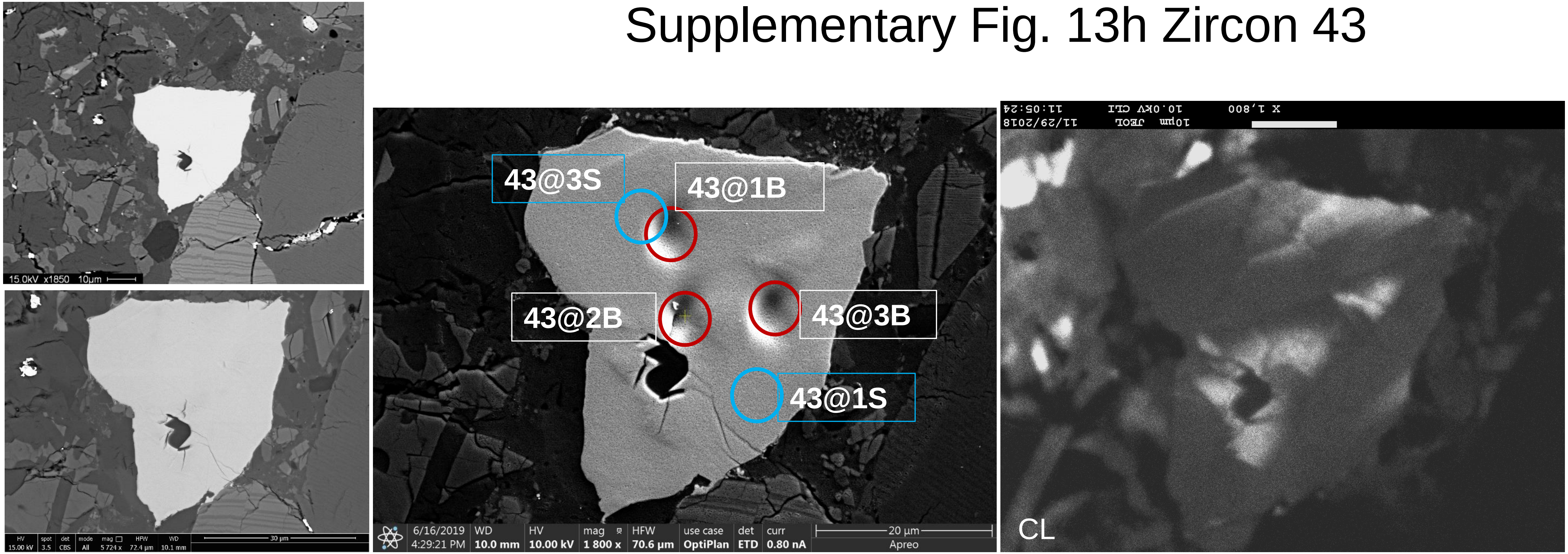

Grain attached to an exsolved pyroxene, contains a Si-rich inclusion. Variable Cl activeness with some CL active and CL inactive zones. RAMAN data indicate some intermediate grain damage in CL active areas, and high damage in CL inactive areas.

SIMS

- Beijing: was analyzed with three spots.
- Stockholm: was analyzed with three spots.

Supplementary Fig. 13i Zircon 44

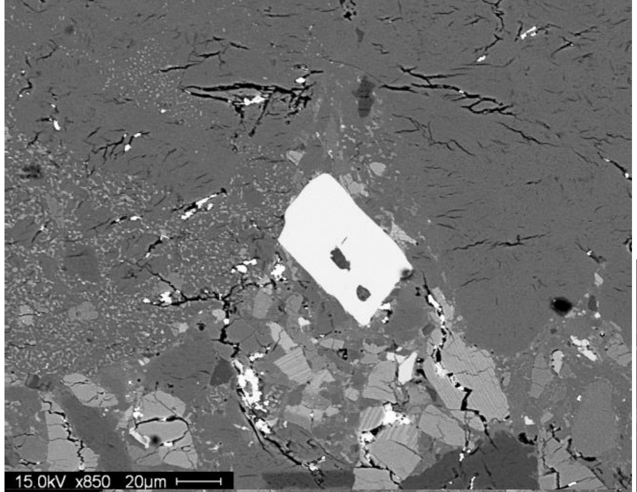

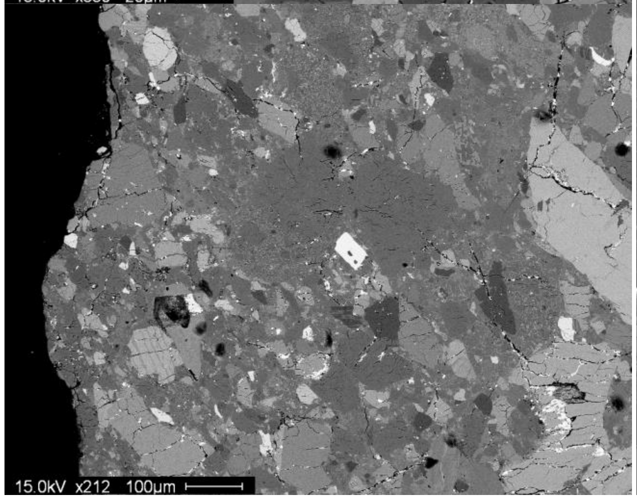

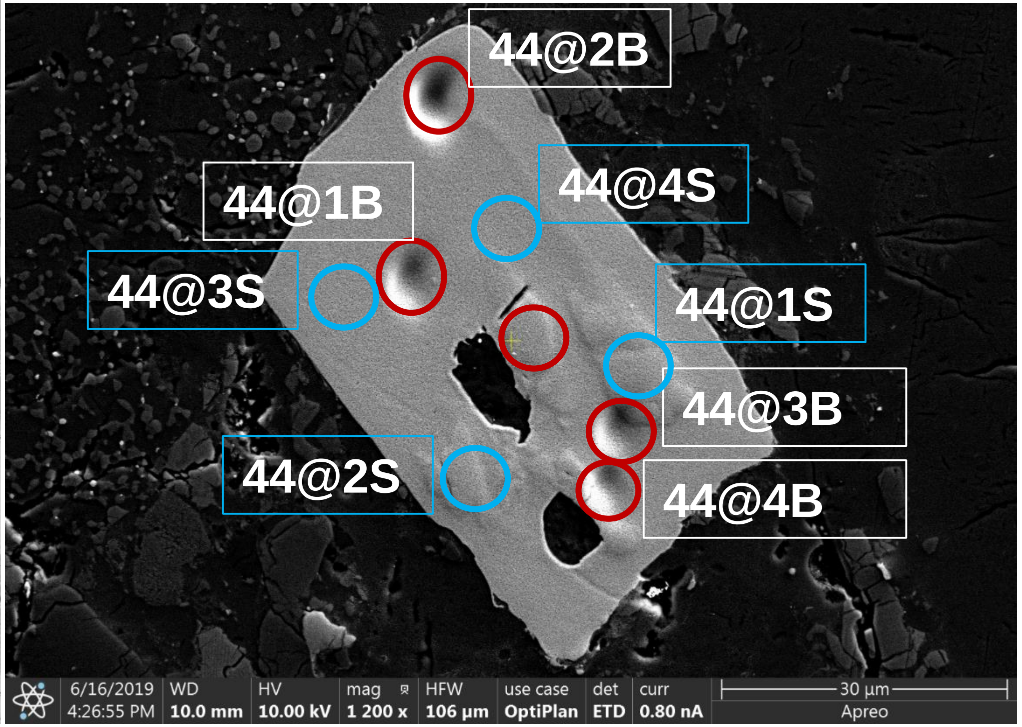

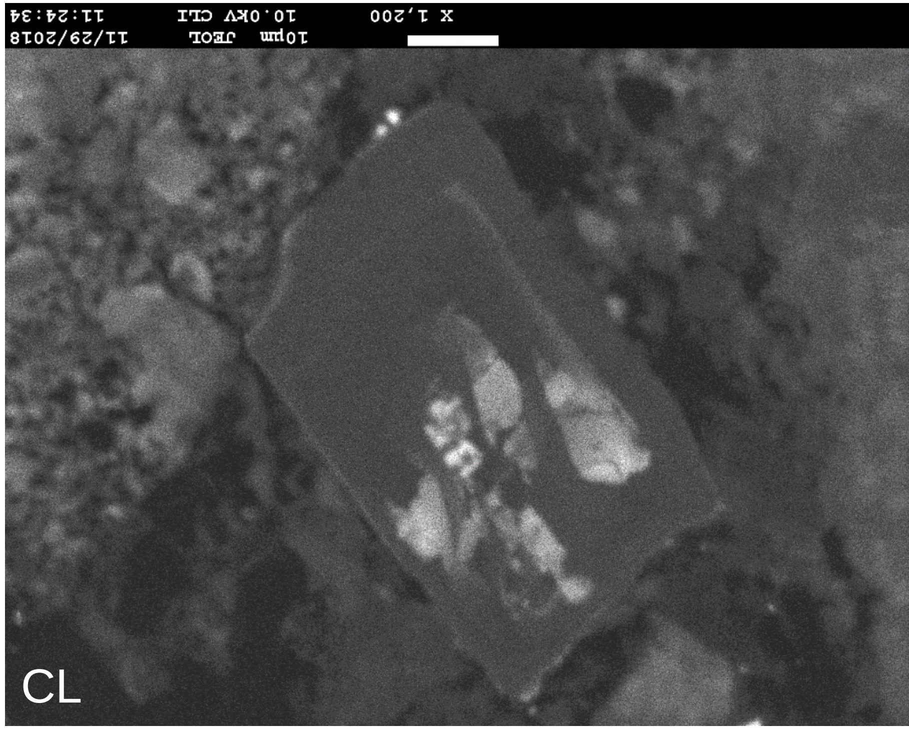

Grain in matrix, contains two Si-rich inclusions. Variable Cl activeness with some CL active and CL inactive zones. RAMAN data indicate some intermediate grain damage in CL active areas, and high damage in CL inactive areas.

SIMS

- Beijing: was analyzed with four spots, however, three give very large errors and are not included in sample weighted mean.
- Stockholm: was analyzed with four spots. Spot 44@4S is non-conordant and is excluded from isochron.

Supplementary Fig. 13j Zircon 62

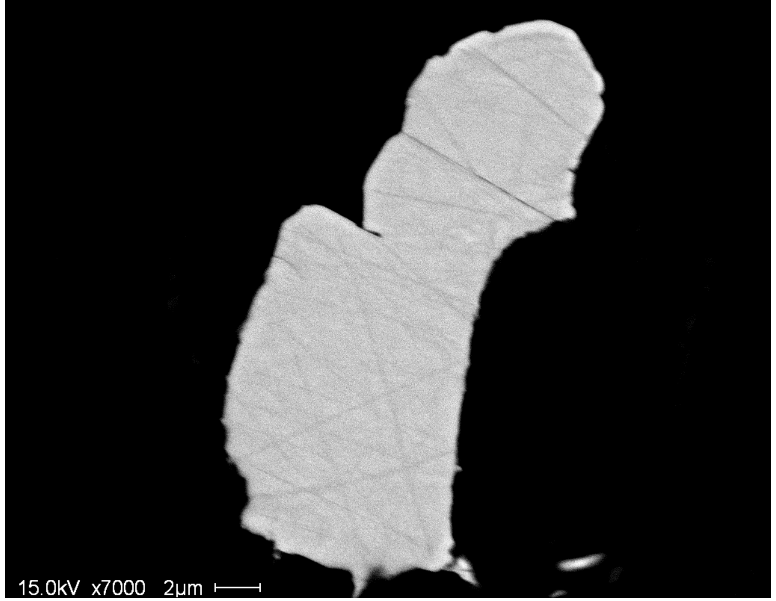

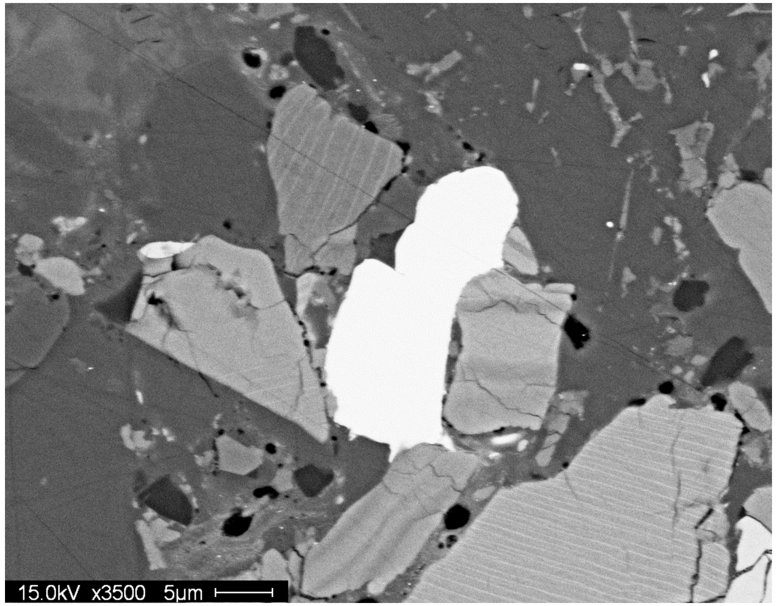

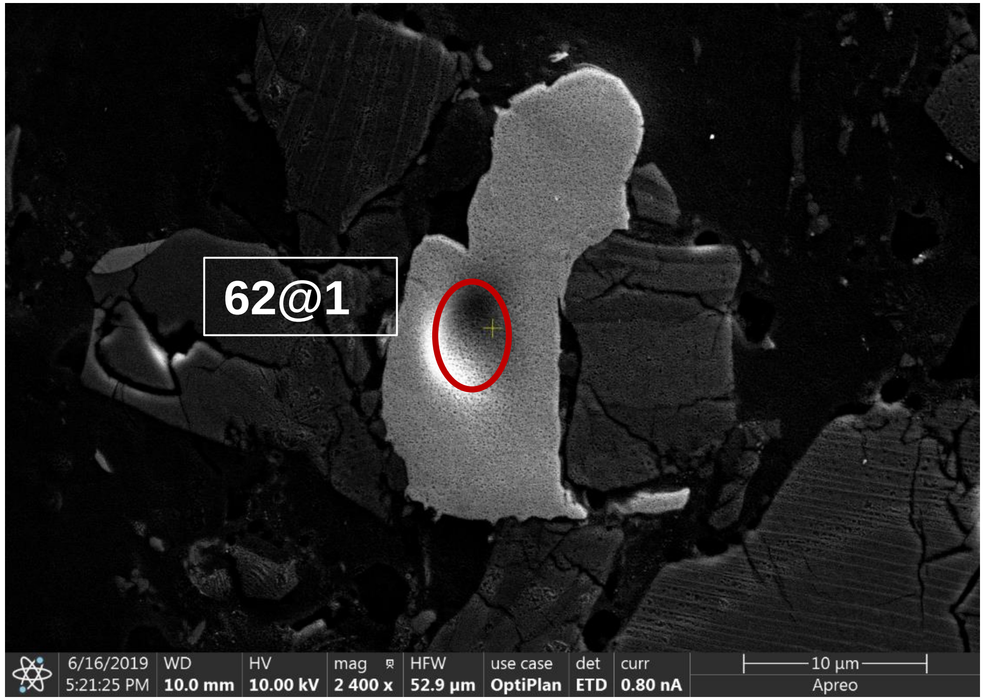

Grain attached to an exsolved pyroxene. No CL image. RAMAN data indicate some intermediate grain damage.

SIMS

- Beijing: was analyzed with one spot.
- Stockholm: was not analyzed.

Supplementary Fig. 13k Zircon 63

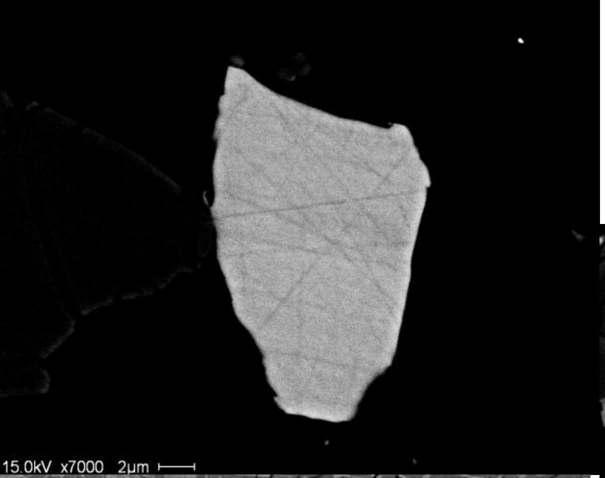

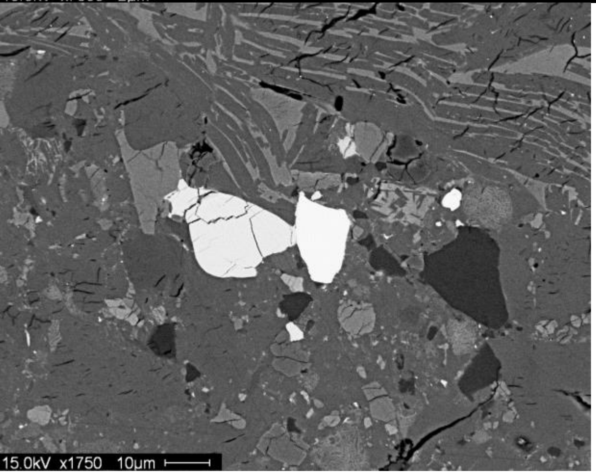

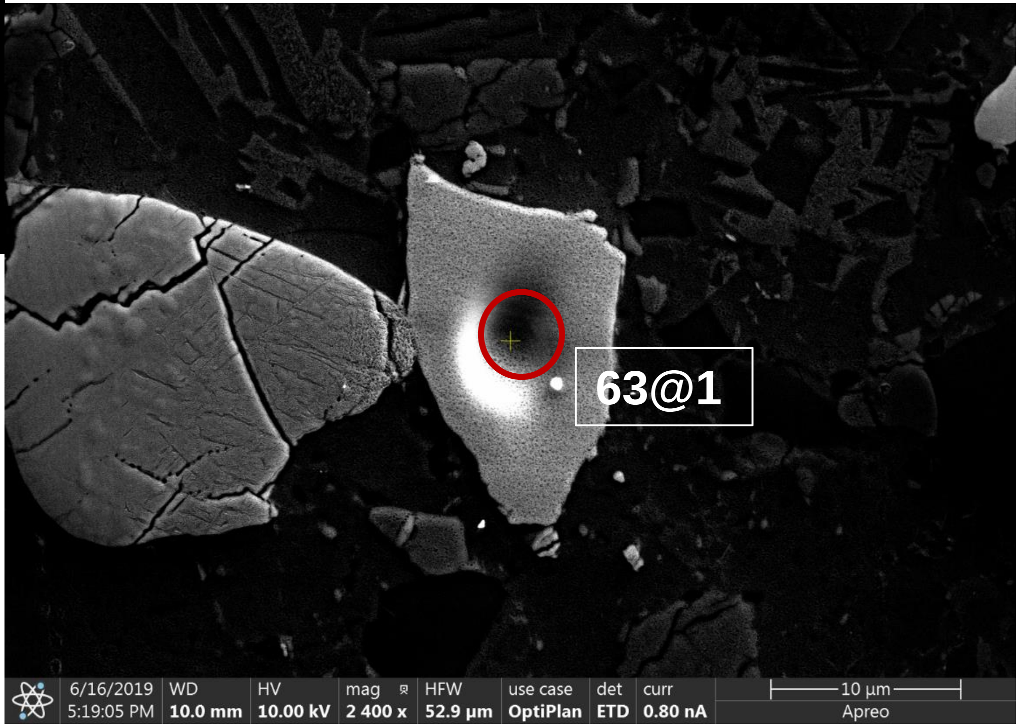

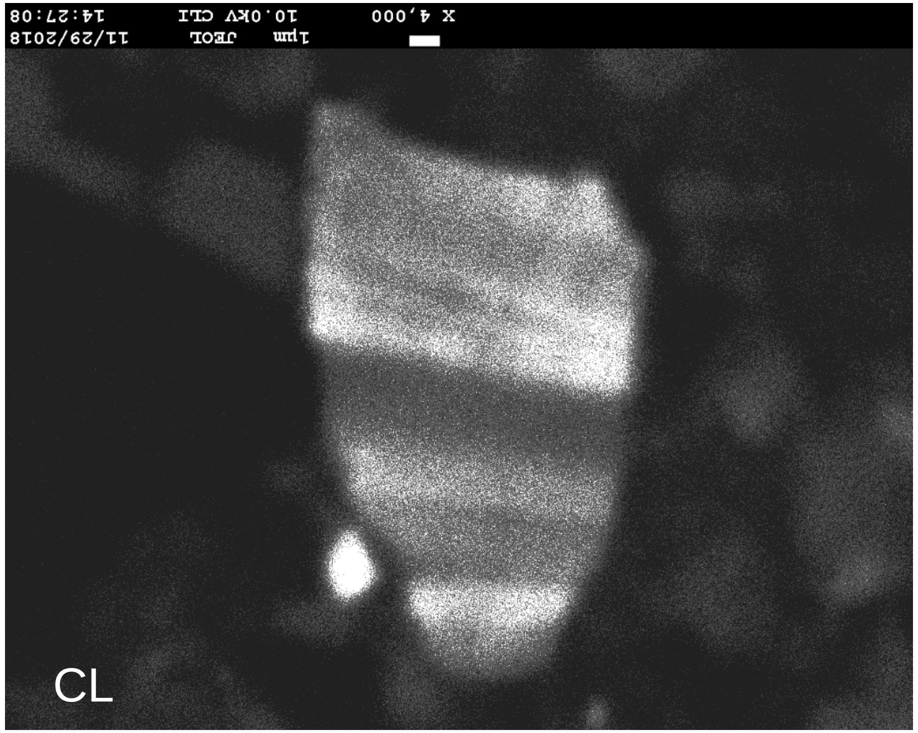

Grain in matrix. Variable Cl activeness with some CL active and CL inactive zones. RAMAN data indicate some intermediate grain damage in CL active areas.

SIMS

- Beijing: was analyzed with one spot.
- Stockholm: was not analyzed.

Supplementary Fig. 13l. Other zircon in NWA 2995 that were not dated by SIMS

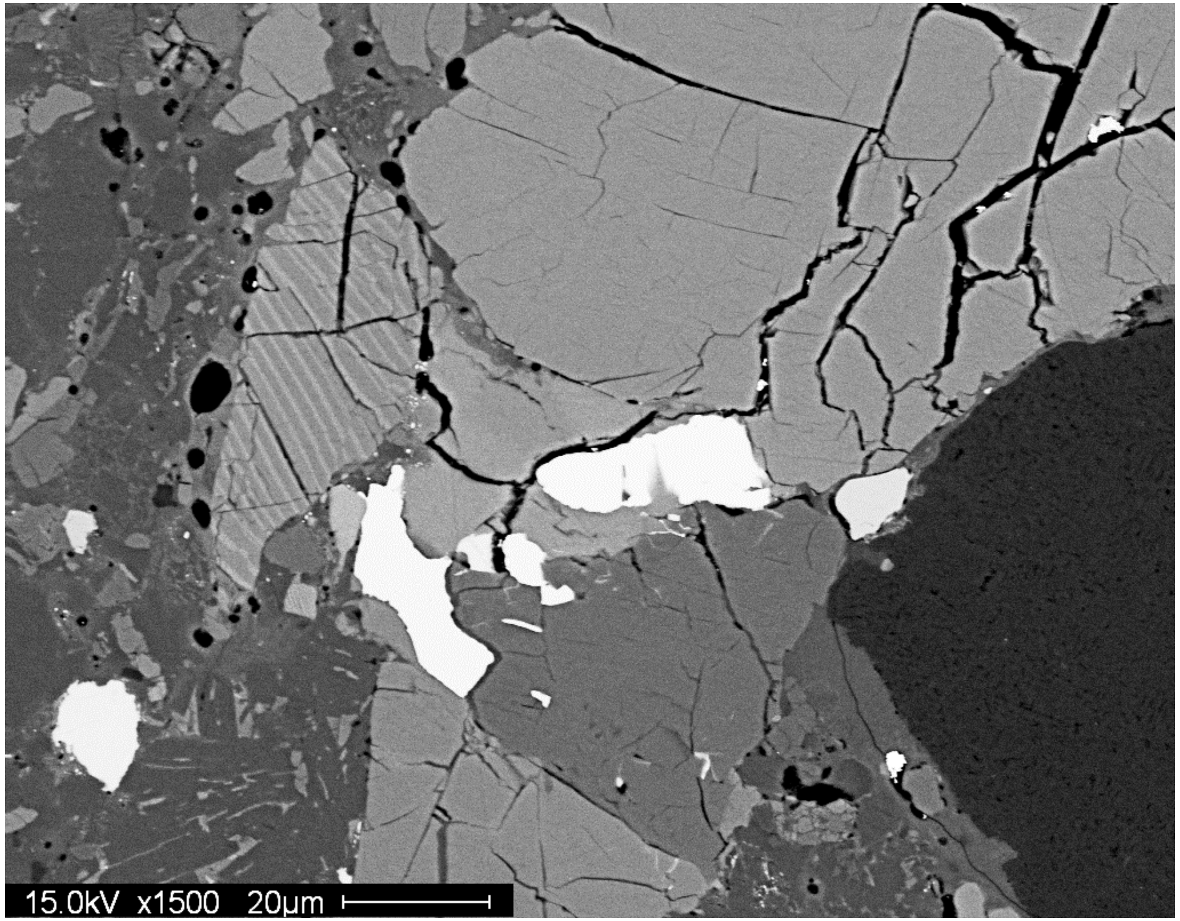

#48 within QMG

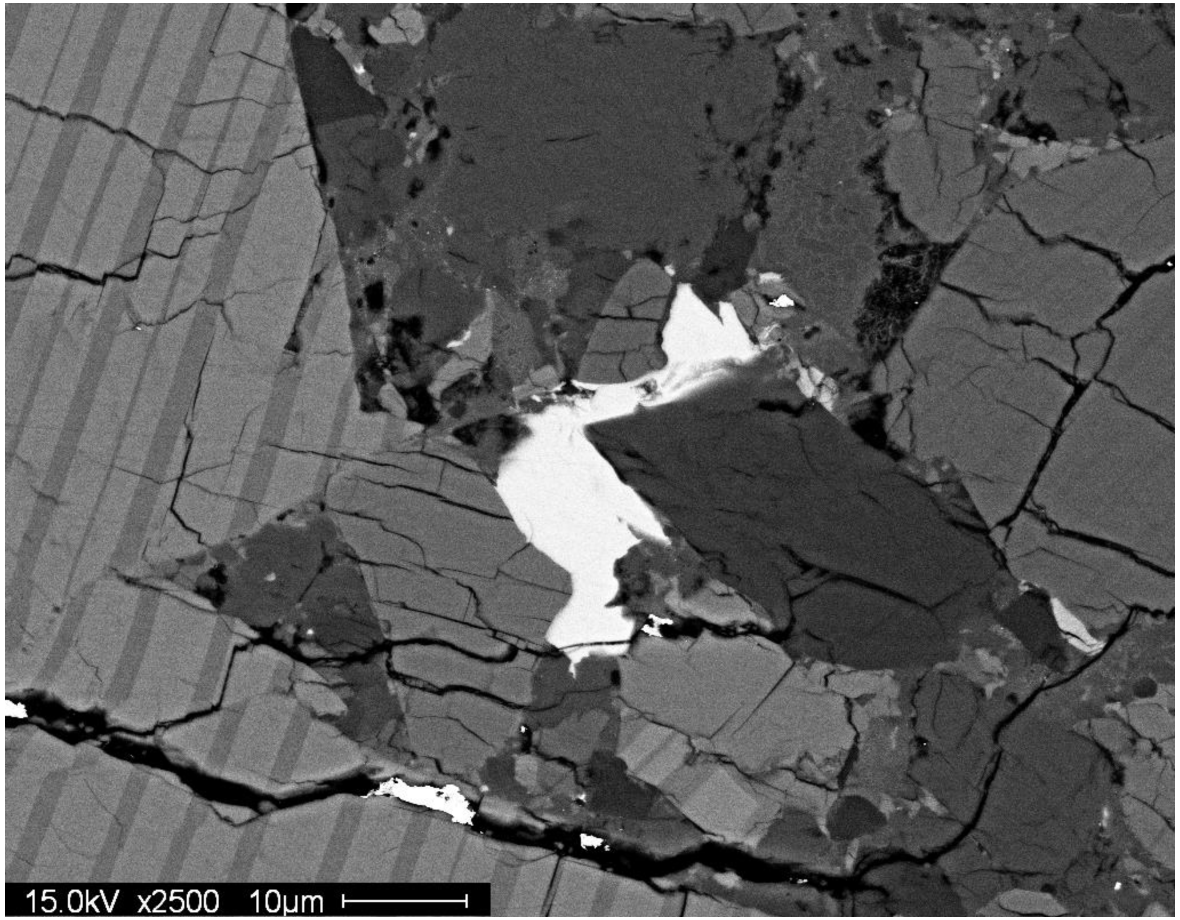

#20 within Clast 2 QMG clast

Supplementary Figure 14 (a-d). BSE images of clasts dated by SIMS Pb-isotope in Stockholm (data in Supplementary Table 13).

Supplementary Figure 14a
Granitic Clast 4

Sufficient data from within this clast for an internal isochron

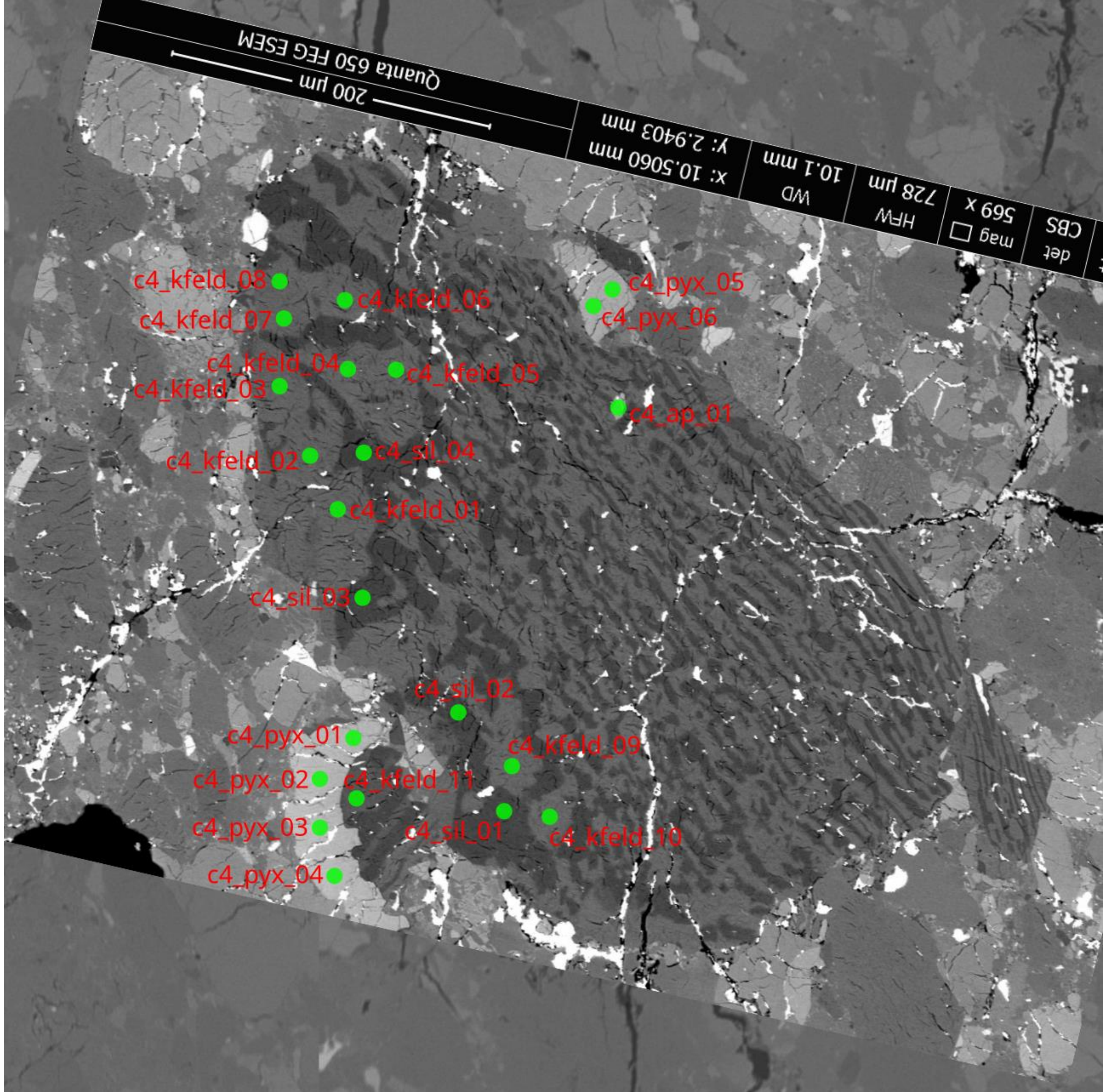

Supplementary Figure 14b
Granitic Clast 3

Insufficient data from within this clast for an internal isochron (because of either low counts or terrestrial contamination)

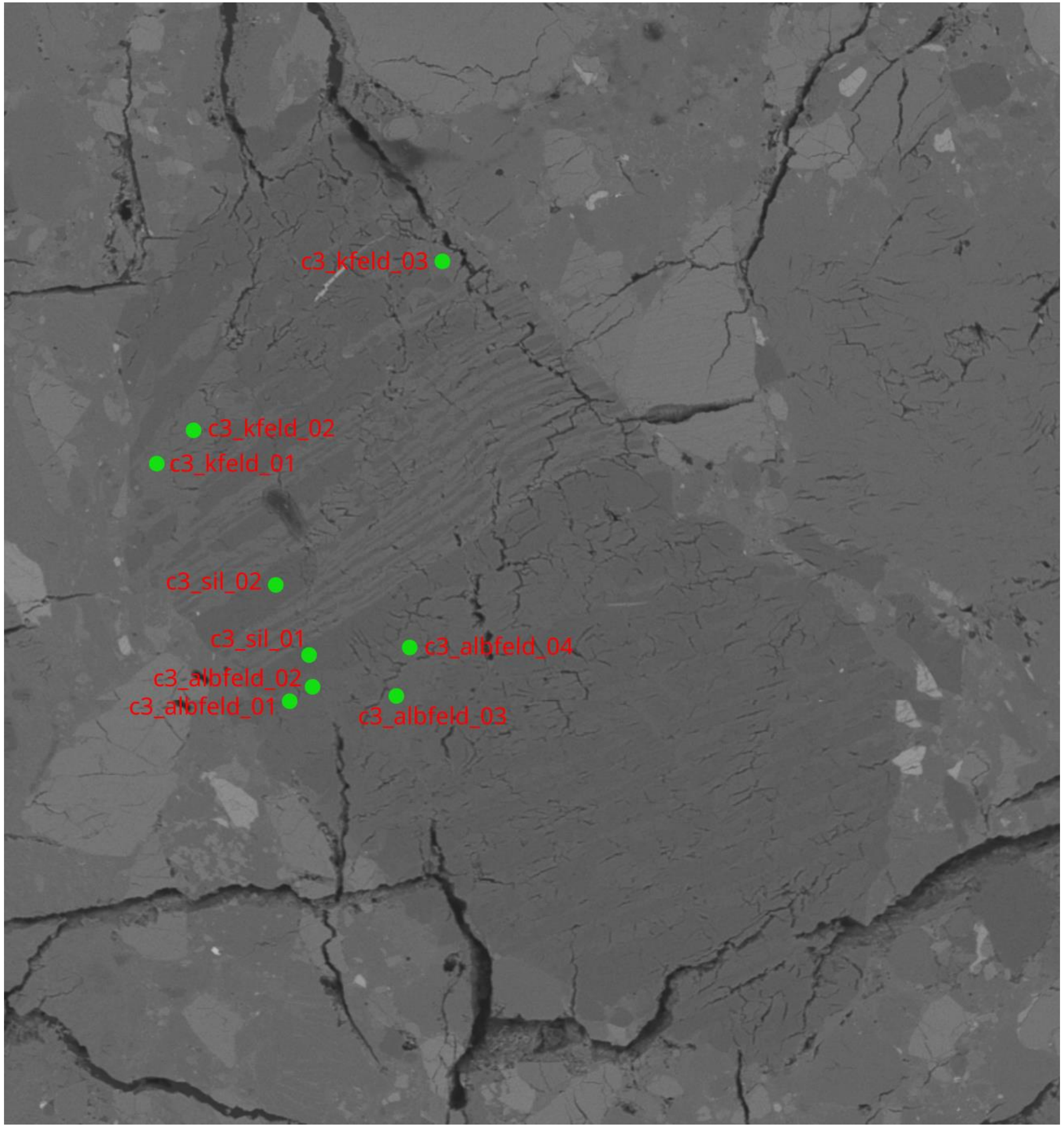

Supplementary Figure 14c. Granitic Clast 5

Named 4b in spot naming protocol

Insufficient data from within this clast for an internal isochron (because of either low counts or terrestrial contamination)

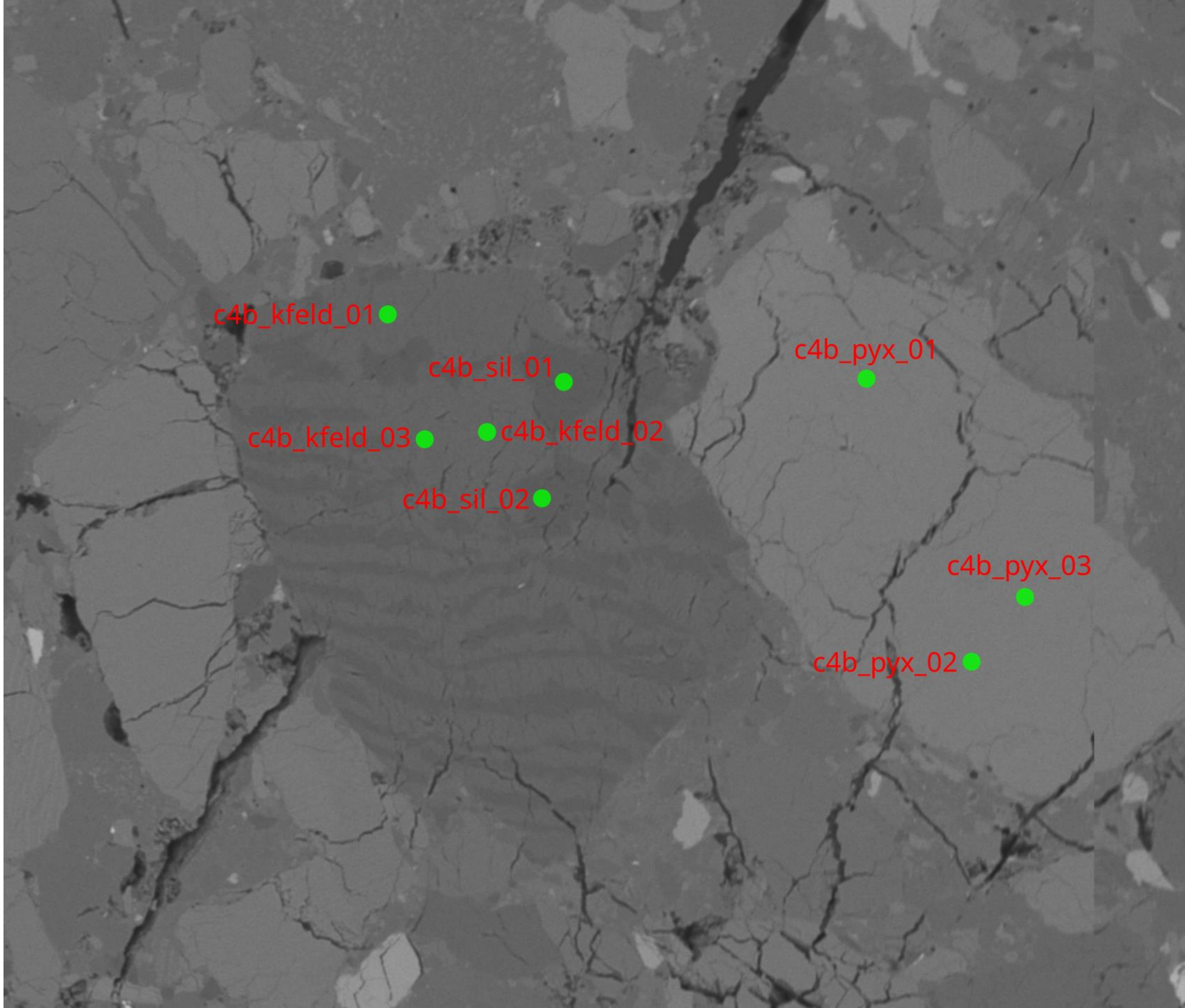

Supplementary Figure 14d. Granitic Clast 8

Insufficient data from within this clast for an internal isochron (because of either low counts or terrestrial contamination)

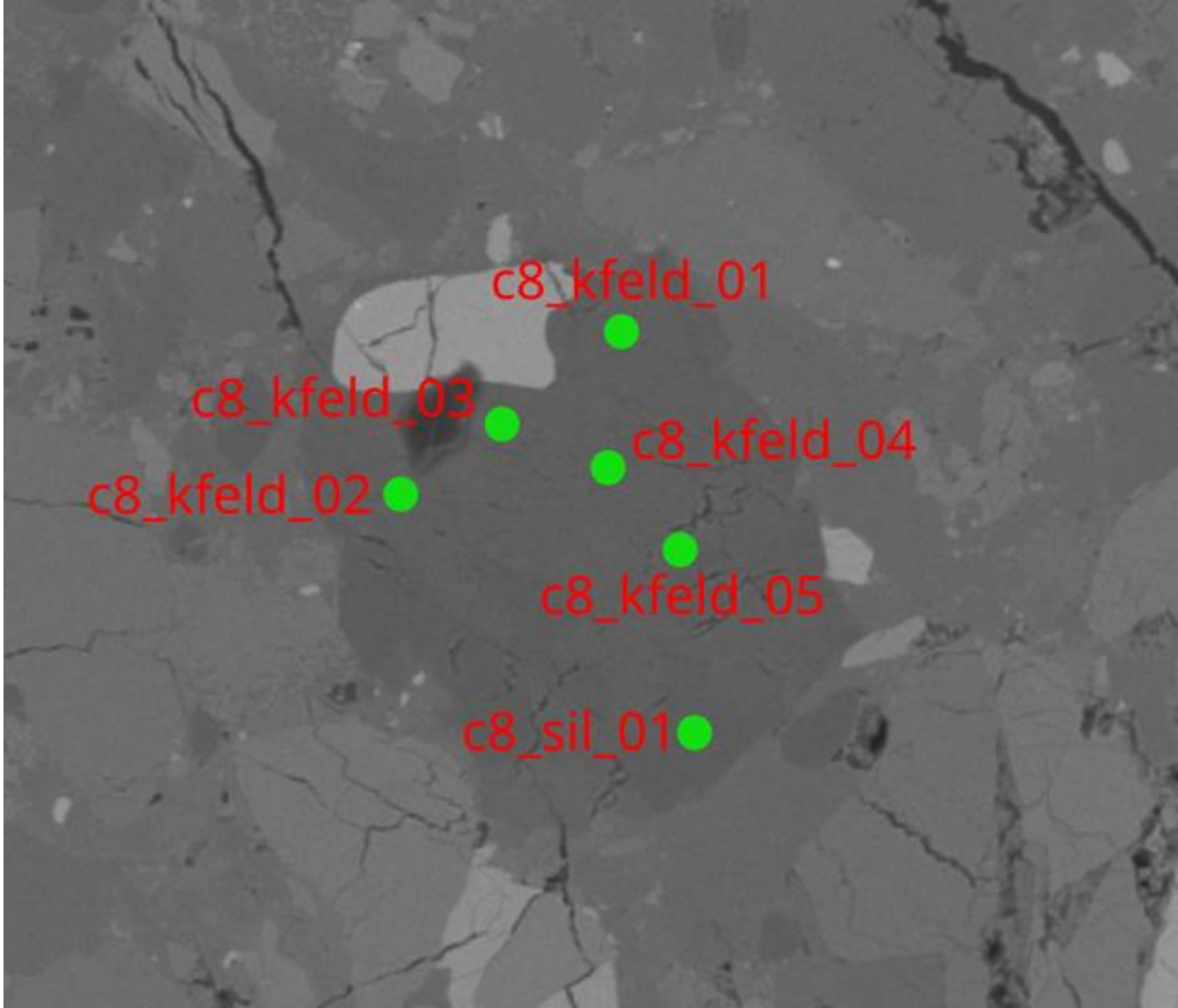

Supplementary Note 1 – Meteorite Provenance Analysis

The final results of the NWA 2995 meteorite provenance analysis are shown in Figure 4 of the main paper with the data listed in Supplementary Tables 15 and 16. This note contains details of the method, validation, and results.

Data and Method Validation

Here we develop the approach first outlined in *Joy et al. (2010)*, later used *by Joy et al. (2011, 2014)* and *Mercer et al. (2013)*, and explored in detail by *Calzada-Diaz et al. (2015)* to use the Lunar Prospector gamma-ray spectrometer (LP-GRS) global orbital chemistry dataset to investigate the potential source region(s) of NWA 2995. Our new approach here improves on that used in these prior works, although does not necessarily invalidate these earlier results. Instead, it provides better understanding and confidence of the probability of likely compositional affinities of the meteorites and the lunar surface. Our new method utilises a chi-squared analysis to assess the absolute 'distance' (in terms of likelihood) between a given sample elemental abundances (and their uncertainties) and the Lunar Prospector dataset (and its reported uncertainty). By considering the full LP-GRS per pixel errors, we can use the number of elements (i.e., the degree of freedom) to generate probability indices (P-values) for each spatial datapoint (pixel) in the LP-GRS dataset. Thus, we can consider utilising as many of the available LP-GRS element datasets as we assess are appropriate to perform statistical hypothesis testing.

Lunar Prospector chemistry data: We have used the LP-GRS HIGH1 2° per pixel dataset (equivalent to 60 × 60 km square pixels) of *Prettyman et al. (2006)* for Th, K, U, FeO, TiO_2, SiO_2, MgO, CaO, and Al_2O_3 abundances (Supplementary Figure 15). Though there are higher spatially mapped versions of Lunar Prospector elemental data (*Lawrence et al., 2002, 2003; Peplowski and Lawrence, 2013*), as well as elemental compositional data reported from other space missions (i.e., Kaguya), the nine chemical elements presented in the *Prettyman et al. (2006)* dataset is processed in a consistent way and have been cross-calibrated against the range of Apollo and lunar meteorite sample compositions (see also *Gillis et al., 2004, Warren 2005*). *Prettyman et al. (2006)* also report 1σ uncertainties per pixel for every element. These data were rebinned into 0.5° per pixel dataset (equivalent to 15 × 15 km square pixels) –see Supplementary Figure 15.

Chi-squared analysis: Here we apply a chi-squared analysis to identify regions of the LP-GRS chemical dataset that are closest in composition to a give lunar sample composition, taking

into consideration elements that have more accurately measured errors. This approach, given by Equation 1 below, takes into consideration:

i. The Lunar Prospector elemental or oxide abundance per pixel.
ii. The Lunar Prospector uncertainty in the elemental or oxide abundance per pixel.
iii. The sample's bulk chemistry elemental or oxide abundance.
iv. The uncertainties on the sample's bulk chemistry elemental or oxide abundances.

$$x^2 = \sum_{i=0}^{n} \left[\frac{(e_i + r_i)}{\left(\sigma_{e_i}^2 + \sigma_{r_i}^2\right)} \right]^2 \qquad \text{(Equation 1)}$$

Where *n* = number of chemical elements, e = chemical element, r = region of interest (i.e., the Lunar Prospector pixel value for that element), and σ = one sigma error on the LP-GRS pixel and sample compositions.

A probability (P-index value) can then be calculated to assess the probability of the result being due to chance alone (i.e. just measurement noise) given the measured X^2 distribution for *n* numbers of elements (i.e., degrees of freedom *d*), under the null-hypothesis that a remotely sensed pixel has a chemical composition that is equivalent to the meteorite sample. This is determined by:

$$1 - \text{cdf}\ (x^2,\ \text{n}) \qquad \text{(Equation 2)}$$

These equations were coded in the IDL programming language using (i) compositions + (ii) uncertainty inputs from the HIGH1 dataset rebinned into a 720 × 360 equal cylindrical projection grid, inputs from the (iii) sample composition + (iv) uncertainty values. The x^2 value is calculated using the inbuilt CHISQR_PDF function (which computes the probability that a variable from the chi-square distribution with *d* degrees of freedom [where X is the number of elements], is greater than the x^2 value. P-value probability results (on a scale from 0-1) are visually displayed as maps using a modified version of visual display routines originally developed by the Lunar Prospector mapping team. The higher the P-value, the higher the probability that there is a match between the input sample composition and the LP-GRS elemental dataset. Note that the maps reported in Supplementary Figures 17, 18 and 19 have been overlain as a slightly transparent layer on top of Clementine albedo map of the Moon so that the reader is able to geographically contextualise the data. However, this might have altered slightly the illustrated colour scale per pixel.

Potential issues with this approach: Before reporting our results, a quick discussion highlighting potential issues is needed to set the scene.

Firstly, we must consider the appropriateness of comparing a geological sample that is a few grams to hundreds of grams (i.e., a very small spatial scale) with a 60 × 60 km region of the Moon. One can argue that this is not an appropriate comparison to make, however, until we have higher spatial resolution elemental datasets to undertake such analysis it is the best we have to work with to achieve our goal of investigating the likely geological context of lunar meteorite source regions.

Secondly, we must consider the accuracy and precision of LP-GRS dataset (see discussion above). The reported 1 sigma uncertainties are shown in comparison to the reported element concentrations in Supplementary Figures 15 and 16. The stripes in elemental uncertainty maps (Supplementary Figure 15 middle panels) originate from estimated uncertainties in the input spectra used in the spectral unmixing analysis of *Prettyman et al. (2006).* The input data were produced from raw data 32 second binned data by *Lawrence et al. (2004*), with some corrections to minimise systematic errors. For spectral unmixing of the gamma-ray data, *Prettyman et al. (2006)* assumed that the counting data are Poisson random variates (analysis presented in *Lawrence et al., 2004*). The propagated statistical uncertainty for each element and pixel was calculated using a covariance matrix as described in *Prettyman et al. (2006).* The data are gridded to calculate a quasi-equal grid pattern, and since Lunar Prospector was in a circular polar orbit, pixels at the poles received many more hits, which is why for longitudes >61 S and >63 °N (i.e., sub-polar to polar regions) pixel counting statistics are better and uncertainties are lower for many elements at the poles (Supplementary Figure 15). An increase in the scatter and range of the LP-GRS data when rebinned from 20° per pixel to 2° per pixel is accompanied by a reduction in precision.

The abundances of Th, FeO, TiO_2, and K are considered to be the most reliably determined elements at the 2° per pixel spatial scale, having uncertainties that are well correlated with their reported concentrations (Supplementary Figure 16). However, we note that % error uncertainties are very large (often >100%) for low concentrations of these elements; for example, for Th, TiO2 and K in the lunar highlands and mare regions outside of the Procellarum KREEP Terrane (Supplementary Figure 15). The major elements SiO2, MgO, CaO, and Al_2O_3 all have poorly correlated elemental abundances and uncertainties (Supplementary Figure 16), although as these species relate to major rock forming components, their % errors do not typically exceed 100%, with the exception of Al_2O_3 in mare basalt regions and MgO in the lunar highlands (Supplementary Figure 15). Uranium has been

calculated as a function of its proportionality to Th abundance (Prettyman et al., 2006) and, like Th, has very large uncertainties (often >100% % errors) for pixels with low concentrations in the lunar highlands outside of the Procellarum KREEP Terrane (Supplementary Figure 15).

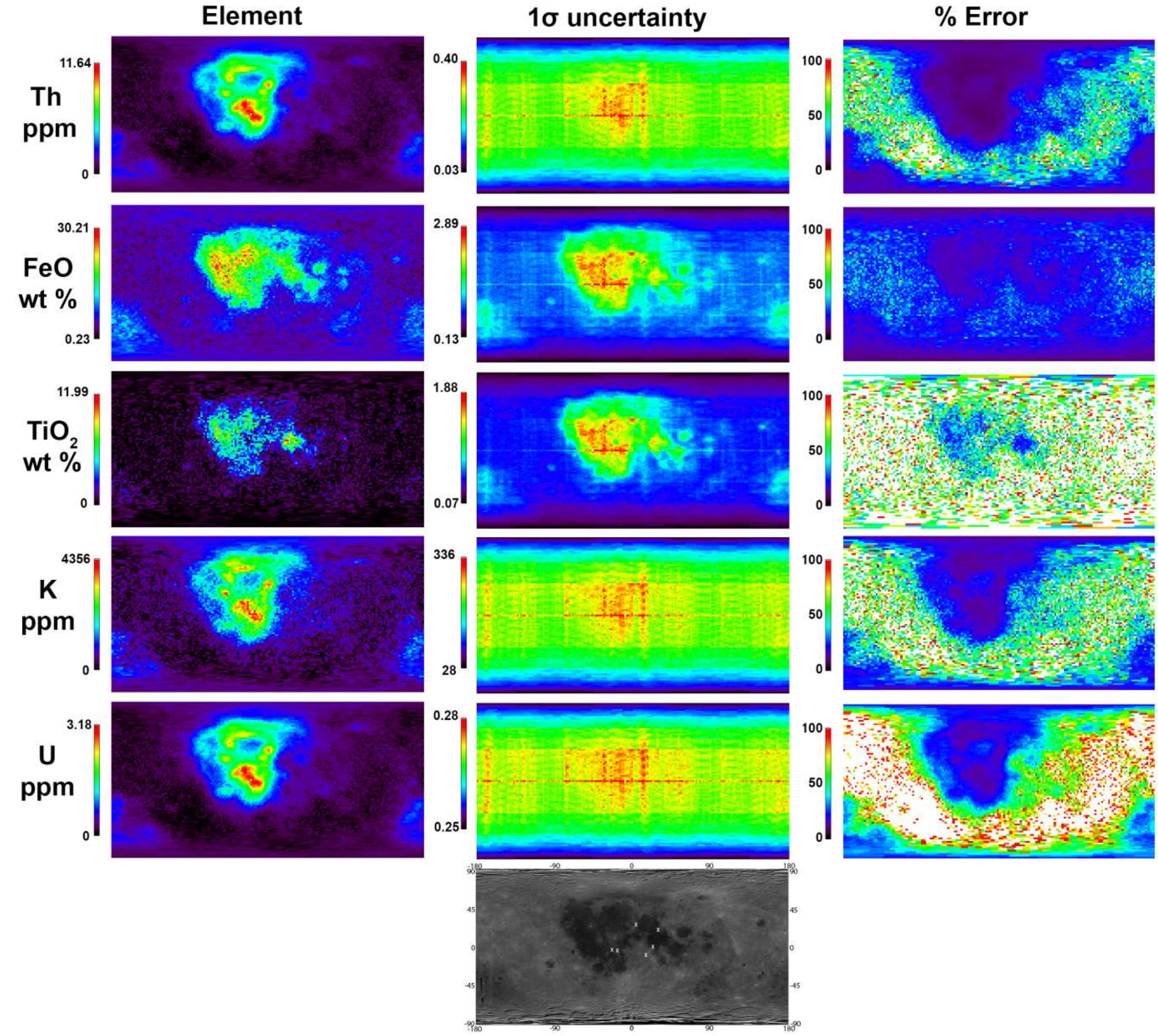

Supplementary Figure 15. Lunar Prospector element (ppm) or oxide (wt %) abundances (left column), reported one sigma uncertainties (middle column), and % error (right column), where % error > 100 are denoted as white pixels. Values are displayed in a cylindrical projection with the lunar nearside 0° longitude at centre. Lowest frame shows a Clementine mission albedo map of the Moon with locations of the six Apollo landing sites denoted as crosses for context.

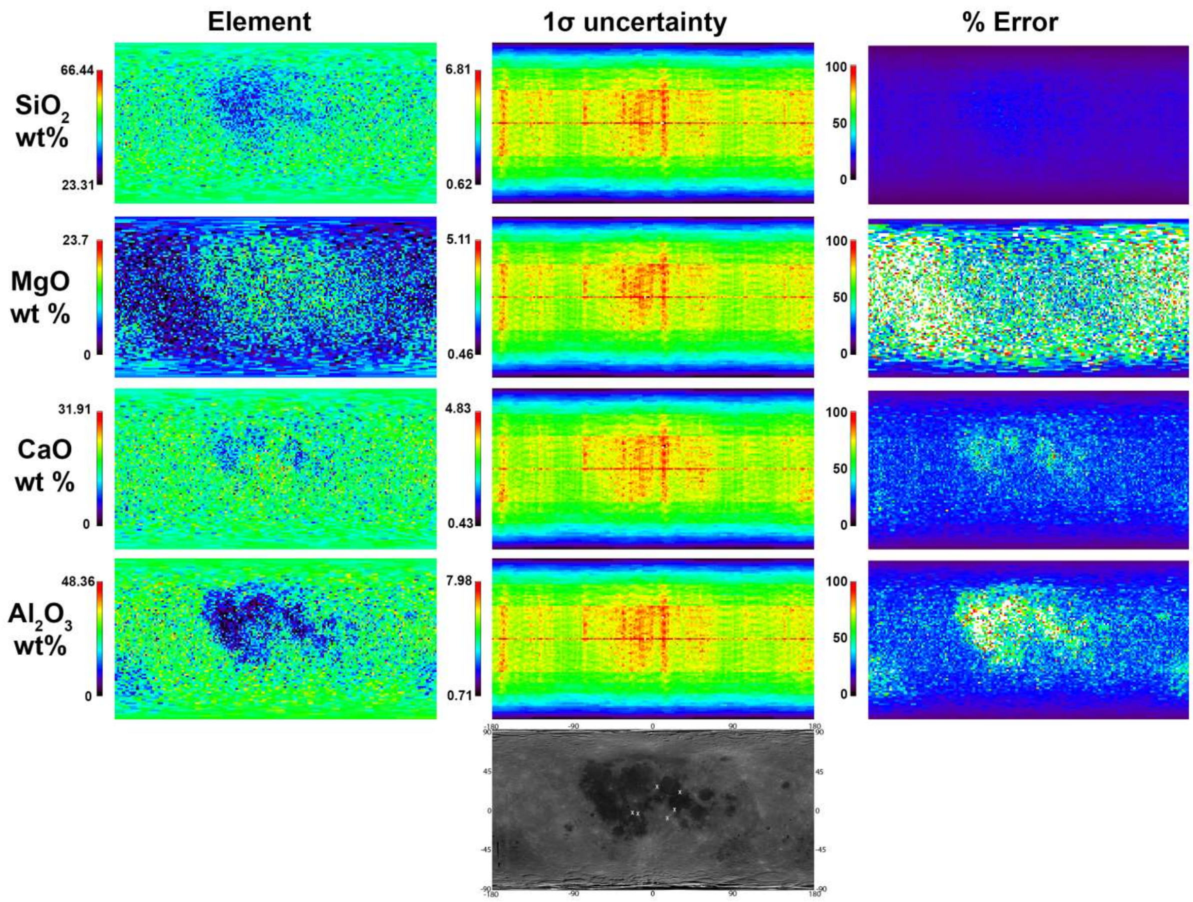

Supplementary Figure 15. (b) continued for SiO_2, MgO, CaO and Al_2O_3 wt%.

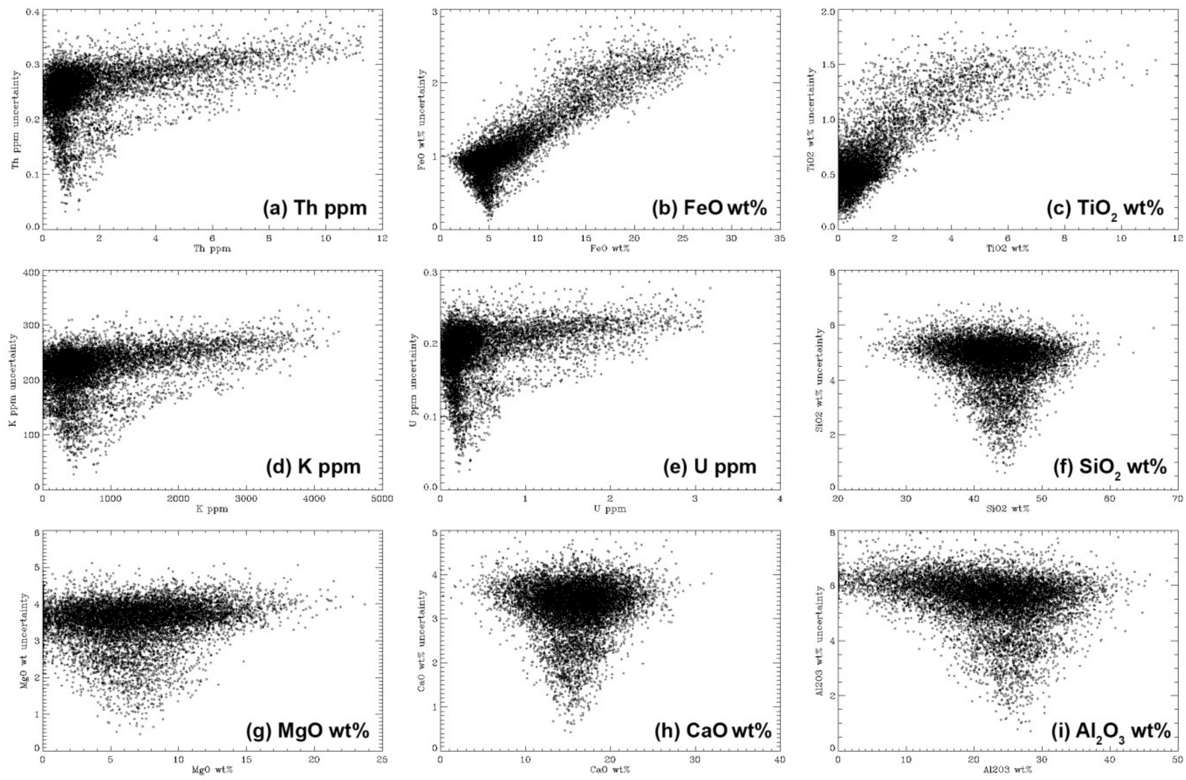

Supplementary Figure 16. Comparison of LP-GRS element (ppm) or oxide (wt %) abundances and their reported one sigma uncertainties (see text for details).

Thirdly, we must consider the accuracy and precision of the lunar sample composition we are interested in comparing with the remote sensing dataset. Depending on the approach used, we might choose to generate an “average sample composition” using data from different analytical approaches that use different sample masses, resulting in different levels of analytical uncertainty on different elements. We must also consider the robustness of the data reported from different laboratories, and if all the elements needed to make the comparison have been reported (for example, some techniques will only report major elements but not traces, others might only report one or two major elements, but all the trace elements). Therefore, how we consider the uncertainties on the “average sample composition” becomes important (see further discussion below). Alternatively, if only one analysis has been made on the sample of interest, an average composition cannot be calculated, and instrumental analytical uncertainties, if available, need to be considered to undertake the chi-squared analysis.

4.2 Validation using Apollo soils

We first tested our approach by comparing the average compositions of the Apollo returned samples bulk soils to the LP-GRS chemical dataset. The values in Supplementary Table 17 are from our own calculated landing site bulk soil chemistries using just the Apollo soil data

reported in the literature, and do not include Apollo regolith breccia data. Our rationale for this is that the regolith breccias often represent ancient (fossil) soils (*McKay et al., 1986*), and, thus, do not necessarily represent the composition of the present-day Apollo landing sites that are measured from orbit.

The number of analyses considered in this average landing site calculation is different for different elements (see Supplementary Table 17), depending on if that data has been reported commonly in the literature or not. As such, we have considered three different types of uncertainty 'errors' (where these errors are not true analytical errors as these were traditionally not reported in the Apollo era literature, but instead represent the compositional variability of the landing site as determined by the spread of data included in the average analysis) on the average landing site composition:

i. Error method 1: The one standard deviation on the values used to calculate a mean composition.
ii. Error method 2: The 95% confidence interval for the mean (i.e., the student T-test approach s*t/SQRT(N), where s is the one standard deviation on the values used to calculate a mean composition, t is Student's t-factor and n is the number of variables used to calculate a mean composition; see approach of *Korotev (2012), and Korotev and Irving (2021)* used to calculate uncertainties on lunar meteorite bulk compositions).
iii. Error method 3: An estimate of the error on the mean, assuming that the individual measurements all have the same error and the error is approximately equal to the spread of the data.

Supplementary Table 17. Calculated average Apollo landing site compositions with reported errors using the three methods outlined in the text above. Th, U, and K are reported in ppm, the other elements in oxide wt%.

	SiO_2	Al_2O_3	FeO	MgO	CaO	TiO_2	Th	U	K	Oxide sum
A11 Av.	41.77	13.39	15.74	8.28	12.03	7.62	1.28	0.51	1183.0	98.82
n.	4	4	4	4	4	4	4	2	4	
Error method 1 (1 stdev)	0.32	0.21	0.27	0.95	0.06	0.09	0.76	0.01	104.5	
Error method 2 (Student T-test)	0.52	0.34	0.43	1.51	0.10	0.14	1.21	0.06	166.2	
Error method 3 (error on the mean)	0.16	0.11	0.13	0.48	0.03	0.04	0.38	0.01	52.2	
A12 Av.	46.16	13.92	15.37	9.76	10.63	3.11	6.54	1.75	2414.4	98.95
n.	12	13	14	13	13	13	8	6	12	
Error method 1 (1 stdev)	0.88	1.15	1.33	0.59	0.25	0.53	1.96	0.75	767.0	
Error method 2 (Student T-test)	0.56	0.70	0.77	0.35	0.15	0.32	1.64	0.79	487.3	
Error method 3 (error on the mean)	0.25	0.32	0.36	0.16	0.07	0.15	0.69	0.31	221.4	
A14 Av.	47.96	17.36	10.38	9.42	10.85	1.72	13.60	3.44	4462.1	97.69
n.	8	9	10	8	8	9	7	5	8	
Error method 1 (1 stdev)	0.40	0.42	0.32	0.17	0.43	0.08	1.04	0.48	257.8	

Error method 2 (Student T-test)	0.34	0.33	0.23	0.14	0.36	0.06	0.96	0.60	215.5	
Error method 3 (error on the mean)	0.14	0.14	0.10	0.06	0.15	0.03	0.39	0.22	91.1	
A15 Av.	46.69	14.55	14.18	11.16	10.81	1.49	3.70	1.17	1472.2	98.86
n.	21	25	48	24	25	25	8	6	23	
Error method 1 (1 stdev)	0.97	2.69	2.72	1.38	0.85	0.34	1.42	0.47	381.6	
Error method 2 (Student T-test)	0.44	1.11	0.79	0.58	0.35	0.14	1.19	0.49	165.0	
Error method 3 (error on the mean)	0.21	0.54	0.39	0.28	0.17	0.07	0.50	0.19	79.6	
A16 Av.	45.00	27.31	5.00	5.72	15.75	0.54	1.83	0.51	1213.2	99.32
n.	26	43	46	42	44	39	30	25	29	
Error method 1 (1 stdev)	0.36	1.07	0.68	0.87	0.75	0.09	0.61	0.16	1242.7	
Error method 2 (Student T-test)	0.14	0.33	0.20	0.27	0.23	0.03	0.23	0.07	472.7	
Error method 3 (error on the mean)	0.07	0.16	0.10	0.13	0.11	0.01	0.11	0.03	230.8	
A17 Av.	42.75	16.13	13.01	10.11	11.76	4.96	1.77	0.70	1188.9	98.73
n.	33	33	77	33	33	33	11	15	33	
Error method 1 (1 stdev)	2.09	4.14	3.47	0.59	1.07	3.16	1.06	0.64	1161.7	
Error method 2 (Student T-test)	0.74	1.47	0.79	0.21	0.38	1.12	0.71	0.35	411.9	
Error method 3 (error on the mean)	0.36	0.72	0.40	0.10	0.19	0.55	0.32	0.16	202.2	

In Supplementary Table 18, we compare our average soil element abundances from each Apollo landing site to the composition of each LP-GRS pixel that includes that landing site. Relative differences are highly variable element to element and landing site to landing site, with 53% of the Apollo landing site elemental values being outside of the range of the LP-GRS value ± 1σ error. Thus, we should not expect our search of the LP-GRS dataset for pixels most like the Apollo landing site compositions to yield perfect matches.

Supplementary Table 18. Table showing LP-GRS (i) chemical abundances and (ii) 1σ errors (as plotted in maps in Supplementary Figure 15) for each Apollo landing site location on the Moon (Prettyman et al., 2006), (iii) the Apollo landing site compositions we have calculated from reported soil data (see Supplementary Table 17), (iv) a Z-score to assess if the calculated Apollo landing site data seen in (iii) are within the error range (ii) of the LP-GRS data (i), and (v) the relative difference between the LP-GRS data (i) and the calculated Apollo soil data (iii).

	**Th**	**FeO**	**TiO_2**	**K**	**U**	**SiO_2**	**MgO**	**CaO**	**Al_2O_3**	**Oxide sum**
**(i) Lunar Prospector Landing Site Pixel Chemistry**										
A11	1.49	12.23	3.44	1132.30	0.41	43.93	10.54	9.66	15.30	95
A12	8.84	15.90	3.02	2987.20	2.41	40.67	14.19	11.30	15.75	101
A14	10.74	15.55	1.21	4041.10	2.93	35.63	12.65	21.24	21.24	108
A15	5.44	14.23	1.56	2328.00	1.48	38.66	9.96	21.53	16.33	102
A16	2.45	5.69	0.00	903.84	0.67	43.15	7.81	16.93	22.80	96
A17	1.69	10.10	3.65	607.43	0.46	39.25	12.02	4.79	25.20	95
**(ii) Lunar Prospector Pixel Landing Site Chemistry reported 1σ error**										
A11	0.29	1.96	1.20	254.02	0.23	5.40	3.99	3.92	6.40	
A12	0.32	2.25	1.44	266.75	0.23	5.42	4.11	3.78	6.36	
A14	0.31	2.15	1.36	260.93	0.22	5.30	4.03	3.66	6.24	
A15	0.30	1.73	1.01	260.07	0.22	5.46	4.14	3.85	6.42	
A16	0.28	1.22	0.66	240.04	0.21	5.18	3.82	3.63	6.03	
A17	0.30	1.89	1.16	258.47	0.23	5.50	4.10	3.98	6.53	

**(iii) Apollo landing site average soil compositions (see Supplementary Table 17)**										
A11	1.28	15.74	7.62	1183	0.51	41.77	8.28	12.03	13.39	99
A12	6.54	15.37	3.11	2714	1.75	46.16	9.76	10.62	13.92	99
A14	13.6	10.38	1.72	4462	3.44	47.96	9.42	10.85	17.36	98
A15	3.7	14.18	1.49	1472	1.17	46.69	11.16	10.81	14.55	99
A16	1.83	5	0.54	1213	0.51	45	5.72	15.75	27.31	99
A17	1.77	13.01	4.96	1188	0.7	42.75	10.11	11.76	16.13	99
**(iv) Z-score**										
A11	-0.73	1.79	3.48	0.20	0.46	-0.40	-0.56	0.60	-0.30	
A12	-7.20	-0.23	0.06	-1.02	-2.90	1.01	-1.08	-0.18	-0.29	
A14	9.13	-2.40	0.38	1.61	2.33	2.33	-0.80	-2.84	-0.62	
A15	-5.75	-0.03	-0.07	-3.29	-1.41	1.47	0.29	-2.78	-0.28	
A16	-2.21	-0.56	0.82	1.29	-0.75	0.36	-0.55	-0.32	0.75	
A17	0.26	1.54	1.13	2.25	1.04	0.64	-0.47	1.75	-1.39	
**(v) % difference between LP data and Apollo landing site soil data**										
A11	14.2	-28.7	-121.3	-4.5	-25.6	4.9	21.4	-24.5	12.5	
A12	26.0	3.3	-2.9	9.1	27.4	-13.5	31.2	6.1	11.6	
A14	-26.7	33.2	-42.0	-10.4	-17.4	-34.6	25.5	48.9	18.3	
A15	32.0	0.3	4.4	36.8	21.1	-20.8	-12.1	49.8	10.9	
A16	25.2	12.1		-34.2	23.5	-4.3	26.8	6.9	-19.8	
A17	-4.6	-28.9	-36.0	-95.6	-51.8	-8.9	15.9	-145.6	36.0	
Min	*-26.7*	*-28.9*	*-121.3*	*-95.6*	*-51.8*	*-34.6*	*-12.1*	*-145.6*	*-19.8*	
Max	*32.0*	*33.2*	*4.4*	*36.8*	*27.4*	*4.9*	*31.2*	*49.8*	*36.0*	

Supplementary Table 19 summarises the P-value results for the LP-GRS pixel containing each Apollo landing site input average soil compositions using the three different error approaches and different combinations of LP-GRS elements, with scenario 1 taking into account only FeO, TiO_2 and Th, and scenario 2 taking into account all nine elements. Maps of the P-value results obtained using scenario 1 and 2 are shown in Supplementary Figures 17 and 18, respectively.

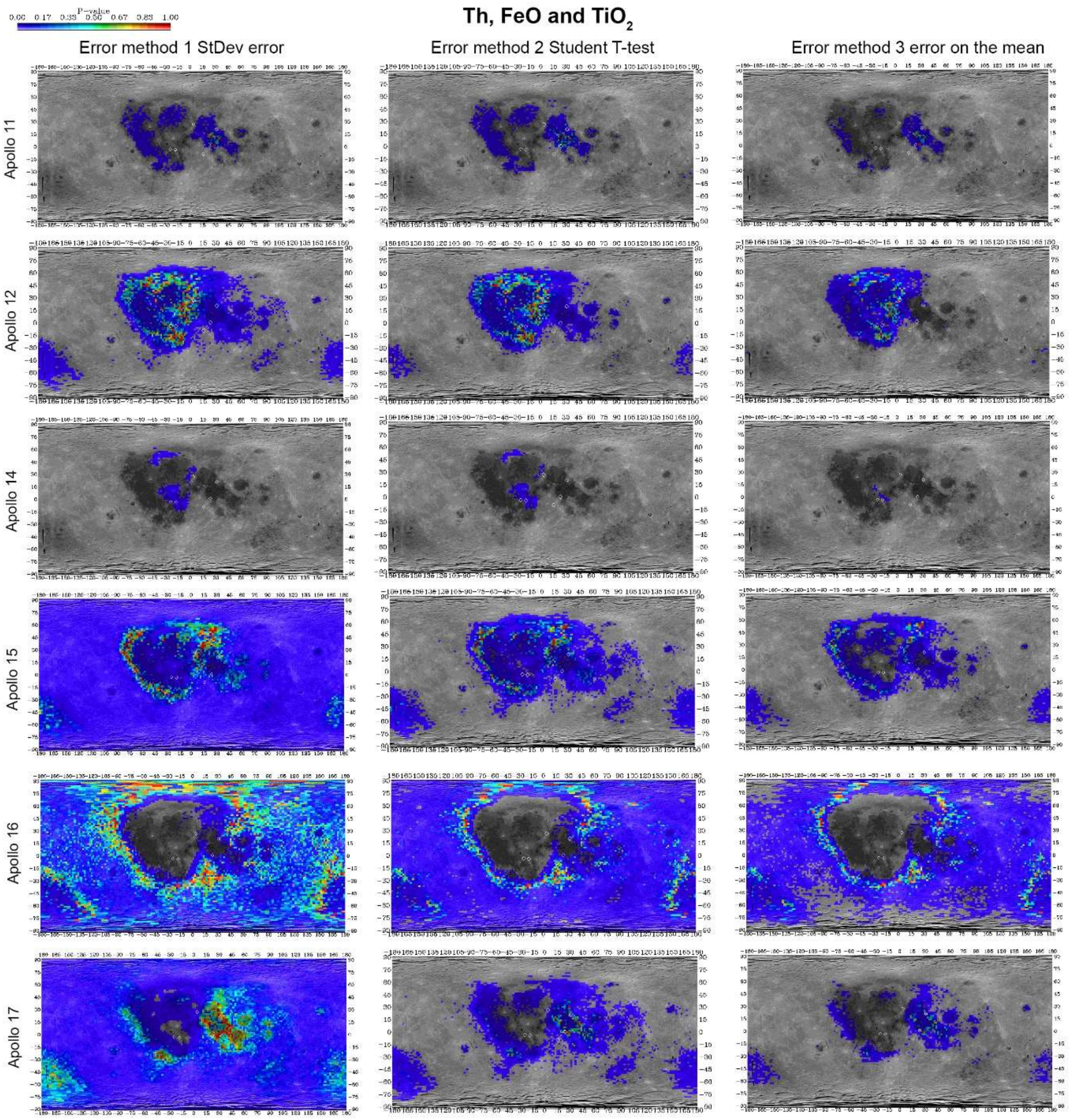

Supplementary Figure 17. Probability maps of Apollo soil compositions being a match to LP-GRS Th, FeO, and TiO_2 abundances. Three different search parameters are depicted, Apollo samples ± error method 1 (left column), Apollo samples ± error method 2 (central column), and Apollo samples ± error method 3 (right column). The pixel probabilities are coloured on a 20 point colour scale where red pixels are highest probability, and blue are low probability. Pixels where the P-index = 0 have been removed from the maps. Apollo landing sites are denoted by white diamonds in each map.

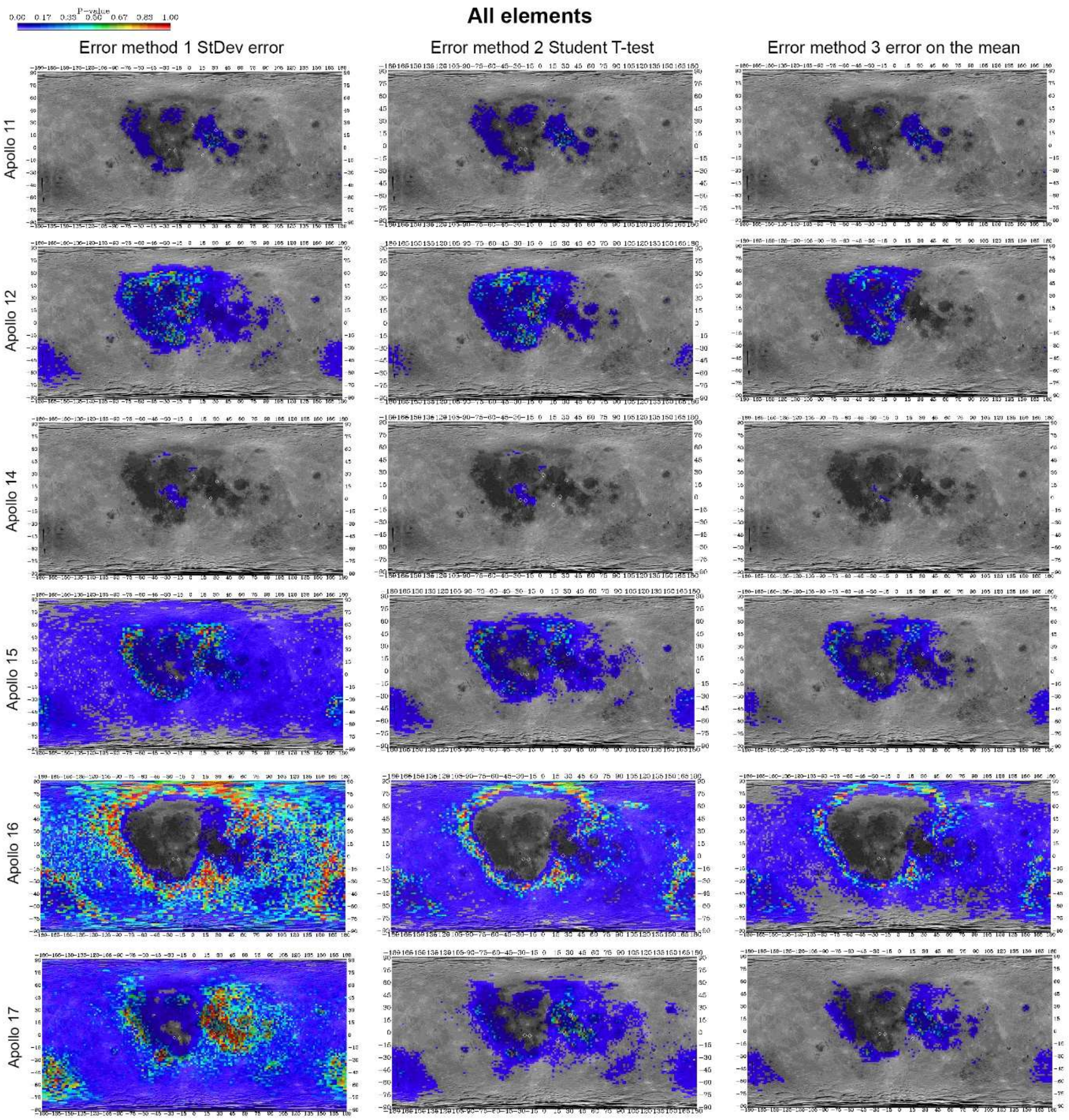

Supplementary Figure 18. Probability maps of Apollo soil compositions being a match to all nine LP-GRS element and oxide abundances. Three different search parameters are depicted, Apollo samples ± error method 1 (left column), Apollo samples ± error method 2 (central column), and Apollo samples ± error method 3 (right column). The pixel probabilities are coloured on a 20 point colour scale where red pixels are highest probability, and blue are low probability. Pixels where the P-index = 0 have been removed from the maps. Apollo landing sites are denoted by white diamonds in each map.

Finally, we looked at the specific P-value at each of the pixels that overlays a specific Apollo landing site (Supplementary Table 19).

Supplementary Table 19. Summary of P-value results of each Apollo landing site equivalent pixels on the Moon using the different input error search parameters where error method 1 = one standard deviation, error method 2 = student T-test, and error method 3 = estimate of the error on the mean.

**Error method 1**	**A11**	**A12**	**A14**	**A15**	**A16**	**A17**
FeO, TiO_2 and Th	0.001588	0.710222	0.005233	0.696618	0.626045	0.872994
All elements	0.058126	0.878659	0.00057	0.0953	0.955074	0.753486
**Error method 2**	**A11**	**A12**	**A14**	**A15**	**A16**	**A17**
FeO, TiO_2 and Th	0.001763	0.584902	0.003058	0.570365	0.274007	0.440591
All elements	0.063197	0.824066	0.000367	0.017469	0.758013	0.35565
**Error method 3**	**A11**	**A12**	**A14**	**A15**	**A16**	**A17**
FeO, TiO_2 and Th	0.001426	0.027007	0	0.031248	0.15731	0.340422
All elements	0.053764	0.089435	0	0.000448	0.56391	0.171911

It is clear from these maps (Supplementary Figures 17 and 18) and data (Supplementary Table 19) that the search using the Apollo landing site data ± error method 1 (standard deviation) produces the most pixel matches (i.e., the least constrained search results). The search using the Apollo landing site data ± error method 2 (student T-test) yield results that are most tightly constrained. Searches using the Apollo landing site data ± error method 3 produces results that rules out the most pixel searched. Using all the nine elements produces more constrained results than just the searches using FeO, TiO_2 and Th. On this basis, we recommend using an approach where ideally all nine elements are used as the chemical input, and use (where possible) the ± error method 2 (student T-test) for the sample uncertainties.

4.3 Lunar Meteorites

To identify regions of interest on the Moon's surface that are compositionally similar to lunar meteorites, we need to make some assumptions. These include:

(1) Lunar meteorites are ejected from near surface environments, within the upper few meters of the lunar regolith *(Lorenzetti et al., 2005*) that represents a similar depth as accessible by the Lunar Prospector gamma-ray spectrometer (*Prettyman et al., 2006*).

(2) The present-day composition of the lunar surface as mapped by LP-GRS is representative of the upper few metres of the lunar regolith.

(3) Lunar meteorites best suited for this type of source provenance analysis are heterogeneous regolith breccias or fragmental breccias that have been well mixed, potentially representing the local soil composition from where they were sourced.

If we accept these conditions, then the question then becomes what uncertainty values to use for the search. Some lunar meteorites have been analysed across different laboratories using different analytical techniques that report different error levels and required different mass samples. However, other samples might have only been looked at by a single lab and so no inter-lab average composition is available. Some samples could have been analysed as a powdered rock (i.e., taking a few tens or hundreds of mg of a sample and crushing it up to homogenise it and then run repeat analysis on the same parent powder) (e.g*., Joy et al., 2010*), others might have taken a similar mass sized sample and broken it up into several small chips which where each analysed in turn (e.g., *Korotev, 2012; Korotev et al., 2009; Korotev and Irving, 2021*). Both these approaches can result in different types of reported errors (analytical uncertainty and/or indication of sample heterogeneity). Thus, if future works want to make the type of searches we discuss here, they must be clear about what bulk chemistry data and type of error reporting has been used to undertake the analysis.

4.4 Searches using Randy Korotev's lunar meteorite data

Dr Randy Korotev, Washington University St Louis, has published several compilation of lunar meteorite bulk chemistry data, collected using the Instrumental neutron activation analysis (INAA) analytical technique for minor and some major elements, and fused bead electron probe microanalysis (EPMA) analysis for major elements. His approach for INAA is to analyse several small fragments of the same meteorite stone and use the mass weighted mean to represent the bulk composition of the meteorite. The fused bead method is conducted by taking some of the INAA rock chips, pulverising them, and fusing them into a single glass bead for analyse (e.g., *Korotev et al. 2009; Korotev, 2012*). For the purpose of this study, we used his data as they are reported in a consistent way where the errors are often reported both as ±1 standard deviation analytical errors, or more commonly as the ±95% confidence interval for the mean as a measure of the sample heterogeneity. Compositional data used for the NWA 2995 search analysis are listed in Supplementary Table 20.

Supplementary Table 20. Bulk composition data for the NWA 2995 meteorite reported by *Korotev et al. (2009).* Data are mass weighted mean. Errors reported are the 95% confidence interval of the mean (i.e., error method 2 described above).

**SiO_2**	wt%	46.20	±	0.924
**TiO_2**	wt%	0.68	±	0.0136
**Al_2O_3**	wt%	20.60	±	0.412
**MgO**	wt%	8.08	±	0.1616

**CaO**	wt%	13.50	±	0.27
**FeO**	wt%	9.75	±	0.195
**K**	ppm	1577	±	79
**U**	ppm	0.42	±	0.04
**Th**	ppm	1.55	±	0.14

Probability search maps for NWA 2995 using the input composition in Supplementary Table 20can be seen in Supplementary Figure 19 (and see Figure 4 in the main paper) where the search has been carried out using all nine available LP-GRS elements (right panel), and only Th, FeO, and TiO_2 (left panel).. The data are arranged in a grid of 360 rows (1 row per per 0.5 degree latitude) and 720 (1 column per 0.5 degree longitude), with the P-index value for that location listed per cell.

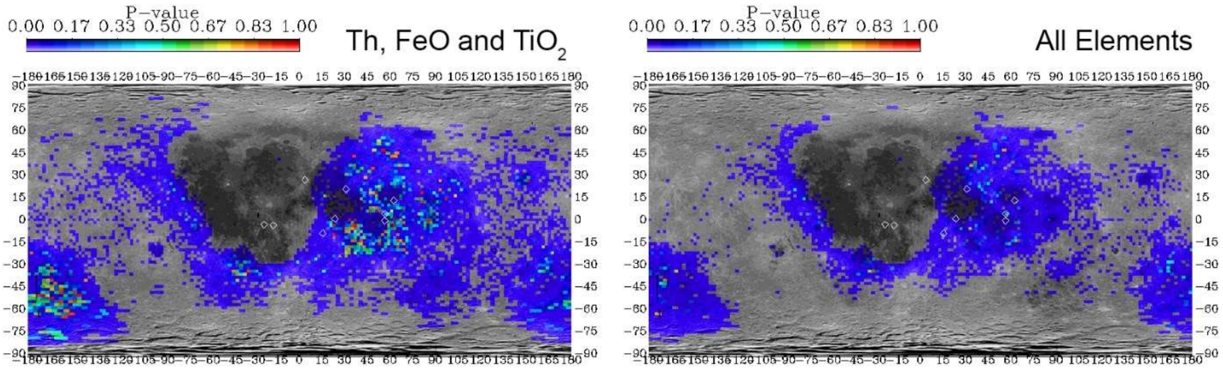

Supplementary Figure 19. Probability (P-index) maps of NWA 2995 bulk rock compositions ± error method 2 (student T-test) being a match to LP-GRS Th, FeO, and TiO_2 abundances (left) and all LP-GRS elements (right: data in Supplementary Table 15). The pixel probabilities are coloured on a 20 point colour scale where red pixels are highest probability, and blue are low probability. Pixels where the P-index = 0 have been removed from the maps. Apollo landing sites are denoted by white diamonds in each map.

Supplementary Table 21. Ranked list of high probability (p=>0.5) 0.5° pixels on the Moon where the bulk composition of NWA 2995 are most similar to the composition of the lunar surface (all nine elements) mapped by Lunar Prospector (see Figure 4 of the main paper and Supplementary Table 16). Ages of the underlying geological surfaces are indicated from either the Digital Unified Global Geologic Map of the Moon At 1:5,000,000 scale (*Fortezzo et al. 2020*) or as determined from crater counting via various authors. The two regions of interest associated with the Pre-Nectarian terrane around Cabannes crater (Figure 4 main paper) are indicated in bold font.

Rank	P-value	Lat min	Lat max	Lon min	Lon Max	Location	Region	Expected dominant rock / age	Surface age	Surface age reference	Unit age	Unit age reference
1	0.8682443	42.5	41.5	36	38	N of Lacus Somniorum	Eastern mare-highlands boundary (N)	VLT mare surface, Post-Imbrian age	On top of Imbrian plains material, adjacent to VLT basalts	as mapped by Fortezzo et al. (2020)		
2	0.8560345	-13	-14.5	65	67	Lame crater	Eastern mare-highlands boundary (S)	Crater ejecta, Imbrian age	Crater (Imbrian plains age) which is overprinting Vendelinus crater which is lower Imbrian age, and younger than Langrenus which is Erstanthian in age	as mapped by Fortezzo et al. (2020)		
3	0.8052577	-33.5	-35	-32	-30	Palus Epidemiarum	Periphery PKT	Mare basalts	Mare surface		2.39 Ga ? Very few studies, no modern study available	as mapped by Baldwin (1970)
**5**	**0.7622789**	**-61.5**	**-62.5**	**-175**	**-173**	**Cabannes**	**SPA**	**Crater floor (SPA compositional anomaly Ca-pyx), pre-Nectarian age**	**Pre-Nectarian**	**as mapped by Fortezzo et al. (2020)**		
**4**	**0.7622789**	**-61.5**	**-62.5**	**-173**	**-171**	**Cabannes**	**SPA**	**Crater floor (SPA compositional anomaly Ca-pyx), pre-Nectarian age**	**Pre-Nectarian**	**as mapped by Fortezzo et al. (2020)**		
6	0.7606359	-39.5	-40.5	-170	-168	S of Oppenheimer (N of Maksutov)	SPA	Crater ejecta from Oppenheimer			4.04 Ga	crater age from Gaddis et al. (2017)
7	0.7516856	46.5	45.5	55	57	Lacus Temporis	Eastern mare-highlands boundary (N)	VLT mare surface			3.66- 3.74 Gyr ago	Hiesinger et al. (2010)
8	0.712729	36.5	35.5	33	35	Lacus Somniorum	Eastern mare-highlands boundary (N)	VLT mare surface, Post-Imbrian age	VLT mare surface	as mapped by Fortezzo et al. (2020)		
9	0.7090176	-33.5	-34.5	-162	-160	NW of Oppenheimer	SPA	Crater ejecta from Oppenheimer			4.04 Ga	Hiesinger et al. (2010)
10	0.6771237	-33.5	-35	-35	-33	Palus Epidemiaum	Periphery PKT	Mare basalts	Mare surface		2.39 Ga ? Very few studies, no modern study available	as mapped by Baldwin (1970)

11	0.6541883	-5.5	-6.5	33	34	Isodorus E	Eastern mare-highlands boundary (S)	Nectarian aged unit		as mapped by Fortezzo et al. (2020)		
12	0.6485393	38.5	37.5	36	38	Lacus Somniorum	Eastern mare-highlands boundary (N)	VLT mare surface, Post-Imbrian age	VLT mare surface	as mapped by Fortezzo et al. (2020)		
13	0.5826848	-19.5	-20.5	179	180	Racah N	SPA	Crater ejecta, imbrian age	Imbrian age craters	as mapped by Fortezzo et al. (2020)		
14	0.536833	14.5	13.5	-78	-76	Cardanus M	Periphery PKT	Crater ejecta / adjacent to ~3.3-3.4 Ga low-Ti mare basalt units	Imbrian Orientale Heveluis formation - Orientale 2ry ejecta	as mapped by Fortezzo et al. (2020)		
15	0.5322137	12.5	11.5	47	48	Glaisher W	Eastern mare-highlands boundary (S)	Crisium ejecta blanket	Crisium age	as mapped by Fortezzo et al. (2020)		
16	0.5245107	4.5	3.5	57	58	South of Apollonis A crater, Luna 20 site area	Eastern mare-highlands boundary (S)	Crisium ejecta blanket	Crisium age	as mapped by Fortezzo et al. (2020)		
17	0.5211514	-37.5	-38.5	173	175	NW of Leibnitz	SPA	Ejecta adjacent to VLT/Low-Ti mare basalts	Mare basalt in crater older than the Imbrium basin, but younger than the Nectaris basin.		3.37-3.68 Ga	Pasckert et al. (2018)
18	0.5164981	42.5	41.5	51	53	S of Lacus Temporis (near Shuckburgh crater)	Eastern mare-highlands boundary (N)	Crater floor, Imbrian age	Crater floor, Imbrian age	as mapped by Fortezzo et al. (2020)		
19	0.5009696	-41.5	-42.5	-170	-168	Maksutov	SPA	Mare basalt	Mare surface (3.06 Ga) within 3.25-3.71 Ga crater		3.06 Ga	Pasckert et al. (2018)

Supplementary Note 1 References

Baldwin, R.B., 1970. Absolute ages of the lunar maria and large craters: II. The viscosity of the moon's outer layers. *Icarus*, *13*(2), pp.215-225.

Calzada-Diaz, A., Joy, K.H., Crawford, I.A. and Nordheim, T.A., 2015. Constraining the source regions of lunar meteorites using orbital geochemical data. *Meteorit. Planet. Sci.*, *50*(2), pp.214-228.

Fortezzo, C.M., Spudis, P. D. and Harrel, S. L. 2020. Release of the Digital Unified Global Geologic Map of the Moon At 1:5,000,000 Scale. Paper presented at the 51st Lunar and Planetary Science Conference, Lunar and Planetary Institute, Houston, TX. (abstr. 2760).

Gaddis, L.R., Bennett, K., Horgan, B., McBride, M., Stopar, J., Lawrence, S., Gustafson, J.O. and Giguere, T., 2017, March. Complex Volcanism at Oppenheimer U Floor-Fractured Crater. In *Lunar and Planetary Science Conference* (No. JSC-CN-38866).

Gillis, J.J., Jolliff, B.L. and Korotev, R.L., 2004. Lunar surface geochemistry: Global concentrations of Th, K, and FeO as derived from lunar prospector and Clementine data. *Geochim. Cosmochim. Acta* , *68*(18), pp.3791-3805.

Hiesinger, H., Head III, J.W., Wolf, U., Jaumann, R. and Neukum, G., 2010. Ages and stratigraphy of lunar mare basalts in Mare Frigoris and other nearside maria based on crater size-frequency distribution measurements. *J Geophys Res-Planet*, *115*(E3).

Korotev, R.L., 2012. Lunar meteorites from Oman. *Meteorit. Planet. Sci.*, *47*(8), pp.1365-1402.

Korotev, R.L. and Irving, A.J., 2021. Lunar meteorites from northern Africa. *Meteorit. Planet. Sci.*, *56*(2), pp.206-240.

Korotev, R.L., Zeigler, R.A., Jolliff, B.L., Irvin, A.J. and Bunch, T.E., 2009. Compositional and lithological diversity among brecciated lunar meteorites of intermediate iron concentration. *Meteorit. Planet. Sci.*, *44*(9), pp.1287-1322.

Joy, K.H., Crawford, I.A., Russell, S.S. and Kearsley, A.T., 2010. Lunar meteorite regolith breccias: An in situ study of impact melt composition using LA-ICP-MS with implications for the composition of the lunar crust. *Meteorit. Planet. Sci.*, *45*(6), pp.917-946.

Joy, K.H., Burgess, R., Hinton, R., Fernandes, V.A., Crawford, I.A., et al., 2011. Petrogenesis and chronology of lunar meteorite Northwest Africa 4472: A KREEPy regolith breccia from the Moon. *Geochim. Cosmochim. Acta* , *75*(9), pp.2420-2452.

Joy, K.H., Nemchin, A., Grange, M., Lapen, T.J., Peslier, A.H., et al., 2014. Petrography, geochronology and source terrain characteristics of lunar meteorites Dhofar 925, 961 and Sayh al Uhaymir 449. *Geochim. Cosmochim. Acta* , *144*, pp.299-325.

Lawrence, D.J., Feldman, W.C., Elphic, R.C., Little, R.C., Prettyman, T.H et al., 2002. Iron abundances on the lunar surface as measured by the Lunar Prospector gamma-ray and neutron spectrometers. *J Geophys Res-Planet*, *107*(E12), pp.13-1.

Lawrence, D.J., Elphic, R.C., Feldman, W.C., Prettyman, T.H., Gasnault, O. et al., 2003. Small-area thorium features on the lunar surface. *J Geophys Res-Planet*, *108*(E9).

Lorenzetti, S., Busemann, H. and Eugster, O., 2005. Regolith history of lunar meteorites. *Meteorit. Planet. Sci.*, *40*(2), pp.315-327.

McKay, D.S., Bogard, D.D., Morris, R.V., Korotev, R.L., Johnson, P et al., 1986. Apollo 16 regolith breccias: Characterization and evidence for early formation in the mega-regolith. *J. Geophys. Res.-Sol. Ea.*, *91*(B4), pp.277-303.

Mercer, C.N., Treiman, A.H. and Joy, K.H., 2013. New lunar meteorite Northwest Africa 2996: A window into farside lithologies and petrogenesis. *Meteorit. Planet. Sci.*, *48*(2), pp.289-315.

Pasckert, J.H., Hiesinger, H. and van der Bogert, C.H., 2018. Lunar farside volcanism in and around the South Pole–Aitken basin. *Icarus*, *299*, pp.538-562.

Peplowski, P.N. and Lawrence, D.J., 2013. New insights into the global composition of the lunar surface from high-energy gamma rays measured by Lunar Prospector. *J Geophys Res-Planet*, *118*(4), pp.671-688.

Prettyman, T.H., Hagerty, J.J., Elphic, R.C., Feldman, W.C., Lawrence, D.J., et al., 2006. Elemental composition of the lunar surface: Analysis of gamma ray spectroscopy data from Lunar Prospector. *J Geophys Res-Planet*, *111*(E12).

Warren, P.H., 2005. “New” lunar meteorites: Implications for composition of the global lunar surface, lunar crust, and the bulk Moon. *Meteorit. Planet. Sci.*, *40*(3), pp.477-506.
